# RanBP3 Regulates Proliferation, Apoptosis and Chemosensitivity of Chronic Myeloid Leukemia Cells *via* Mediating SMAD2/3 and ERK1/2 Nuclear Transport

**DOI:** 10.3389/fonc.2021.698410

**Published:** 2021-08-24

**Authors:** Qian Li, Zhenglan Huang, Yuhang Peng, Xin Wang, Guoyun Jiang, Teng Wang, Ke Mou, Wenli Feng

**Affiliations:** ^1^Key Laboratory of Laboratory Medical Diagnostics Designated by the Ministry of Education, Department of Clinical Hematology, Chongqing Medical University, Chongqing, China; ^2^Department of Hematology, The First Affiliated Hospital of Chongqing Medical University, Chongqing, China; ^3^Department of Hematology, The Second Affiliated Hospital of Chongqing Medical University, Chongqing, China

**Keywords:** RanBP3, nuclear export, CML, imatinib, ERK1/2

## Abstract

Abnormal subcellular localization of proteins is an important cause of tumorigenesis and drug resistance. Chromosome region maintenance 1 (CRM1), the nuclear export regulator of most proteins, has been confirmed to be over-expressed in various malignancies and is regarded as an efficient target. But the potential role of the CRM1 cofactor RanBP3 (Ran Binding Protein 3) is left unrevealed in chronic myeloid leukemia (CML). Here, we first detected the level of RanBP3 in CML and found an elevated RanBP3 expression in CML compared with control. Then we used shRNA lentivirus to down-regulated RanBP3 in imatinib sensitive K562 cells and resistant K562/G01 cells and found RanBP3 silencing inhibited cell proliferation by up-regulating p21, induced caspase3-related cell apoptosis, and enhanced the drug sensitivity of IM *in vitro*. Notably, we observed that RanBP3 silencing restored imatinib sensitivity of K562 cells in NOD/SCID mice. Mechanistically, the nuclear aggregation of SMAD2/3 revealed that tumor suppressor axis (TGF-β)-SMAD2/3-p21 was the anti-proliferation program related to RanBP3 knockdown, and the decrease of cytoplasmic ERK1/2 caused by RanBP3 interference leaded to the down-regulation of anti-apoptosis protein p(Ser112)-BAD, which was the mechanism of increased cell apoptosis and enhanced chemosensitivity to imatinib in CML. In summary, this study revealed the expression and potential role of RanBP3 in CML, suggesting that targeting RanBP3 alone or combined with TKIs could improve the clinical response of CML.

## Introduction

Chronic myeloid leukemia (CML) is a malignant proliferative disease originated from a fusion gene *bcr-abl*, which is produced by the translocation of the *abl1* gene in chromosome 9 and the *bcr* gene in chromosome 22 (1–3). Unlike the C-ABL protein encoded by *abl1* gene, BCR-ABL protein loses its ability of nucleocytoplasmic shuttling, is mainly anchored in the cytoplasm (4, 5), and continuously activates cytoplasmic STAT5, MAPK-ERK, and PI3K-AKT signals by its high tyrosine kinase activity, resulting in promoted leukemogenesis (6, 7). Imatinib mesylate (IM), the first-line tyrosine kinase inhibitor (TKI), has been widely applied in the clinical therapy of CML patients with the advantage of effectively improving their prognoses (8, 9). Besides, new TKIs are developed for IM-resistant patients (10, 11). However, long-time IM treatment induced resistance in 20-30% of patients, CML stem cells insensitive to TKIs result in 60% recurrence of CML patients after discontinuation (12–14). More investigation is required to develop a cure for CML.

The alteration of protein subcellular location caused by abnormal nucleocytoplasmic trafficking, a common phenomenon in various cancers, is considered to be a significant mechanism in oncogenesis and therapeutic resistance (15, 16). Chromosome region maintenance 1 (CRM1), an important nuclear export receptor, which regulates nucleocytoplasmic trafficking of most proteins by recognizing their NES (nuclear export signal) amino acid sequence, is overexpressed in lots of tumor cells and is correlated with poor prognosis (17, 18). More than 241 proteins are CRM1 cargos, including the transcription factors (FOXO, β-catenin, and NF-kB), cell cycle regulators (p21Cip1, p27Kip1, and p53), tumor suppressors (INI1, BRCA1), and carcinogenic proteins (BCR-ABL) (16, 19, 20). Published studies have shown that inhibition of CRM1 in cancers could suppress cell growth, induce cell apoptosis, and improve survival for patients by directly inhibiting the protein nuclear export (21). In CML, targeting CRM1 significantly induced cell apoptosis and reversed drug resistance (20, 22, 23), suggesting that effective regulation of protein localization in CML cells may have great significance for prognosis. However, due to extensive nuclear transportation mediated by CRM1, serious off-target toxicity is inevitable when targeting CRM1 in tumors (22, 24, 25). Thus, relatively safe selective inhibitors of nuclear export (SINE) are needed.

Ran binding protein 3 (RanBP3), an important co-factor of CRM1, binds CRM1 directly and enhances its effect on nuclear transportation of cargos which possess the leucine-rich NESs (26). Considering the important role of RanBP3 in CRM1-medidated nuclear export, it is urgent to determine the effect of RanBP3 on cancer. Recently, RanBP3 has been reported as a potential regulator in melanoma biology (27), hepatocellular carcinoma (28), prostate cancer (29), and ovarian cancer (30). Moreover, RanBP3 has been reported to be a crucial downstream molecule of MAPK-ERK and PI3K-AKT signal pathways (31), and these signals are over-activated by BCR-ABL in CML. Therefore, it is important to reveal the physiological function of RanBP3 in CML. Herein, our study aimed to figure out the expression of RanBP3 and investigate its potential function and mechanism in CML. We showed that RanBP3 was highly expressed in CML and regulated cell proliferation, apoptosis, and chemosensitivity of IM by mediating the nuclear export of SMAD2/3 and ERK1/2. Moreover, RanBP3 silencing attenuated K562 cells proliferation *in vivo*, especially in IM-treated group. Taken together, our findings indicated a crucial role of RanBP3 in CML biology, and targeting RanBP3 alone or combined with TKIs could improve the clinical response of CML patients.

## Materials and Methods

### Cell Lines and Human Bone Marrow Cells

P210^BCR-ABL^ retrovirus was transduced into BaF3 and 32D cells respectively, to generate BP210 and 32DP cell lines (32). CML cells (32DP, BP210, K562, K562/G01 and KCL22) and acute myeloid leukemia cells (HL-60, NB4, and THP-1) were cultured in the RPMI 1640 medium including 10% fetal bovine serum (FBS). For the murine cells, 32D and BaF3, 10ng/ml murine IL-3 (R&D, USA) was added into the RPMI 1640 culture medium. Cell lines in this study were purchased from the Cell Culture Center of the Chinese Medical Science Academy in Shanghai.

The bone marrow (BM) samples of anemia and diagnosed CML patients from the Second Affiliated Hospital of Chongqing Medical University were collected. Patients information was shown in **Supplementary Table 1**. All patients signed informed consent before sampling. Mononuclear cells were separated following the instructions of the human bone marrow mononuclear cells (BMMCs) separation regents (Solarbio, China).

### Reagents

The inhibitors in this study include SB431542 (MCE, USA), imatinib (TargetMol, USA), and U0126 (Cell Signaling Technology/CST, USA). All of them were dissolved with DMSO at the concentration of 10mM and stored at −20°C. RPMI 1640 (Gibco, USA) with 10% FBS (Gibco, USA) was used for drug dilution.

The antibodies involved in this study were anti-phospho-c-ABL, anti-phospho-RanBP3, anti-c-ABL, anti-RanBP3, anti-CRM1, anti-phospho-ERK, anti-ERK1/2, anti-β-Actin, anti-histon3, anti-Caspase3 and anti-PARP (CST, USA). Anti-p21 and anti-SMAD2/3 (Abcam, UK). Anti p-SMAD2/3 (Santa, USA) and anti-p-Bad (Immunoway, USA).

### Short Hairpin RNA and Lentiviral Infection

The NC (shNT) and RanBP3 shRNAs (shRanBP3) were synthesized by HanBio (Shanghai, China) and packaged into lentivirus. The K562 or K562/G01 cells were seeded at 2×10^5^ per well into 6-well plates, transfected with the lentivirus at a multiplicity of infection (MOI) of 30, then added with 6μg/ml polybrene. Cell viability, cycle, and apoptosis were analyzed after infection for 72h. To determine the role of IM on shRanBP3 infected K562 and K562/G01 cell lines, cells were treated with different doses of IM for another 48h and collected for further analysis.

### Protein Extraction, Co-Immunoprecipitation and Western Blotting

The total proteins were obtained using RIPA buffer (CST, USA) containing phosphatase and protease inhibitors. Nucleocytoplasmic protein was extracted with the use of nuclear and cytoplasmic extraction kits (Thermo Fisher Scientific, USA). BCA protein assay kits (New cell & Molecular Biotech, China) were used to determine the concentration. Western blot was performed as described earlier (32). Before incubation, the primary antibodies were diluted at 1:1000, and the corresponding second antibodies were diluted at 1:5000.

For co-immunoprecipitation, total proteins were extracted using the IP lysis buffer (Thermo Fisher Scientific, USA) containing PMSF. 30μl protein A/G magnetic beads (MCE, USA) were incubated with anti-RanBP3 antibody (Cell Signaling Technology, USA) or isotype-matched control IgG at 4°C for 2h on a shaker, after that, magnetic beads were separated magnetically, washed by 1ml PBST for 4 times, and incubated another 2h with the proteins under the same condition. Then beads were separated by a magnetic grate and washed 4 times before denaturation by 2x SDS buffer at 100°C for 10min. Western blot was then used to identify the proteins.

### Real-Time PCR

1×10^6^ cells were collected for total RNA extraction with the use of 1ml TRIZOL (Takara, Japan). The PrimeScript RT reagent Kits (Takara, Japan) were used to reverse transcription of total RNA. Quantitative PCR was performed using the TB Green PCR Kit (Takara, Japan). The primer sequences were: RanBP3 forward 5′-GCCAGAAGCCCAAGGAG-3′ and reverse 5′-CAGCAGTGTCAGGGGATG-3′; Actin forward 5′-ACTTAGTTGCGTTACACCCTT-3′ and reverse 5′-TGTCACCTTCACCGTTCC-3′; P21 forward 5′-GACACCACTGGAGGGTGACT-3′ and reverse 5′-CAGGTCCACATGGTCTTCCT-3′.

### Immunofluorescence Assay

Cells were collected, washed and coated on the slides. The sildes were fixed with 4% paraformaldehyde for 0.5h, treated for 15min at 37°C using the 0.2% Triton X-100, blocked with goat serum at 4°C for 2h, and incubated with primary antibodies (Santa, USA) at 4°C overnight. On the next day, after removing the primary antibodies, cells on slides were washed 4 times using PBS, incubated with fluorescent-conjugated secondary antibodies (Introvigen, USA) at 37°C for 1h, then incubated with DAPI at 37°C for 15min in the dark. Finally, cells were sealed with glycerin and observed by the confocal microscope.

### Cell Cycle and Apoptosis Analysis

Propidium iodide (PI) staining was performed to examine the cell cycle. Briefly, 1×10^6^ cells were fixed with cold 75% ethanol for 1h, washed by PBS, re-suspended by 500μl PI/RNase solution, and incubated in darkness for 0.5h before detection of flow cytometry (FCM).

For apoptosis detection, the cell apoptosis analysis kit (Sungene Biotech, China) was performed with the instructions of manufacturer. In short, 1×10^5^ cells were collected, washed, suspended in 100μl buffer and incubated with 7-AAD and Annexin V-PE for 15 minutes in darkness, then detected by FCM within 1h.

### Cell Viability Assay

Cell viability was performed using the CCK-8 reagents (Life-iLab, China) at 24, 48, 72, and 96h. The infected cells were collected and adjusted to the concentration of 5×10^3^ per well. Cells were added with 10μl CCK-8 at the specified time and cultured at 37°C for 3h, then absorbance values were measured at 450nm. Each group has 3 duplicate wells.

### Cell Colony-Forming Assay

300 infected cells were suspended in a 750μl RPIM1640 medium containing 20% FBS and seeded in a well of 24-well plate, another 750μl semi-solid methylcellulose was supplemented in the well and stirred evenly with the medium. Cells were cultured for 7-10d at 37°C, then colonies were recorded and counted. Three multiple holes were set in each group.

### Murine Tumor Models

Infected K562 cells were collected, counted, washed by pre-cooled PBS and adjusted at the number of 2.5×10^7^/ml. Female NOD/SCID mice (weighing 18–20 g, 5 weeks old) were irradiated by 250cGy gamma rays before intravenous injection. 20 mice were randomly assigned into 4 groups and injected with 200μl cells respectively. After 10 days of inoculation, mice were injected intraperitoneally with IM (10mg/kg/mouse/day) for 14 days with PBS as control. The body weights, the number of white blood cells, and the percentage of human CD45 in mouse peripheral blood were recorded weekly. All procedures in the experiment were approved by the Ethics Committee of Chongqing Medical University.

### Statistical Analysis

Results of 3 independent tests were represented in the manner of mean ± SD. Statistical analysis was calculated by software SPSS 22.0 using t test (two groups) or One-way ANOVA (more than two groups) to calculate the main effect. When P < 0.001 (***), P < 0.01 (**), and p-values < 0.05 (*), statistical significance was determined.

## Results

### RanBP3 Is Highly Expressed in CML Cells

**Figure 1:**
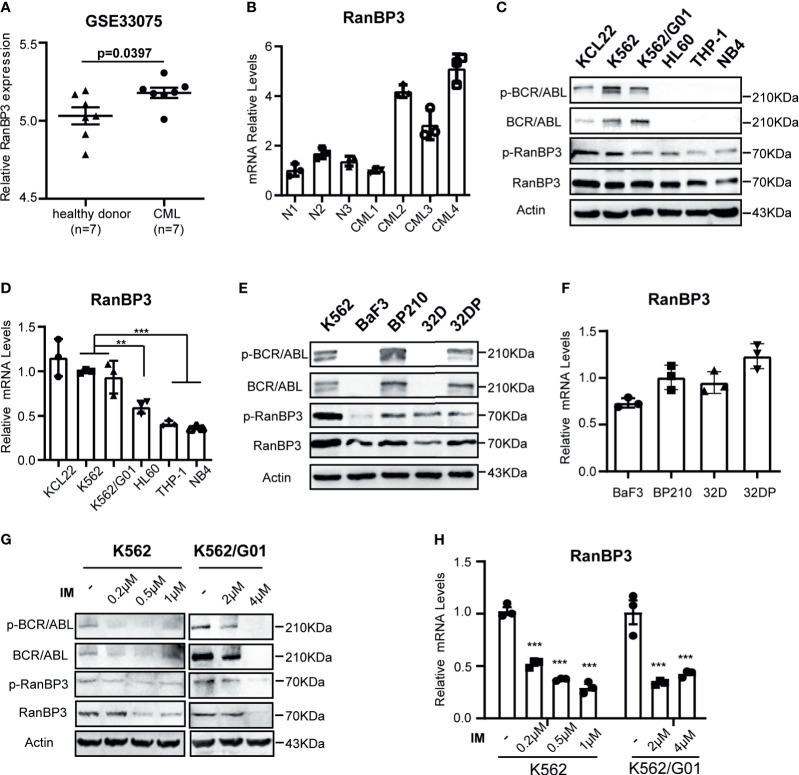
Expression of RanBP3 is up-regulated in CML. **(A)** The RanBP3 mRNA level was analyzed in database GSE33075, including 9 healthy individuals and 9 CML patients. After removing the outliers of each group, 7 samples of each group were used for expression analysis. **(B)** Bone marrow mononuclear cells separated from 3 normal controls and 4 CML patients were used to assess the mRNA levels of RanBP3. **(C)** The protein levels of RanBP3 were detected in BCR-ABL positive cells K562, K562/G01, KCL22 and BCR-ABL negative cells HL60, THP-1 and NB4. **(E)** The protein levels of RanBP3 in BCR-ABL transformed mouse cell lines and their corresponding control cells. **(D, F)** The mRNA levels of RanBP3 correspond to cells in **(C)** and **(E)**. **(G, H)** K562 and K562/G01 cells were treated with various concentrations of IM for 48 hours and the protein **(G)** and mRNA **(H)** levels of RanBP3 in these cells were detected. **P < 0.01, ***P < 0.001.

We analyzed RanBP3 expression levels in previously published gene expression data (GEO Series accession no. GSE33075), which contains bone marrow (BM) samples of 9 healthy donors and 9 CML patients (33). After removing the outliers, the expression of RanBP3 in each group including 7 samples was analyzed. As the **Figure 1A** showed, RanBP3 mRNA was higher in CML patients than that in healthy donors. Next, we collected BM samples of 3 healthy donors and 4 CML patients to measure the RanBP3 expression. The highly expressed mRNA levels of RanBP3 in CML patients were confirmed by Q-PCR (**Figure 1B**). To further assess the RanBP3 levels, leukemia cell lines K562, K562/G01, KCL22, HL60, THP1, and NB4 were examined. Consistently, the protein and mRNA detection showed that the RanBP3 levels in CML cells (BCR-ABL^+^) were much higher than that of other leukemia cells (BCR-ABL^-^) (**Figures 1C, D**). Moreover, using the BCR-ABL transformed 32DP and BP210 cells and untransformed 32D and BaF3 cells, we found that the protein levels of RanBP3 (**Figure 1E**) and the mRNA levels (**Figure 1F**) were higher in BCR-ABL^+^ 32DP and BP210 cells compared with the untransformed cells.

The fusion gene *bcr-abl* is considered as the primary cause of CML (5, 34), which has constitutive kinase activity in cytoplasm. We have confirmed the higher expression of RanBP3 in BCR-ABL^+^ cells compared with BCR-ABL^-^ cells (**Figures 1A–F**), so it is necessary to figure out the relation of RanBP3 and BCR-ABL activity in CML. We used the imatinib (IM) to inhibit the BCR-ABL activity of K562 and K562/G01 cells, as **Figure 1G** indicated, the protein levels of BCR-ABL, p-BCR-ABL, RanBP3 and p-RanBP3 were reduced in a dose-dependent way with the increased IM concentration. At the same time, Q-PCR showed the decreased RanBP3 mRNA after IM-treatment for 48h (**Figure 1H**), suggesting a possible correlated of RanBP3 expression and BCR-ABL activity. In summary, these findings indicate that RanBP3 is highly expressed in CML and downregulated after IM treatment, suggesting a possible role of RanBP3 in CML.

### RanBP3 Promotes the Proliferation of CML Cells

**Figure 2:**
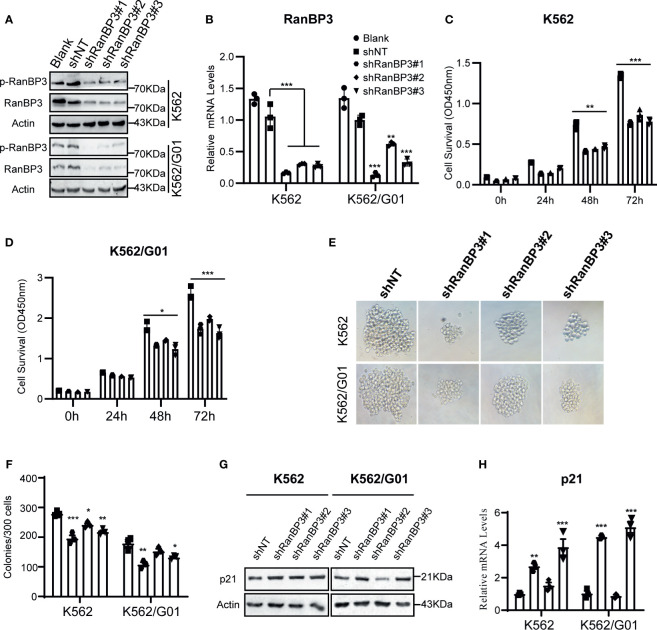
Effect of RanBP3 on the proliferation of CML cells. K562 and K562/G01 cells were infected with the lentivirus-carrying shRanBP3 and control shRNAs for 72h and collected for following assays. **(A, B)** RanBP3 levels in shRanBP3 and control cells were measured by western blots and qRT-PCR. **(C, D)** Cell viability was detected by CCK-8 at indicated time-points. The size **(E)** and number **(F)** of colonies were measured in lentivirus infected K562 or K562/G01. The protein **(G)** and mRNA **(H)** levels of p21 were evaluated. *P < 0.05, **P < 0.01, ***P < 0.001.

To investigate the function of RanBP3 in CML, we manipulated RanBP3 levels in K562 and K562/G01 cells using lentivirus-carrying shRNAs. The knockdown efficiency of RanBP3 was measured with western blot and Q-PCR, and shRanBP3#1 markedly inhibited RanBP3 expression (**Figures 2A, B**). Next, by counting the cell numbers of shRNAs-infected cells at 72h, we found that cell proliferation was obviously impaired (**Supplementary Figure 1A**). Meanwhile, the CCK-8 assay (**Figures 2C, D**) showed RanBP3 silencing significantly inhibited the viability of CML cells. In cell colony-forming assay, the sizes (**Figure 2E**) and numbers (**Figure 2F**) of colonies were suppressed in RanBP3-silenced cells. We also tested the cell cycle using PI staining and found RanBP3 knockdown slightly blocked cell cycle in the G1 phase (**Supplementary Figure 1B**). Western blot showed an up-regulated level of p21 (**Figures 2G, H**), which supported the increased G1 cell cycle fraction. These results demonstrate that RanBP3 can regulate CML cell proliferation.

### Silencing of RanBP3 Induces Cell Apoptosis in CML

**Figure 3:**
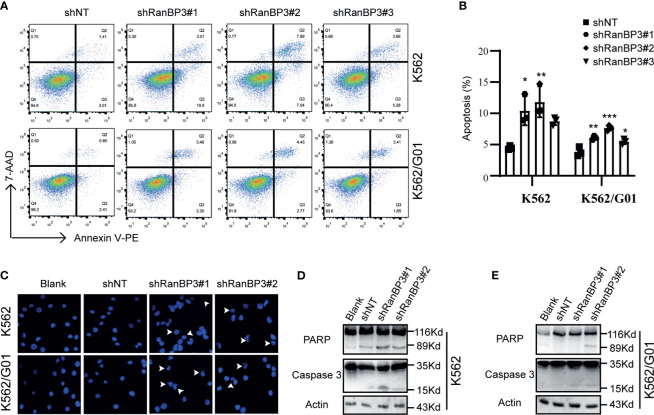
Effect of RanBP3 on the apoptosis of CML cells. **(A)** Infected cells were labeled with Annexin V-PE and 7-AAD and examined by FCM. **(B)** The apoptosis results of three times in **(A)** were recorded and plotted as a bar graph. **(C)** DAPI staining was used to assess morphologic changes in apoptosis. The prominent apoptotic morphologies were indicated using white arrows. **(D, E)** Cleaved PARP and Caspase-3 were evaluated by western blot in lentivirus infected cells. *P < 0.05, **P < 0.01, ***P < 0.001.

Meanwhile, we examined the role of RanBP3 silencing in the apoptosis of CML cells. Flow cytometry showed increased apoptosis in IM sensitive K562-shRanBP3 cells and IM resistant K562/G01-shRanBP3 cells compared with shNT-infected cells (**Figures 3A, B**), and results of the DAPI staining experiment were consistent with that of flow cytometry (**Figure 3C**). Western blots showed the enhanced expression of activated caspase-3 and cleaved PARP (**Figures 3D, E**), suggesting that apoptosis was induced through the caspase-3 dependent pathway. Together, our data showed that knockdown of RanBP3 induced apoptosis in CML cells.

### Silencing of RanBP3 Increases the Sensitivity of CML Cells to IM

**Figure 4:**
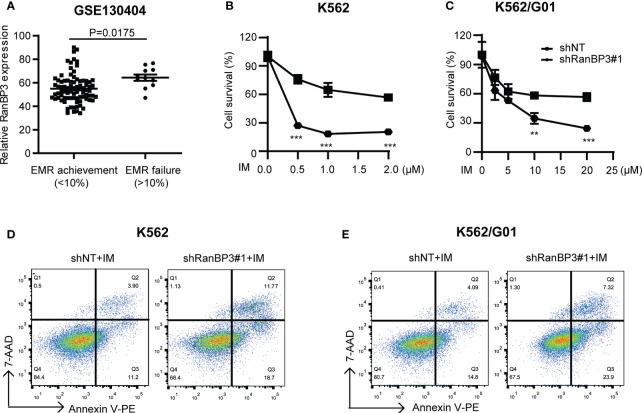
RanBP3 silencing increases the IM-induced apoptosis of CML cells. **(A)** The database GSE130404 contains 83 patients with early molecular response achieved (EMR achieved: BCR-ABL1 < 10% (IS) at 3 months) and 13 patients with early molecular response failure (EMR failure: BCR-ABL1 > 10% (IS) at 3 months). After excluding the outliers of each group, the P-value of RanBP3 expression of these two groups was calculated. **(B, C)** K562 (or K562/G01) cells and their derivatives were inoculated in 96-well culture plates (5000 cells/100μl per well), then treated with different concentrations of IM for 48h. The OD450nm value of each well was measured by CCK-8 and the cell survival rate was calculated. **(D, E)** Cells were infected with the lentivirus-carrying shRanBP3 and control shRNAs for 48h and followed by IM treatment for 48h (0.5μM IM for K562, 2μM IM for K562/G01). Apoptosis was examined by FCM. **P < 0.01, ***P < 0.001.

Considering the outstanding anti-tumor effect of silencing RanBP3 in both imatinib sensitive and resistant CML cells (**Figures 2**, **3**), it is necessary to investigate the relationship between RanBP3 and imatinib sensitivity in these cells. By analyzing the published database (GEO Series accession no. GSE130404), which contains 83 IM-treated patients with early molecular response achieved (EMR achieved: BCR-ABL < 10% (IS) at 3 months) and 13 IM-treated patients with EMR failure (BCR-ABL > 10% (IS) at 3 months) (35), we found that the expression of RanBP3 in EMR failure patients was much higher compared with the EMR achieved patients (**Figure 4A**), suggesting a possible link between RanBP3 and IM resistant. Then we performed CCK-8 assay to measure the cell viability in shRanBP3 or shNT infected CML cells after IM treatment for 24h. Data showed that RanBP3 knockdown significantly decreased cell viability in IM-treated K562 (**Figure 4B**) and K562/G01 (**Figure 4C**). Flow cytometry (**Figures 4D, E**) showed that RanBP3 knockdown remarkably increased the apoptosis in IM-treated K562 and K562/G01 cells. Overall, our results suggest that RanBP3 can regulate the sensitivity of IM in CML cells.

### Silencing of RanBP3 Enhances the Cells Sensitivity of IM in CML Mouse Model

**Figure 5:**
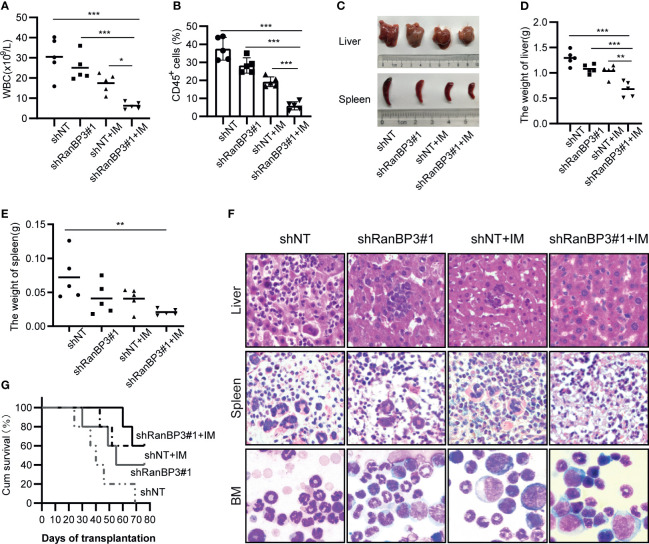
RanBP3 silencing increases IM sensitivity *in vivo*. K562-shRanBP3 or K562-shNT cells were injected into the tail veins of NOD/SCID mice. 10 days after injection, 10mg/kg IM or control PBS was injected intraperitoneally for 14 days. **(A)** The total WBC counts in mice were determined. **(B)** The percentage of human CD45^+^ cells in BM cells of NOD/SCID mice was measured by FCM. **(C)** The images of livers and spleens were shown. The weights of livers **(D)** and spleens **(E)** were quantified. **(F)** Murine liver and spleen infiltration in 4 groups were analyzed by H&E, and bone marrow cells were checked with Wright’s stain. **(G)** Survival curves were drawn by GraphPad 8.0 and analyzed by Kaplan–Meier methods. *p< 0.05, **p< 0.01 and ***< 0.001.

Based on our data from cell experiments, to further explore the effect of RanBP3 on the pathogenicity of CML cells and chemo-sensitivity of IM *in vivo*, we constructed a CML mouse model. K562-shNT and K562-shRanBP3 cells were injected i.v. into NOD/SCID mice, respectively. 10 days after the injection, 10mg/kg IM started in the treated group (once a day, 14 days, i.p.), while the equal volume of PBS was injected as control. 4 weeks after cell injection, mice in the K562-shNT group first showed symptoms of mental fatigue, fluffy hair, lameness, weight loss and leukocytosis, which were consistent with the published symptoms of the murine CML-xenograft model (36). As **Figure 5A** showed, the leukocytes in the K562-shNT group were much lower than these in the K562-shRanBP3 group, which was more significant in these two groups with IM-treatment. To figure out whether the increased white blood cells (WBC) were caused by human K562 engraftment or not, leukocytes from murine peripheral blood (PB) were incubated with human-CD45 antibody, flow cytometry was performed to evaluate the ratio of human cells (37). As **Figure 5B** indicated, the proportion of human-CD45 positive K562 cells in murine PB were much lower in the K562-shRanBP3 group than these in the K562-shNT group, especially in the IM-treated group. These results demonstrated that RanBP3 knockdown suppressed K562 cells proliferation *in vivo*, and combining with IM could enhance this effect.

Histopathological changes such as hepatosplenomegaly and infiltration of K562-like cells in the liver, spleen, and bone marrow (BM) were also characteristic of CML mice (36, 38). Indeed, we observed severe hepatosplenomegaly in K562-shNT mice compared with K562-shRanBP3 mice whether IM was added or not (**Figure 5C**), and the weights of mouse livers (**Figure 5D**) and spleens (**Figure 5E**) were consistent with their morphology. To examine the infiltration of leukemic cells in the mouse model, hematoxylin/eosin (HE) staining was performed in murine liver and spleen, which showed an alleviation of leukemic infiltration in the K562-shRanBP3 xenograft model, especially in IM-treated group. And Wright’s staining assay of mice BM cells showed that RanBP3 knockdown alleviated the increased ratio of myeloid: erythroid caused by K562 cell engraftment, and this ability was enhanced when combined with IM therapy (**Figure 5F**). In addition, the BCR-ABL expression in mouse spleen, liver and BM was detected by immunofluorescent and the result was consistent as above (**Supplementary Figure 2**). Kaplan-Meier survival analysis indicated that the mice of the IM-treated K562-shRanBP3 group had the longest survival time among the 4 groups (**Figure 5G**). In summary, our results showed that inhibition of RanBP3 may delay the malignant progression onset of CML *in vivo*, and enhance the chemosensitivity of CML cells to IM in mice.

### RanBP3 Mediates Nuclear Transport of SMAD2/3 and ERK1/2

**Figure 6:**
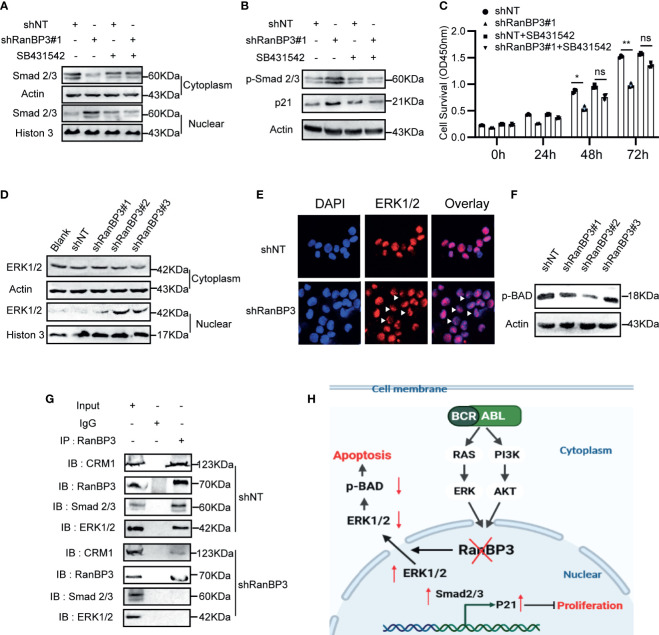
RanBP3 regulates cell proliferation and apoptosis by mediating the nuclear transport of SMAD2/3 and ERK1/2. K562-shRanBP3 and K562-shNT were treated with DMSO or 10μM SB432542 for 8h. **(A)** The nuclear and cytosol proteins were extracted and used for the detection of SMAD2/3 by western blot. **(B)** Total proteins were obtained for the detection of p-SMAD2/3 and p21 levels. **(C)** Cell survival was determined by CCK-8 at the indicated time. The cytoplasmic and nuclear distribution of ERK1/2 was analyzed in K562-ShRanBP3, K562-shNT and K562 by western blot **(D)** and immunofluorescent **(E)**. **(F)** The expression of p(ser112)-BAD was determined in K562-ShRanBP3 and K562-shNT cells. **(G)** CO-IP assay was performed using the antibody anti-RanBP3 to examine the interaction of proteins in K562-ShRanBP3 and K562-shNT cells, and the RanBP3-bound proteins were determined by anti-ERK1/2, anti-CRM1, and anti-SMAD2/3 antibody. An anti-Rabbit IgG antibody was used as the negative control. **(H)** The working model for RanBP3 silencing-mediated proliferation and apoptosis in CML. RanBP3 is a downstream molecule of BCR-ABL and regulates SMAD2/3 and ERK1/2 nuclear export. After the knockdown of RanBP3, nuclear SMAD2/3 increased followed by the enhanced levels of p21, which inhibited cell proliferation, and the decreased cytoplasmic ERK1/2 levels relieved the antiapoptotic effect of p-BAD in CML. *p < 0.05, **p < 0.01 and ns means no significant.

Previous studies (27, 30) have shown that RanBP3 is involved in nuclear transport of some proteins in the CRM1-independent manner, such as β-catenin, SMAD2/3, and ERK1/2. Based on our data from the CML cells and mouse models, we speculated that RanBP3 may regulate the proliferation and apoptosis of CML by alterations of certain proteins localization in cells. We found that the levels of p-SMAD2/3, p21, and nuclear SMD2/3 increased after RanBP3 knockdown (**Figures 6A, B** and **Supplementary Figure 3A**), indicating that the transforming growth factor-β (TGF-β) signal pathway may be activated. Indeed, the up-regulation of p-SMAD2/3 and p21 in RanBP3-silenced K562 cells could be neutralized by TGF-β inhibitor (SB431542) (**Figure 6B**). Furthermore, the CCK-8 assay (**Figure 6C**) showed that SB431542 treatment relieved the anti-proliferative effect of RanBP3 knockdown. Herein, these results suggest that RanBP3 may promote cell proliferation in CML by regulating the TGF-β signaling.

BAD is a pro-apoptotic protein in the cytoplasm. The MAPK signaling may overcome the pro-apoptotic activity of BAD through phosphorylating BAD at ser112 (39). In our study, the increased nuclear ERK1/2 (**Figures 6D, E**) and decreased expression of p(ser112)-BAD were observed in K562-shRanBP3 cells (**Figure 6F**), which was associated with a decreased level of cytoplasmic ERK1/2. And the combination of RanBP3 and ERK1/2 was substantiated by the co-immunoprecipitation assay (**Figure 6G**). To evaluate the further relationship between RanBP3 and ERK1/2 related pathway, U0126, an inhibitor of MAPK pathway, was used to inhibit the phosphorylation of ERK without affecting the expression of ERK. Notably, the levels of RanBP3, p-RanBP3, and p(ser112)-BAD were down-regulated in CML cells after treating with U0126 for 6h (**Supplementary Figure 3B**). These findings indicate that RanBP3 regulates cell apoptosis in CML by transporting ERK1/2 out of the nucleus.

## Discussion

Abnormal nuclear-cytoplasmic localization of the protein is an important cause of cancer occurrence and drug resistant (20). CRM1 is the most common transport receptor protein mediating the export of over 241 proteins, which makes it a putative good target in various tumors with abnormal protein localization (40, 41). However, off-target toxicity of CRM1 inhibitors, such as serious gastrointestinal adverse events and thrombocytopenia, has suppressed their further research (42). Thus, the exploration of safer inhibitors in nuclear exporting is needed. RanBP3 is best known as a cofactor in CRM1-mediated nuclear export (43), but it is also reported to export β-catenin and SMAD2/3 in a CRM1-independent manner (44, 45). Besides, RanBP3 is a crucial downstream molecule of MAPK-ERK and PI3K-AKT signal pathways, which links the oncogenic signals to nuclear transport (26). Recently, Gaurav et al. showed that RanBP3 regulated cell proliferation through selective control of nuclear export in melanoma (27). And other studies have begun to concern about the potential role of RanBP3 in tumor progression (28–30). These findings suggest that RanBP3 may play an irreplaceable role in tumors by regulating the subcellular localization of proteins. However, the possible relationship between RanBP3 and CML has never been investigated.

Herein, we first detected the level of RanBP3 in CML. Using published GEO data (GSE33075) and the collected patients samples, we found the over-expression of RanBP3 in CML patients. The protein and mRNA levels of RanBP3 in CML cell lines (BCR-ABL^+^) were also higher compared with other control cells (BCR-ABL^-^) (**Figures 1C–F**). Next, we tested the levels of RanBP3 in IM treated cells and found that the downregulation of RanBP3 was consistent with the decrease of BCR-ABL, suggesting a possible correlation between BCR-ABL and RanBP3. Indeed, it is well known that the kinase activity of BCR-ABL in CML can activate the downstream MAPK and AKT pathways (7), and RanBP3 has been reported to be the common downstream of the two pathways (26). But further study is needed to demonstrate the direct relationship between BCR-ABL and RanBP3.

To figure out the role of RanBP3 in CML cells, we used shRNA lentivirus to knockdown RanBP3 in K562 and K562/G01 cells and found that silencing of RanBP3 could significantly inhibit proliferation and induce apoptosis. At the same time, cleaved caspase3 and PARP increased, suggesting apoptosis was caspase3-related in RanBP3-silenced CML cells. These findings were consistent with the previous study (27). Furthermore, we used GEO data to show the possible role of RanBP3 in patients with IM treatment and did substantiate that down-regulation of RanBP3 improved apoptosis of IM-treated CML cells.

Finally, we examined the nuclear export mediated by RanBP3 to understand the mechanism of RanBP3 in cell proliferation and apoptosis. SMAD2/3 is the key transducer in the signaling pathway of TGF-β, when it was activated and imported into the nucleus, the downstream transcription, such as p21, p15, c-myc, cyclin D1, and so on, were regulated. In this process, the TGF-β pathway can regulate many biological processes, such as cell and tissue growth, differentiation, development, embryonic development, apoptosis, and so on (46–49). Here, we reported that RanBP3 silencing increased nuclear SMAD2/3, up-regulated the level of p21, and then inhibited proliferation of CML cells. And SB43154, a kind of TGF-β inhibitor, was used to rescue these anti-proliferation effects. These results indicated that RanBP3 mediates CML proliferation by negatively regulating the tumor growth inhibitor axis TGF-β-SMAD2/3-p21.

As Englemeier et al. reported, CRM1 regulates the cargos nuclear export by recognizing and combining with its nuclear export sequence (NES), and RanBP3 is essential in CRM1-mediated export when the cargo contains a weak NES (50). Gaurav et al. have confirmed that extracellular signal-regulated kinase 1/2 (ERK1/2) was a cargo harboring the weak NES, thus requiring RanBP3 to participate in its nuclear export (27). In CML, with the stimulation of BCR-ABL kinase activity, ERK1/2 is over-activated in the cytoplasm and exerts the oncogenic potential by enhancing the activities of anti-apoptosis proteins, such as Bcl-2, p-BAD, Bcl-XL, and Mcl-1, and inhibiting pro-apoptotic proteins, such as Bad and Bim (39, 51). Here, we reported the reduction of p(ser112)-BAD and cytoplasm ERK1/2 in RanBP3-silenced CML cells, suggesting that the RanBP3 silencing induced apoptosis may be related to MAPK signaling. The activation of ERK1/2 after long-term imatinib treatment is a kind of CML resistance mechanisms, therefore, combination of ERK inhibitors and imatinib has been used in IM-resistant CML (52–54). Recently, ERK1/2 cellular localization was regarded as a considerable pathogenic factor, and the accumulation of nuclear ERK signaling or the lacking of tumor-related cytoplasmic ERK may be the mechanism of regulating cell progression (55, 56). However, how to achieve balanced subcellular distribution of ERK needs more research. Our study on the role of RanBP3 in regulating ERK1/2 localization in CML has laid a foundation for addressing the above issues.

In conclusion, we proved that RanBP3 was important in regulating proliferation and apoptosis in CML, and the potential mechanism was changing the distribution of cancer-related proteins between the cytoplasm and nucleus (**Figure 6H**). As the mislocalization of cancer-related proteins and tumor suppressor molecules may be the indispensable cause of tumorigenesis, further study will be performed to figure out the possible effects of RanBP3-medidated nuclear export in other cancers. Collectively, our data showed for the first time that RanBP3 was a potential target for CML, and played a crucial role in regulating proliferation, apoptosis, and the IM chemosensitivity of CML cells by mediating the nuclear export of SMAD2/3 and ERK1/2, suggesting that targeting RanBP3 alone or combined with IM can improve the clinical response of refractory and resistant CML patients. In addition, this study also provides a reference for functional study of RanBP3 in other tumors.

## Data Availability Statement

The original contributions presented in the study are included in the article/**Supplementary Material**. Further inquiries can be directed to the corresponding author.

## Ethics Statement

The studies involving human participants were reviewed and approved by The Ethics Committee of Chongqing Medical University. The patients/participants provided their written informed consent to participate in this study. The animal study was reviewed and approved by The Ethics Committee and Animal Care Institution of Chongqing Medical University.

## Author Contributions

QL and ZH discussed and designed the experiments. QL, ZH, and YP refined the experimental scheme and conducted the experiments. QL drew all figures and wrote the draft of manuscript. XW and TW provided patients samples. GJ and KM interpreted and analyzed the data. WF revised this manuscript. All authors contributed to the article and approved the submitted version.

## Funding

This work was funded by the National Science Foundation of China (No. 81572060).

## Conflict of Interest

The authors declare that the research was conducted in the absence of any commercial or financial relationships that could be construed as a potential conflict of interest.

## Publisher’s Note

All claims expressed in this article are solely those of the authors and do not necessarily represent those of their affiliated organizations, or those of the publisher, the editors and the reviewers. Any product that may be evaluated in this article, or claim that may be made by its manufacturer, is not guaranteed or endorsed by the publisher.
