# Supplementary material for: RanBP3 Regulates Proliferation, Apoptosis and Chemosensitivity of Chronic Myeloid Leukemia Cells via Mediating SMAD2/3 and ERK1/2 Nuclear Transport
Source: Front Oncol. 2021 Aug 24;11:698410. doi: 10.3389/fonc.2021.698410 (PMC8421687; doi:10.3389/fonc.2021.698410)
Supplement: Supplementary file 1 [file DataSheet_1.docx]

Supplementary Material

# Supplementary Figures and Tables

## Supplementary Figures

##
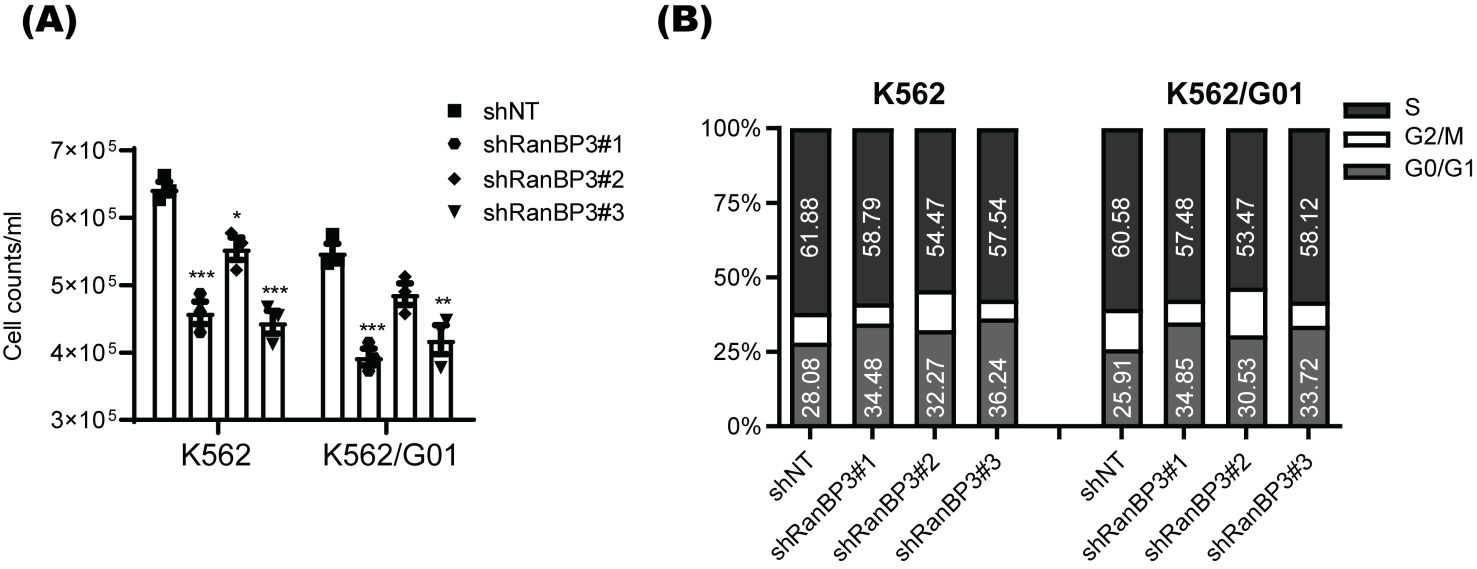


**Supplementary Figure 1.**

**RanBP3 regulates proliferation of CML cells.** K562 and K562/G01 cells were infected with the lentivirus-carrying shRanBP3 and control shRNAs for 72h and collected for following assays. (A) The numbers of K562 and K562/G01 cells were counted. (B) Cell cycle was determined by PI staining.

**
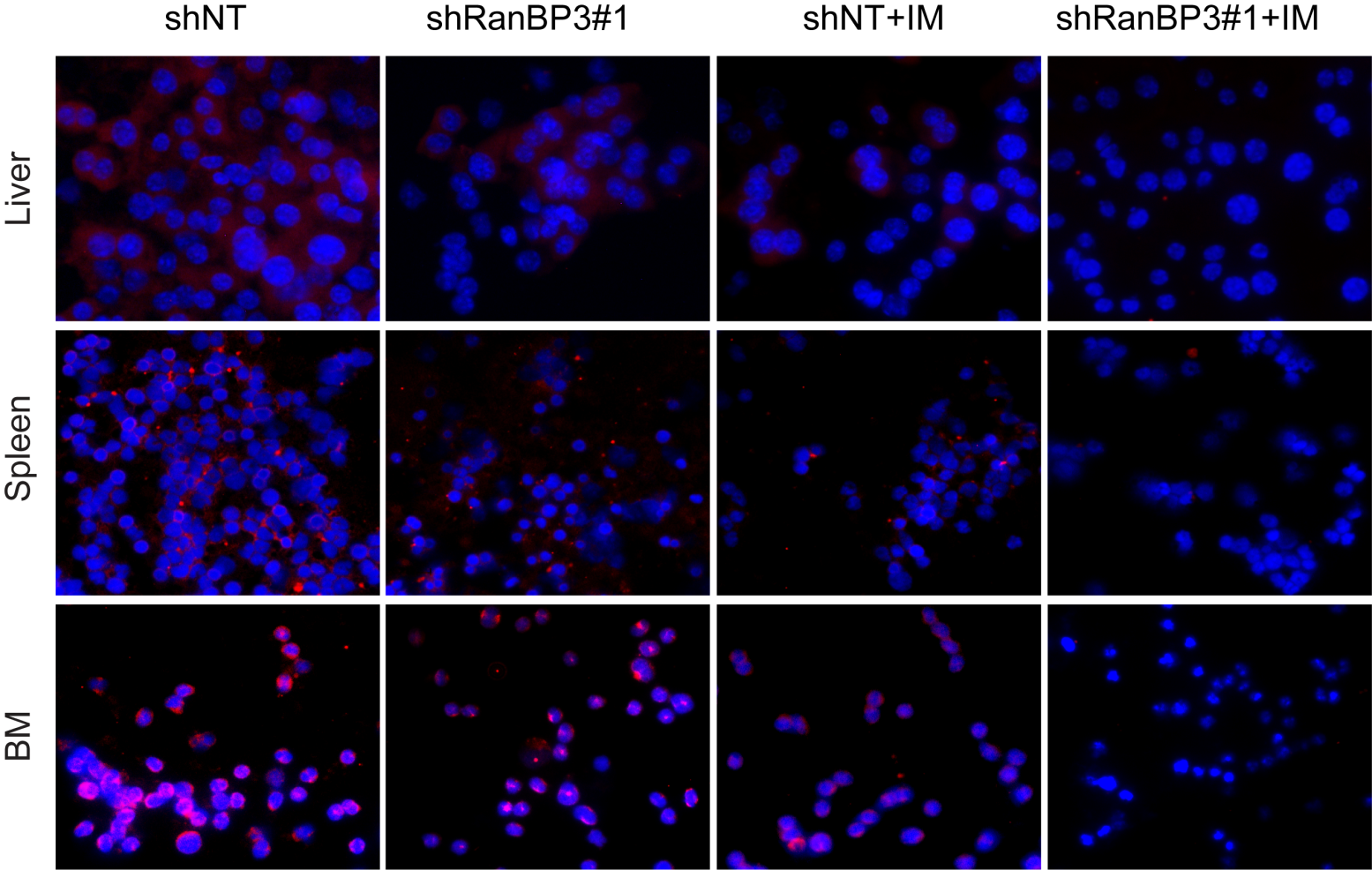
**

**Supplementary Figure 2.**

**RanBP3 silencing combined with IM treatment inhibited the leukemic infiltration in vivo.** Expression of BCR/ABL in murine liver, spleen and bone marrow cells were detected by immunofluorescent.

**
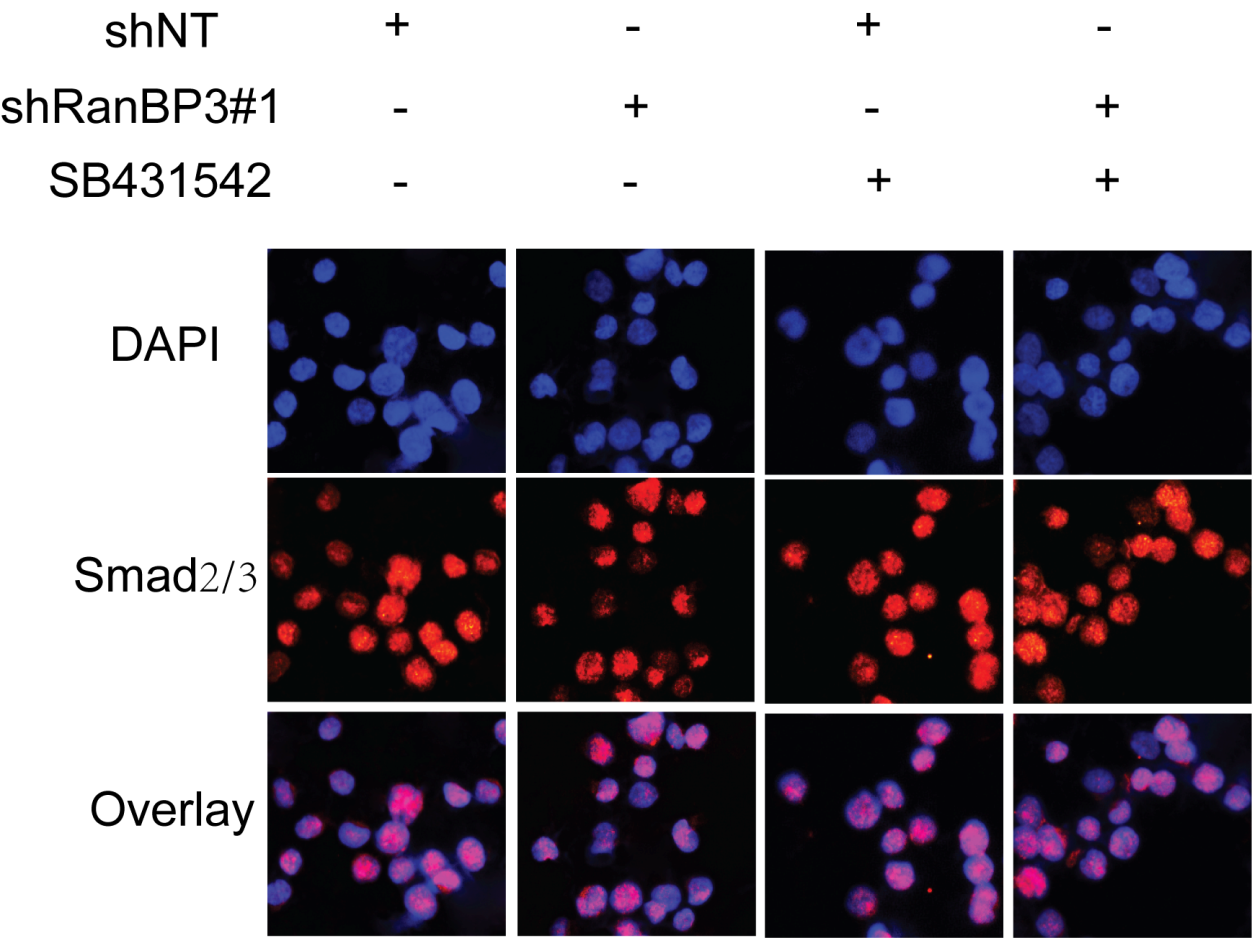
**

**Supplementary Figure 3.**

**RanBP3 regulates Smad2/3 nuclear export.** K562 cells were infected with the lentivirus for 72h followed by immunofluorescence staining with DAPI (top panel) and Smad2/3 (middle panel). The overlay of DAPI and Smad2/3 was shown at the bottom.

**
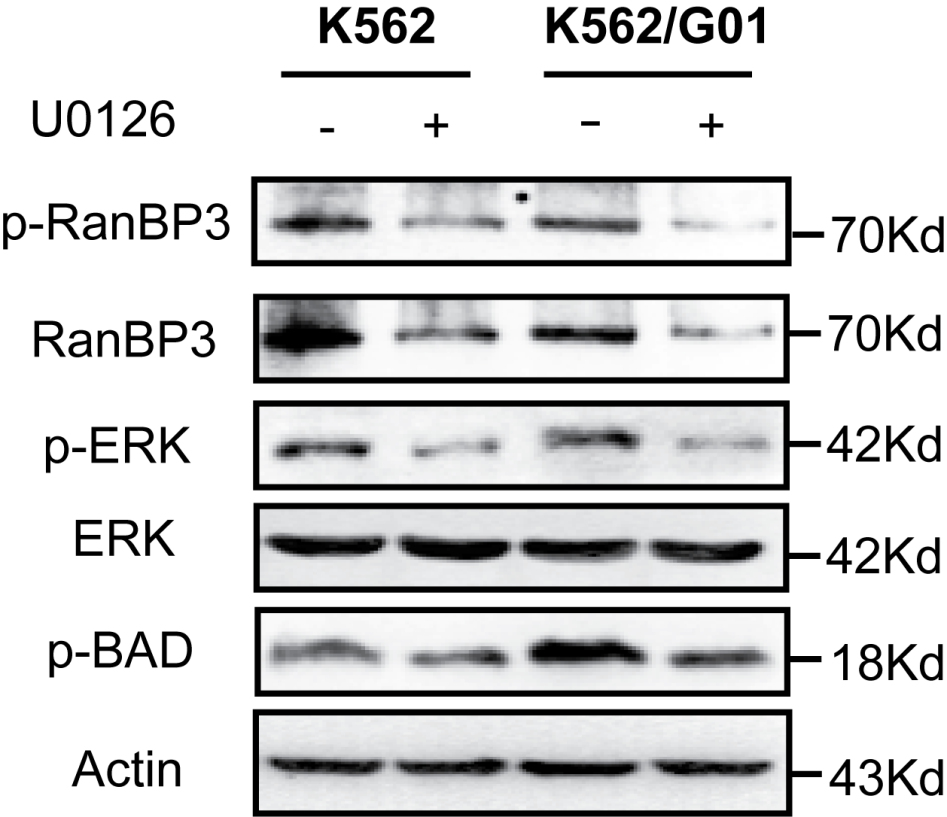
**

**Supplementary Figure 4.**

**The correlation of RanBP3 and MAPK signaling in CML.** K562 and K562/G01 cells were treated with MEK inhibitor, U0126 (10μM) or DMSO for 6 hours, western blot was performed to measure the expression of RanBP3, p-RanBP3, p-ERK, ERK and p-BAD.

## Supplementary Tables

**Supplementary Table 1. Patients information.**

| **Samples** | **Diagnosis** | **Stage of disease** | **Gender/Age** | **Karyotype** | **BCR-ABL1 copy number/ABL1 copy number*100% (IS)** | **WBC**  **(×10^9^ /L)** |
| --- | --- | --- | --- | --- | --- | --- |
| CML1 | Primary | CP | Female/66 | t (9;22) | 26.175% | 19.05 |
| CML2 | Relapse | AP | Male/45 | t (9;22) | 63.350% | 149.52 |
| CML3 | Primary | CP | Male/49 | t (9;22) | 79.910% | 70.25 |
| CML4 | Relapse | CP | Female/41 | t (9;22) | 63.350% | 208.16 |
| Normal1 | Anemia |  | Male/46 | normal |  | 3.71 |
| Normal2 | Anemia |  | Male/29 | normal |  | 5.23 |
| Normal3 | Anemia |  | Female/25 | normal |  | 6.27 |
