# Supplementary material for: RanBP3 Regulates Proliferation, Apoptosis and Chemosensitivity of Chronic Myeloid Leukemia Cells via Mediating SMAD2/3 and ERK1/2 Nuclear Transport
Source: Front Oncol. 2021 Aug 24;11:698410. doi: 10.3389/fonc.2021.698410 (PMC8421687; doi:10.3389/fonc.2021.698410)
Supplement: Supplementary file 2 [file DataSheet_2.zip › Figure 3 original data/3A.pdf]

# SHNT-K562

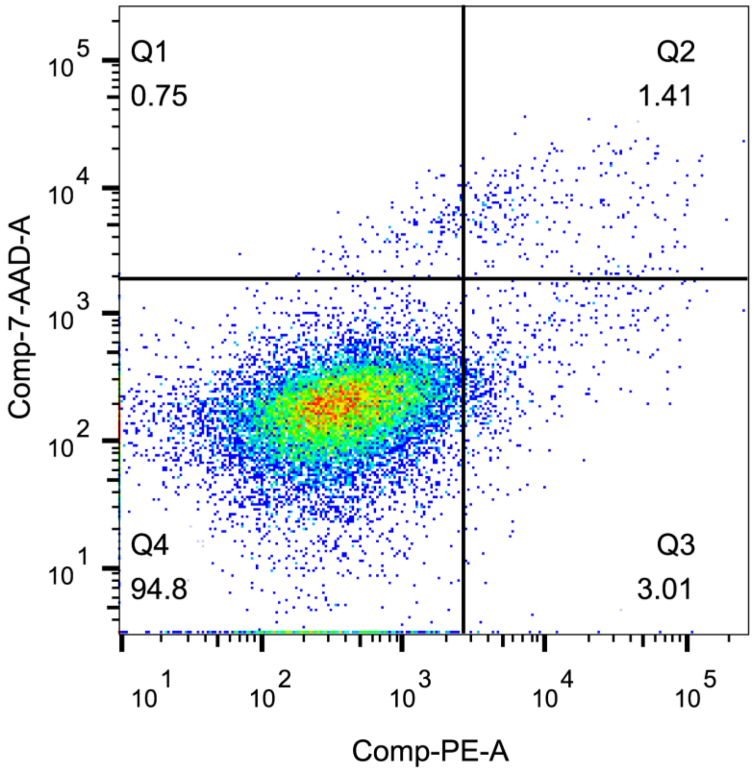


180716_Tube_001_017.fcs
Comp-GFP-A, SSC-A 子细胞群
18440

# SH1-K562

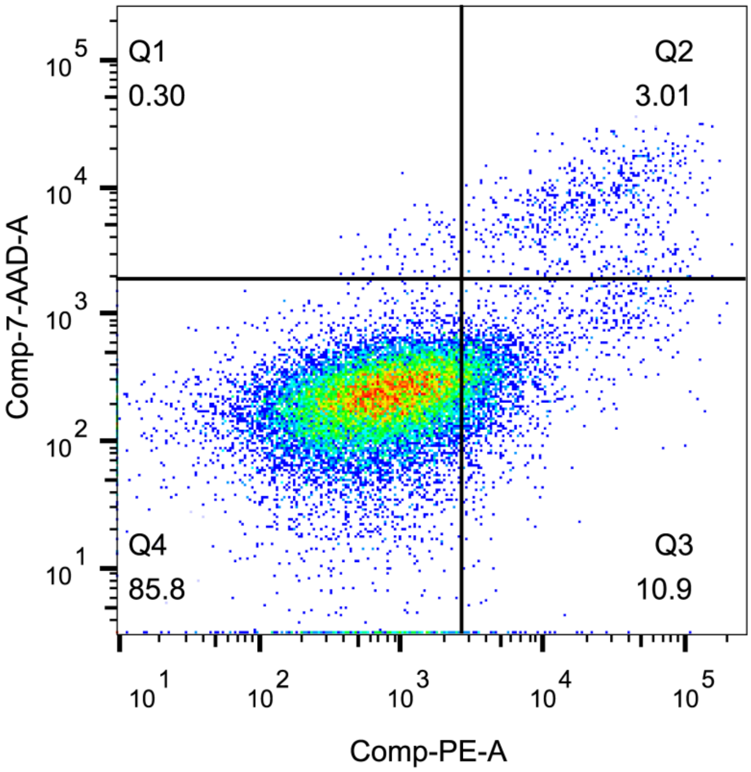


180716_Tube_002_018.fcs
Comp-GFP-A, SSC-A 子细胞群
21755

# SH2-K562

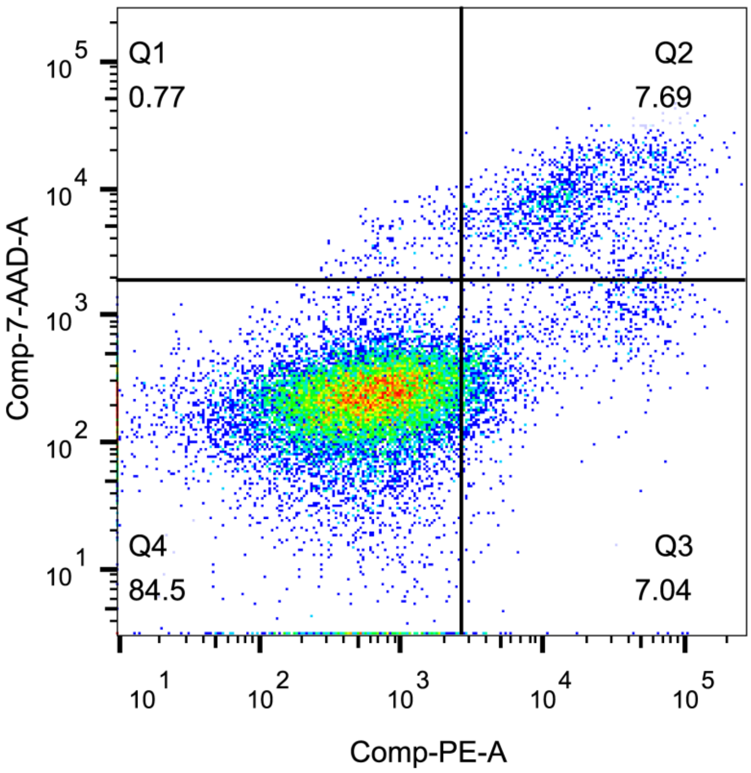


180716_Tube_003_019.fcs
Comp-GFP-A, SSC-A 子细胞群
19388

## SH3-K562

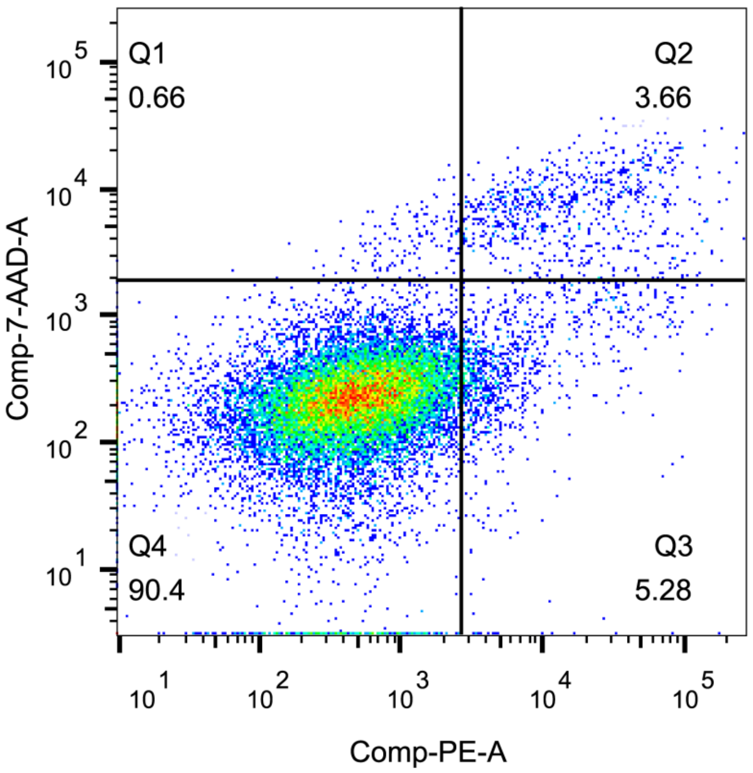


180716_Tube_004_020.fcs
Comp-GFP-A, SSC-A 子细胞群
21323

# SHNT-K562/G01

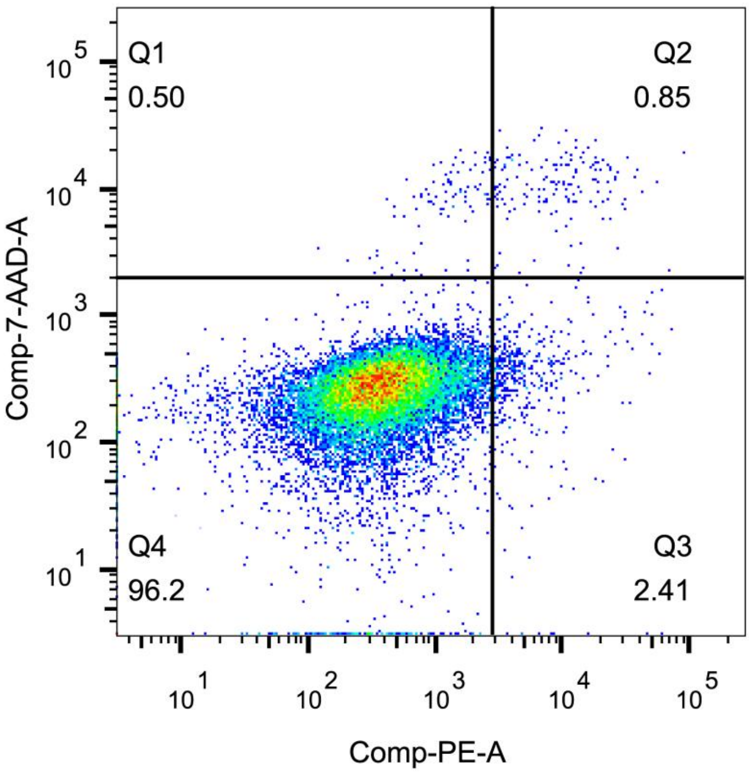


180626_Tube_001_009.fcs
Comp-GFP-A, SSC-A 子细胞群
17852

## SH1-K562/G01

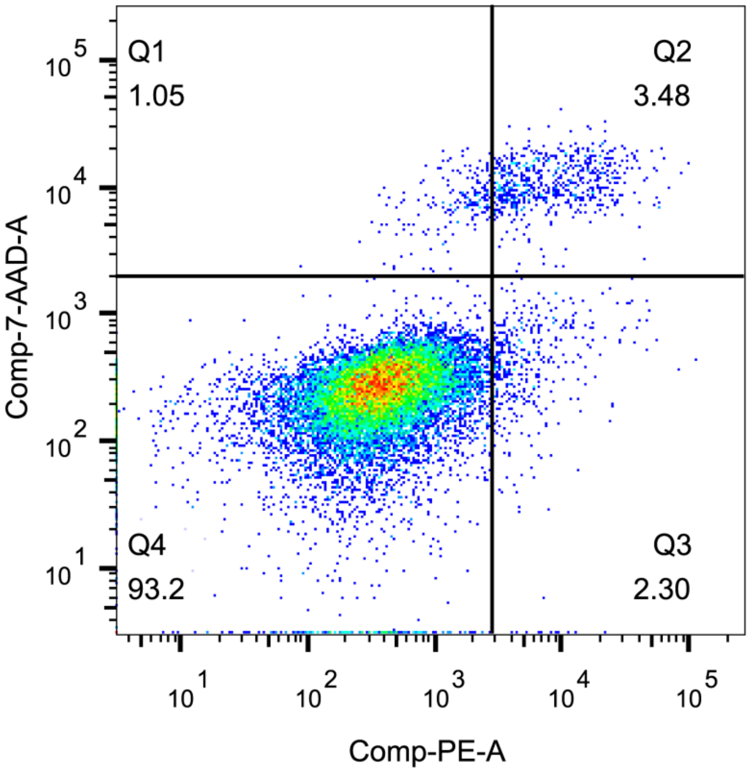


180626_Tube_002_010.fcs
Comp-GFP-A, SSC-A 子细胞群
18056

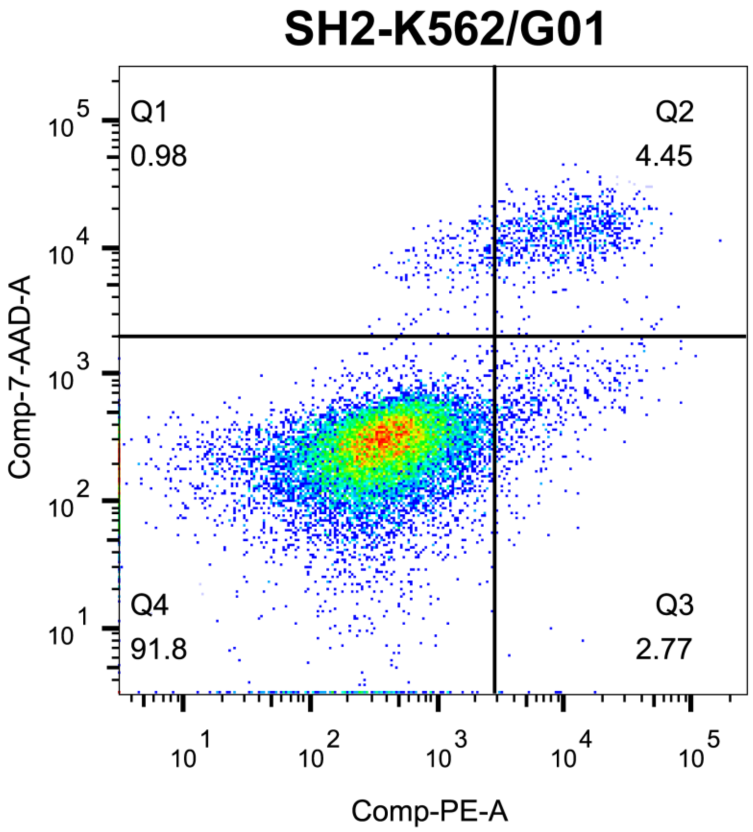


180626_Tube_003_011.fcs
Comp-GFP-A, SSC-A 子细胞群
18695

# SH3-K562/G01

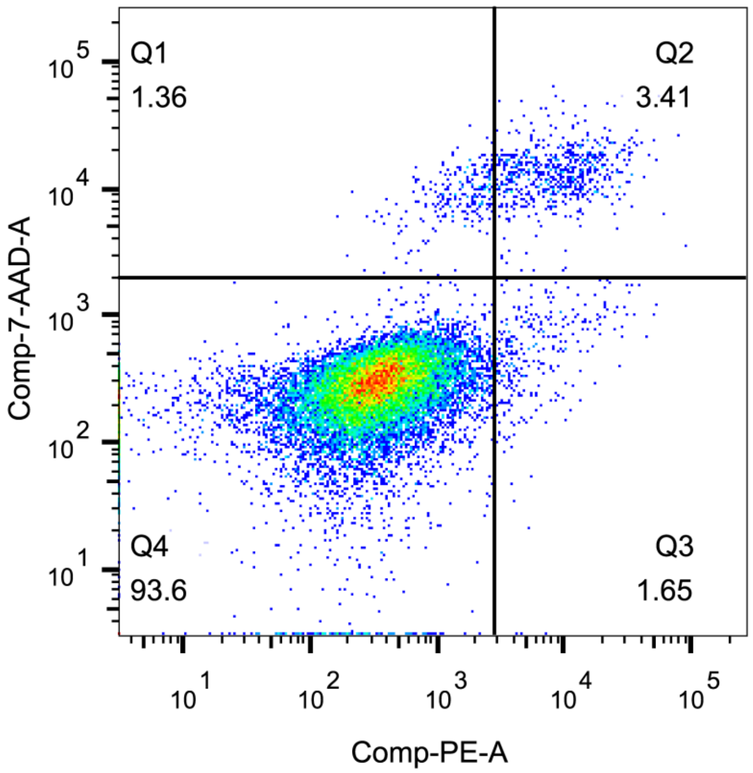


180626_Tube_004_012.fcs
Comp-GFP-A, SSC-A 子细胞群
18472
