# Supplementary material for: RanBP3 Regulates Proliferation, Apoptosis and Chemosensitivity of Chronic Myeloid Leukemia Cells via Mediating SMAD2/3 and ERK1/2 Nuclear Transport
Source: Front Oncol. 2021 Aug 24;11:698410. doi: 10.3389/fonc.2021.698410 (PMC8421687; doi:10.3389/fonc.2021.698410)
Supplement: Supplementary file 2 [file DataSheet_2.zip › Figure 3 original data/3D E.pptx]

## Slide 1
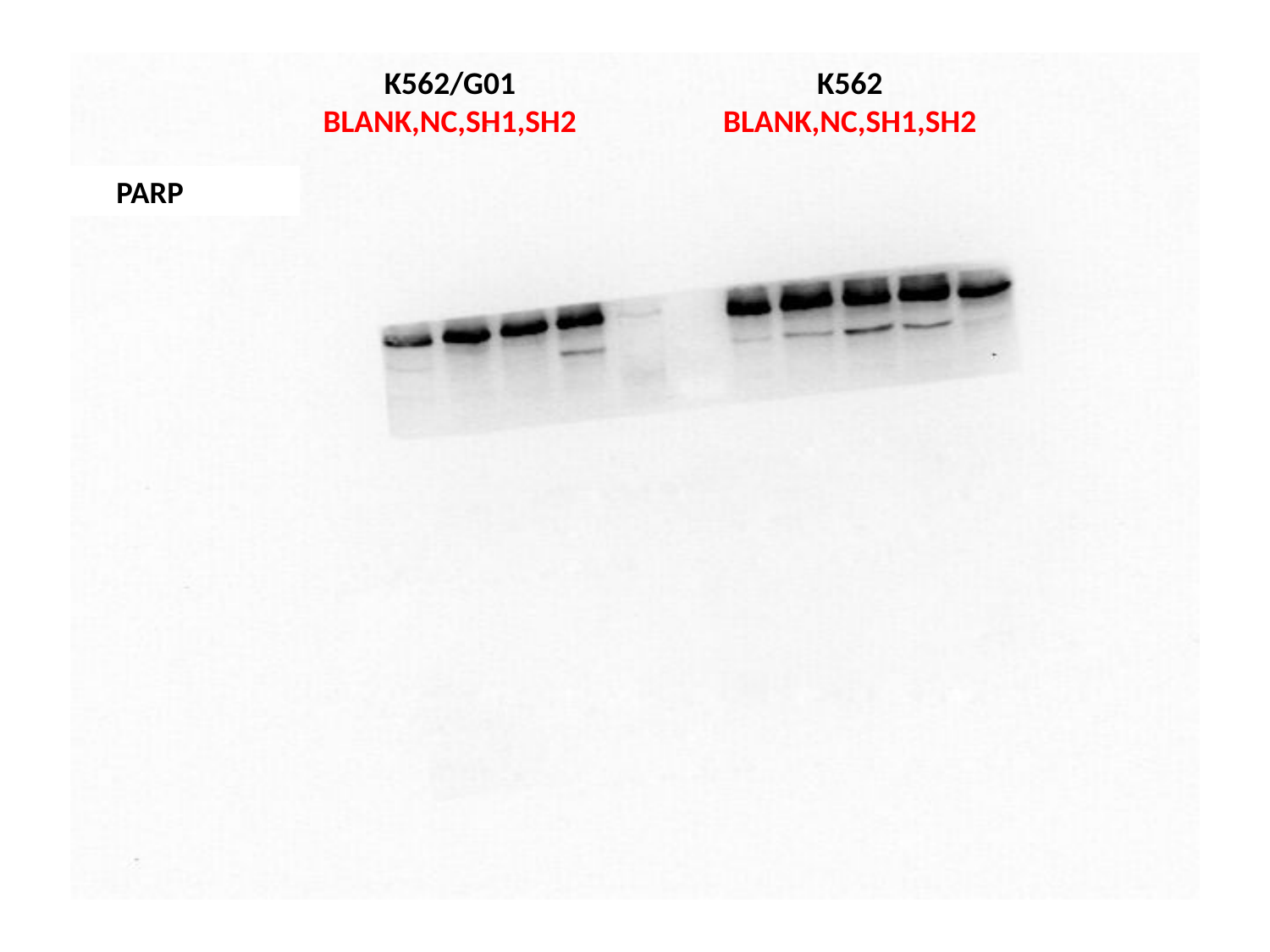

K562/G01
BLANK,NC,SH1,SH2
K562
BLANK,NC,SH1,SH2
PARP

## Slide 2
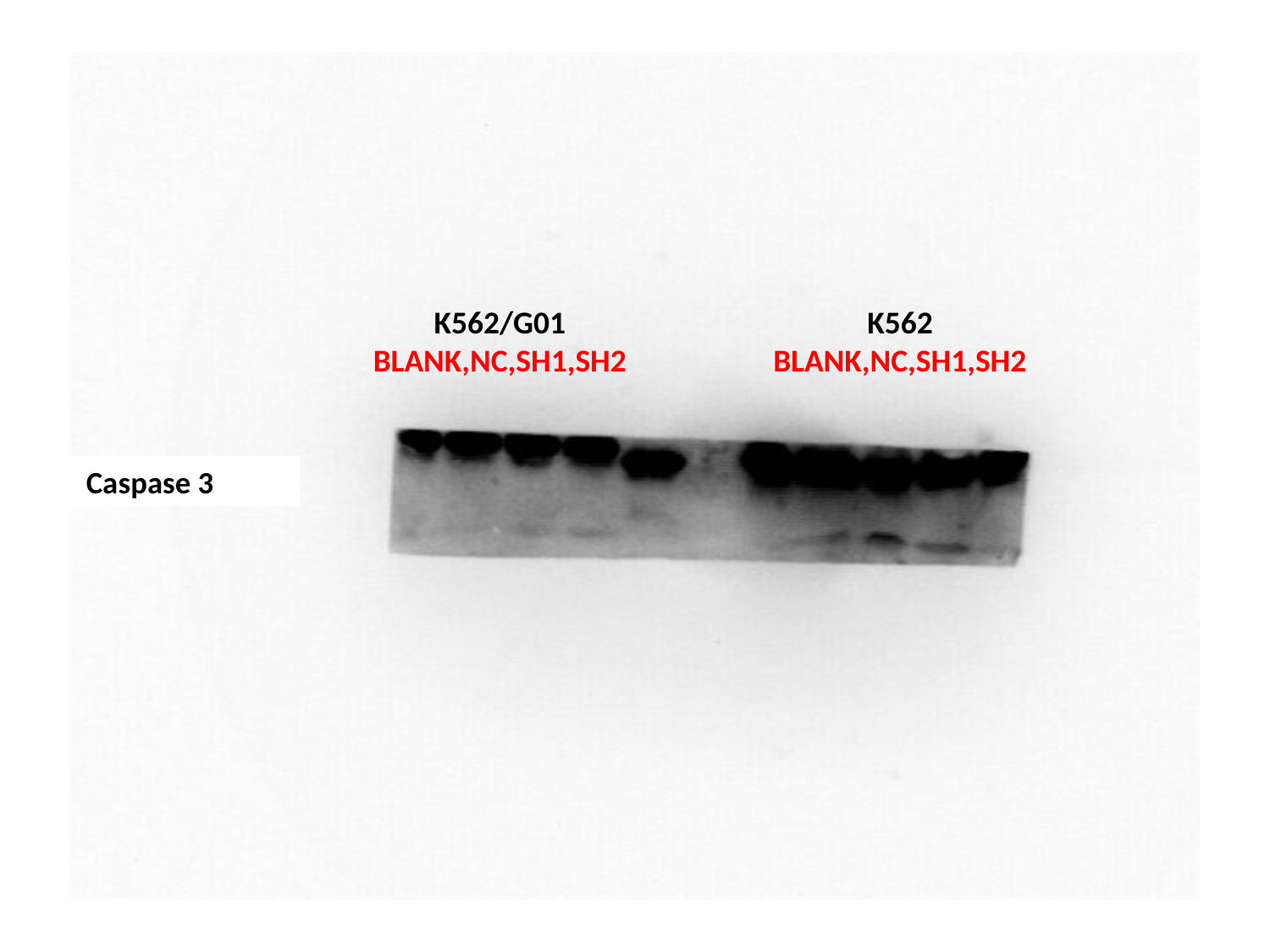

K562/G01
BLANK,NC,SH1,SH2
K562
BLANK,NC,SH1,SH2
Caspase 3

## Slide 3
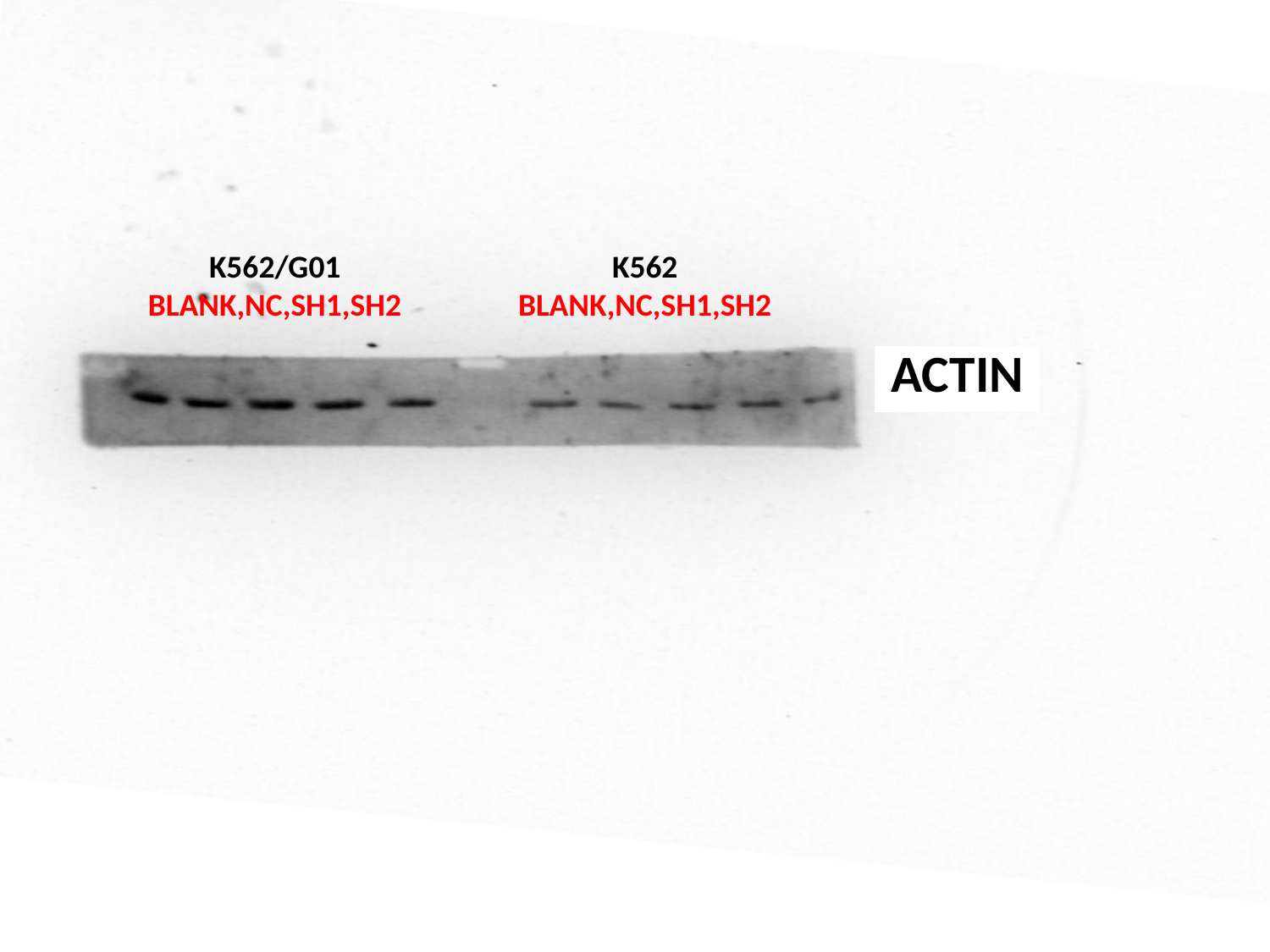

K562/G01
BLANK,NC,SH1,SH2
K562
BLANK,NC,SH1,SH2
ACTIN
