# Supplementary material for: RanBP3 Regulates Proliferation, Apoptosis and Chemosensitivity of Chronic Myeloid Leukemia Cells via Mediating SMAD2/3 and ERK1/2 Nuclear Transport
Source: Front Oncol. 2021 Aug 24;11:698410. doi: 10.3389/fonc.2021.698410 (PMC8421687; doi:10.3389/fonc.2021.698410)
Supplement: Supplementary file 2 [file DataSheet_2.zip › Figure 1 original data/1C.pptx]

## Slide 1
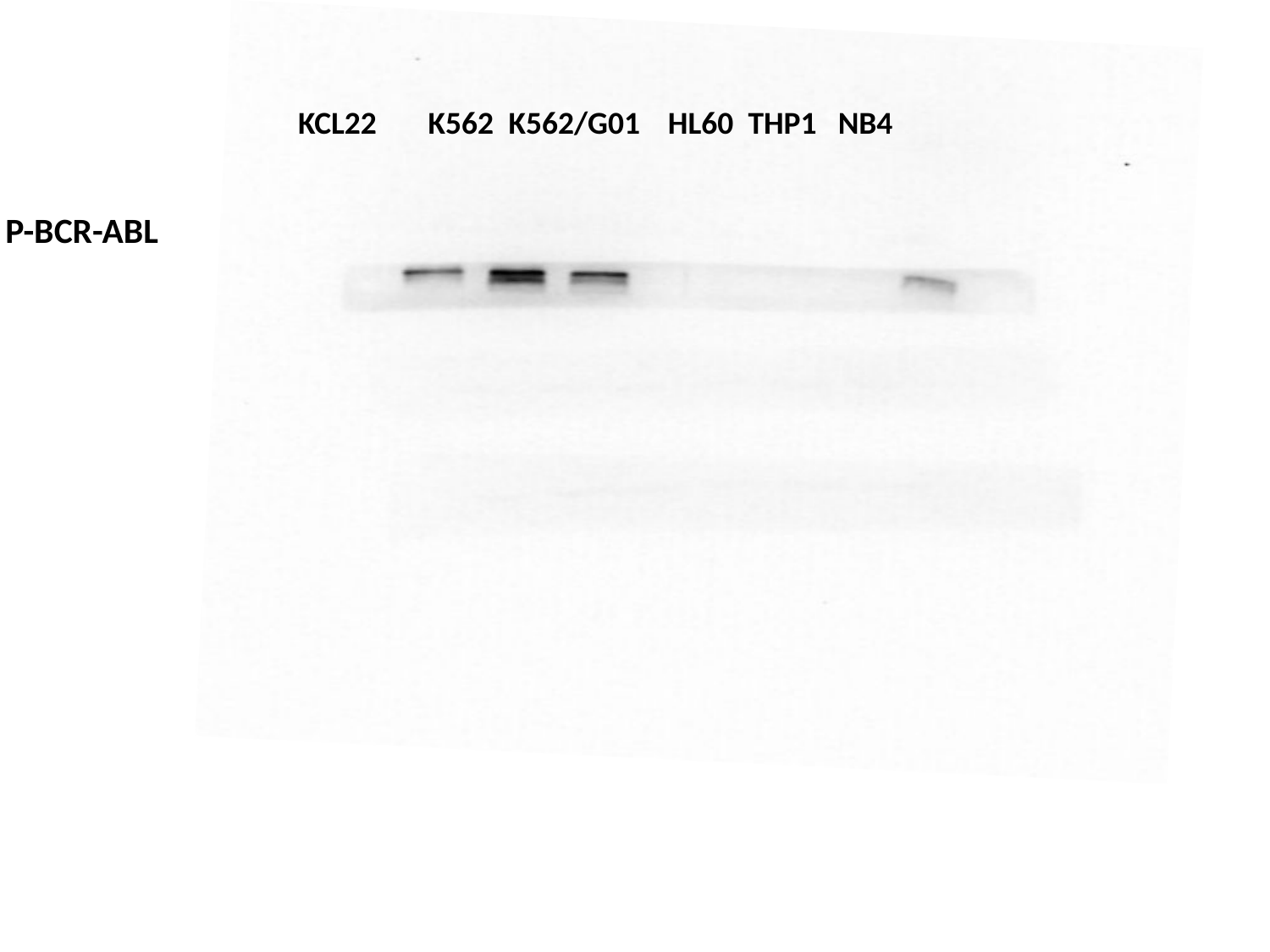

KCL22
K562
K562/G01
HL60
THP1
NB4
P-BCR-ABL

## Slide 2
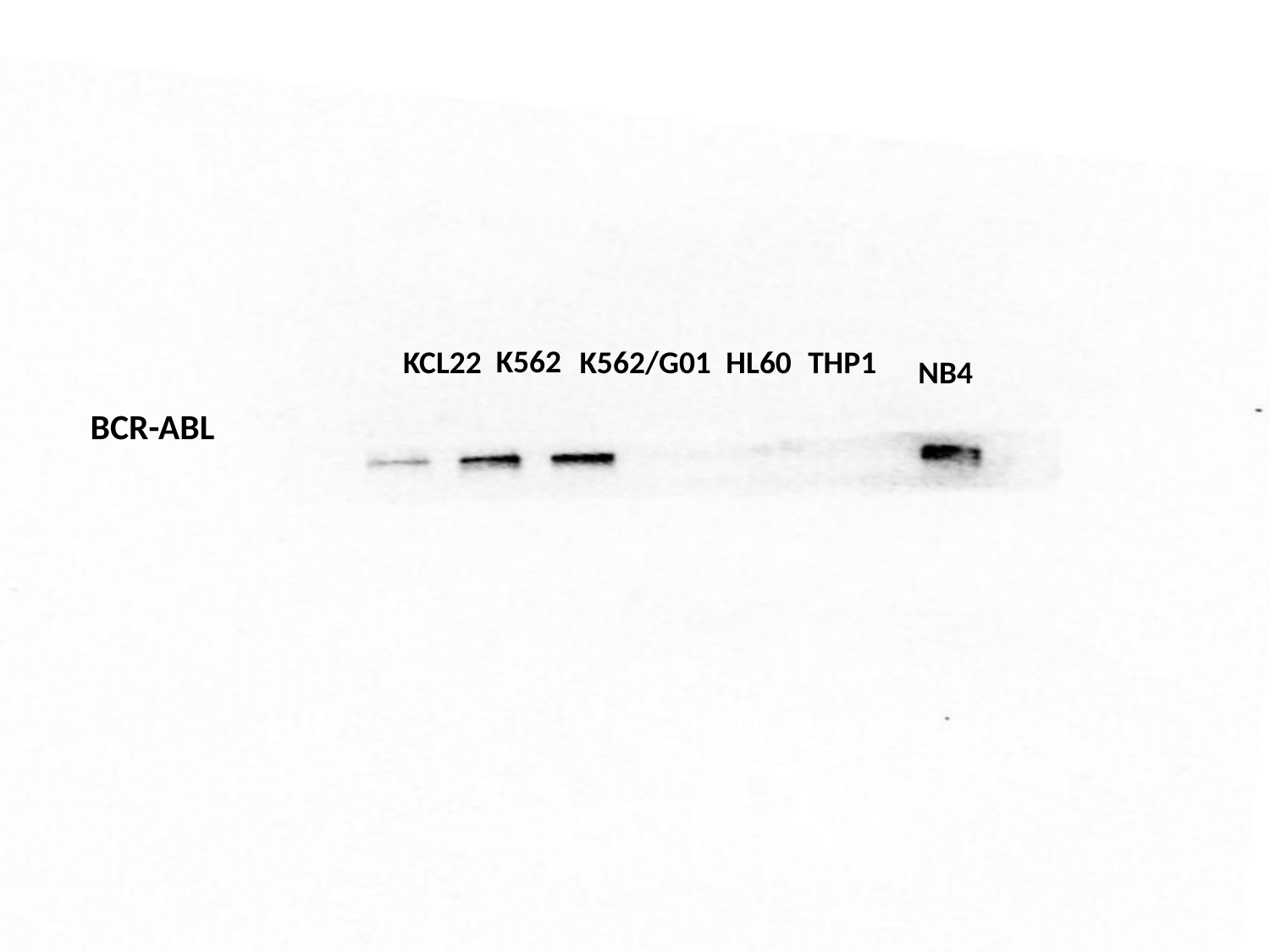

K562
KCL22
K562/G01
HL60
THP1
NB4
BCR-ABL

## Slide 3
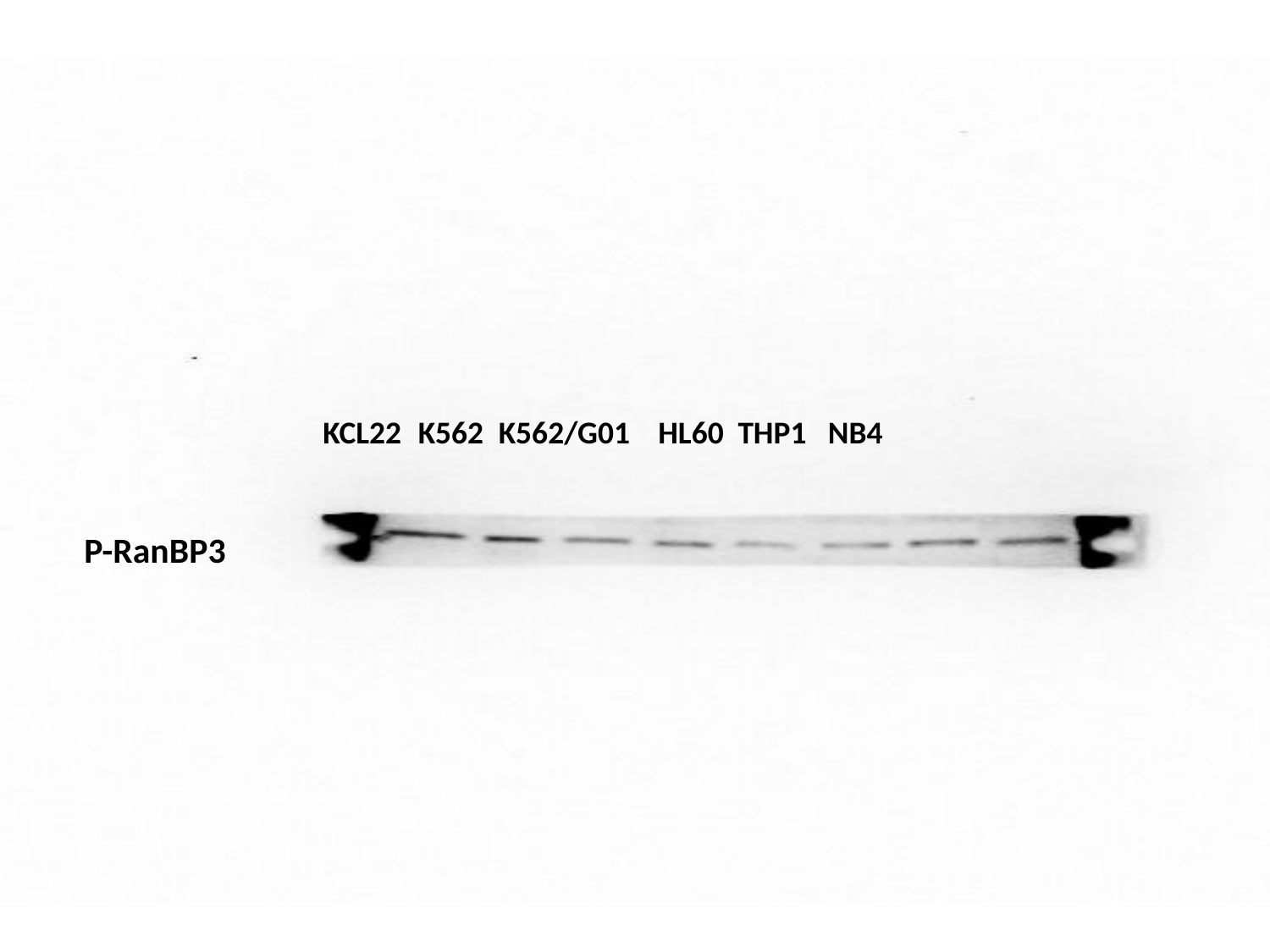

KCL22
K562
K562/G01
HL60
THP1
NB4
P-RanBP3

## Slide 4
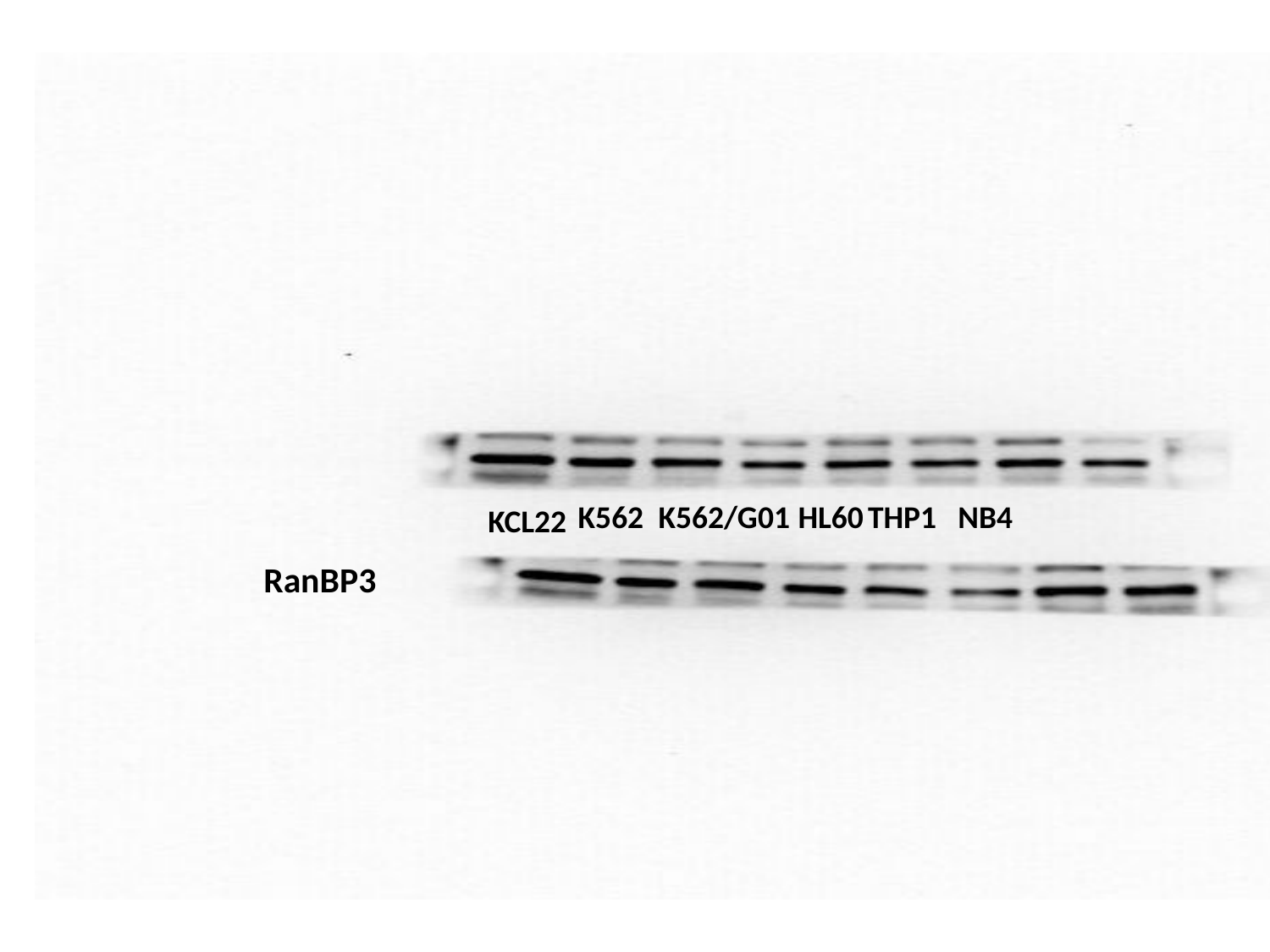

K562
K562/G01
HL60
THP1
NB4
KCL22
RanBP3

## Slide 5
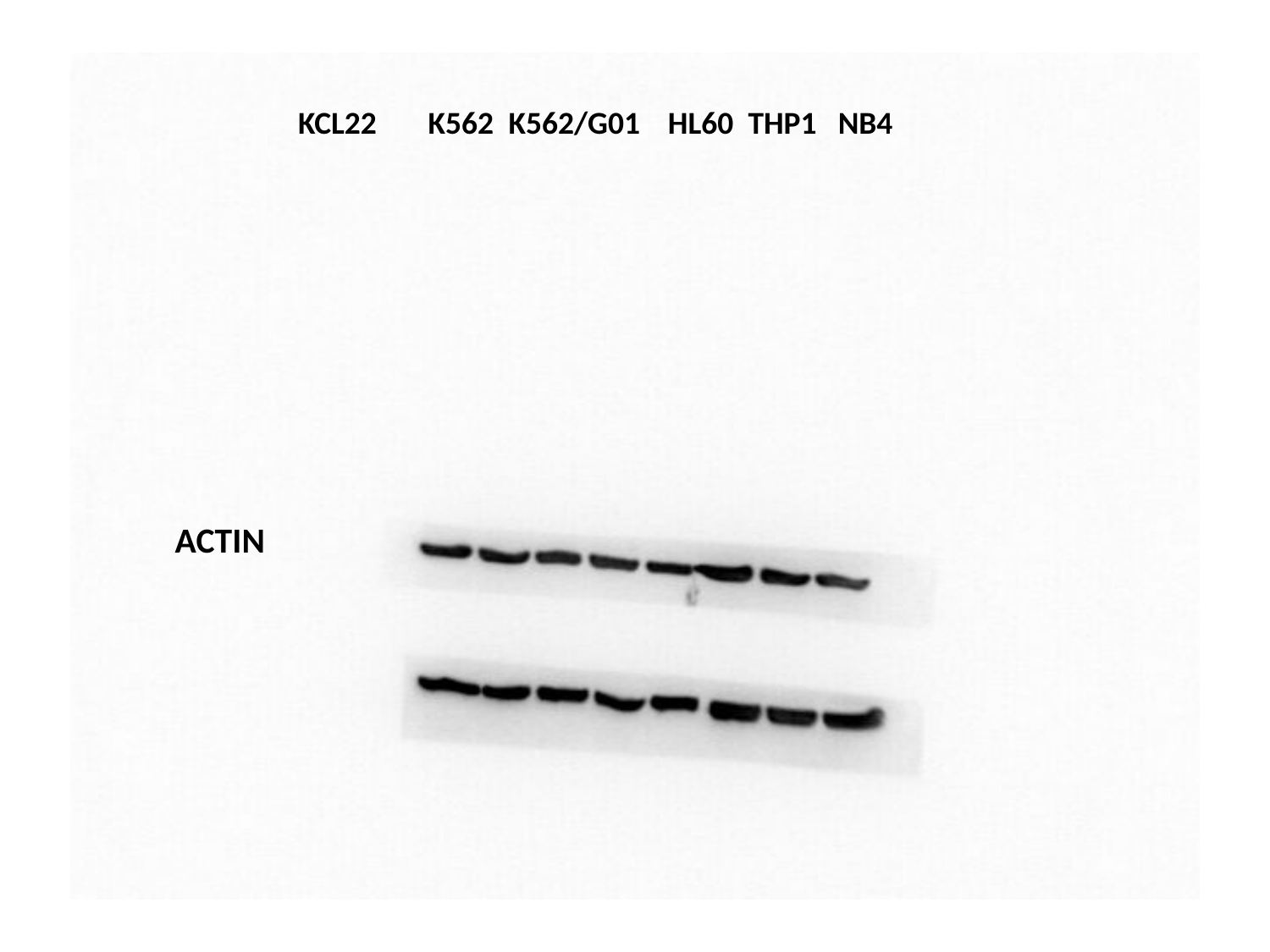

KCL22
K562
K562/G01
HL60
THP1
NB4
ACTIN
