# Supplementary material for: RanBP3 Regulates Proliferation, Apoptosis and Chemosensitivity of Chronic Myeloid Leukemia Cells via Mediating SMAD2/3 and ERK1/2 Nuclear Transport
Source: Front Oncol. 2021 Aug 24;11:698410. doi: 10.3389/fonc.2021.698410 (PMC8421687; doi:10.3389/fonc.2021.698410)
Supplement: Supplementary file 2 [file DataSheet_2.zip › Figure 1 original data/1E.pptx]

## Slide 1
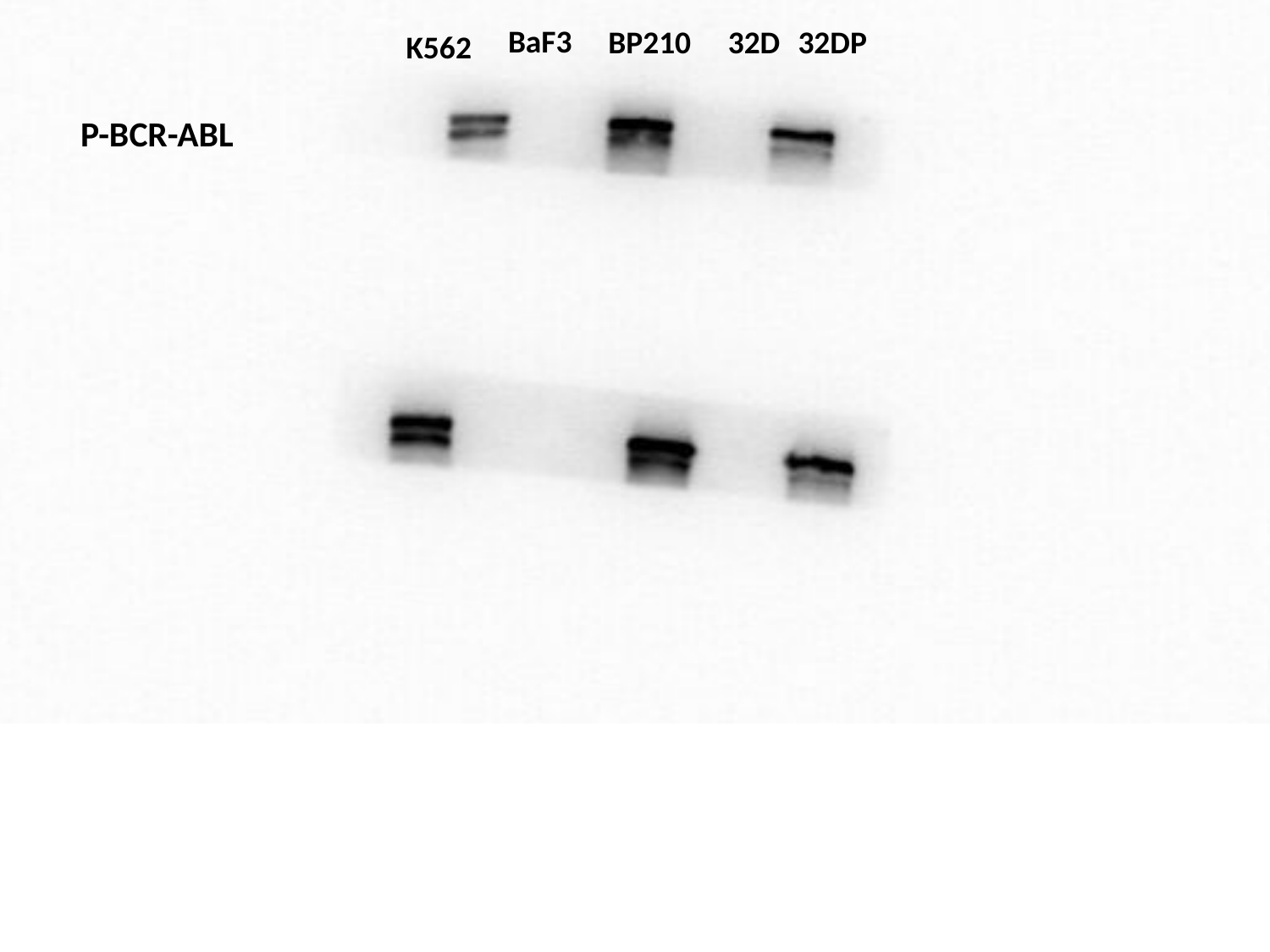

BaF3
BP210
32D
32DP
K562
P-BCR-ABL

## Slide 2
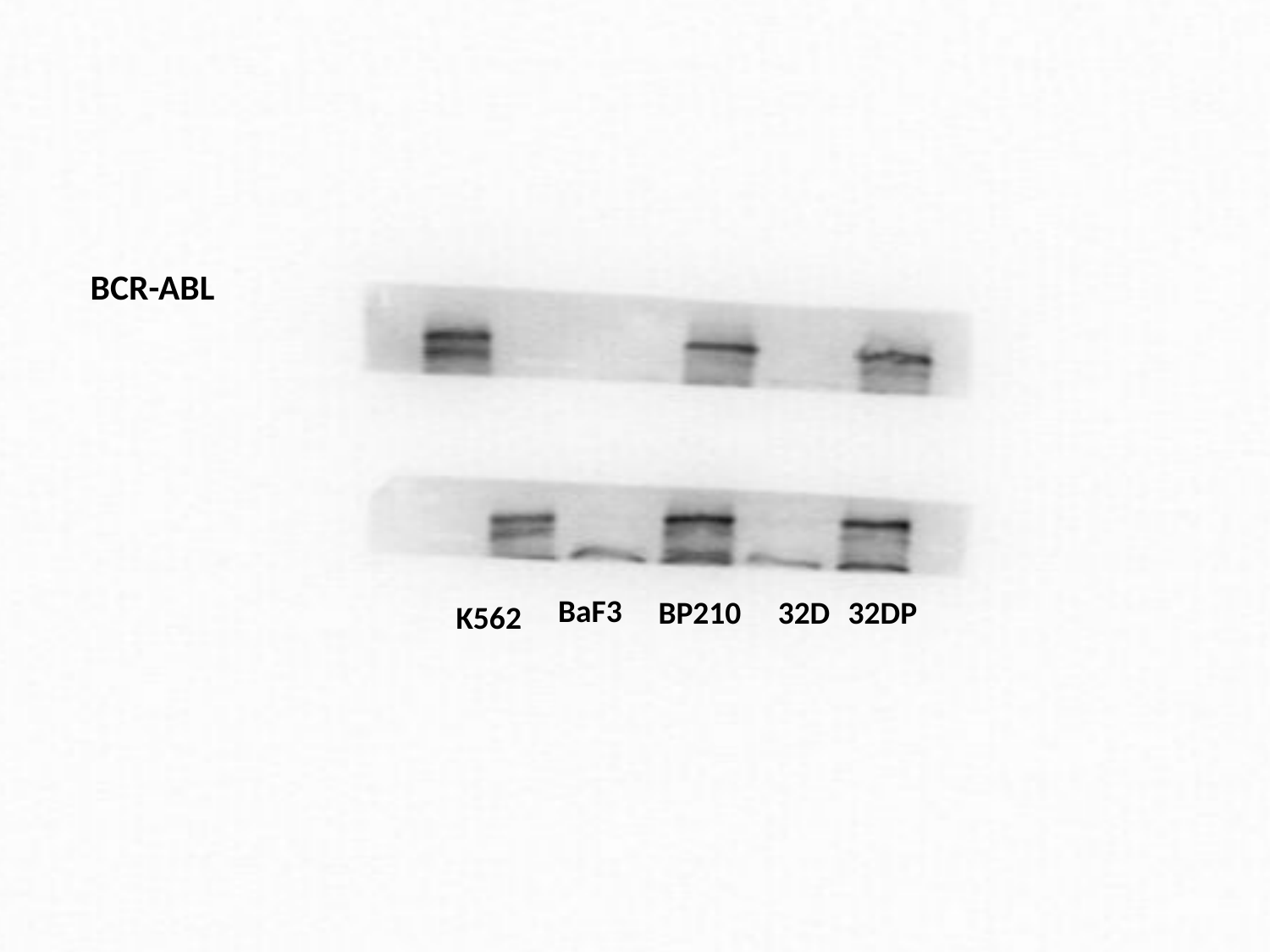

BCR-ABL
BaF3
BP210
32D
32DP
K562

## Slide 3
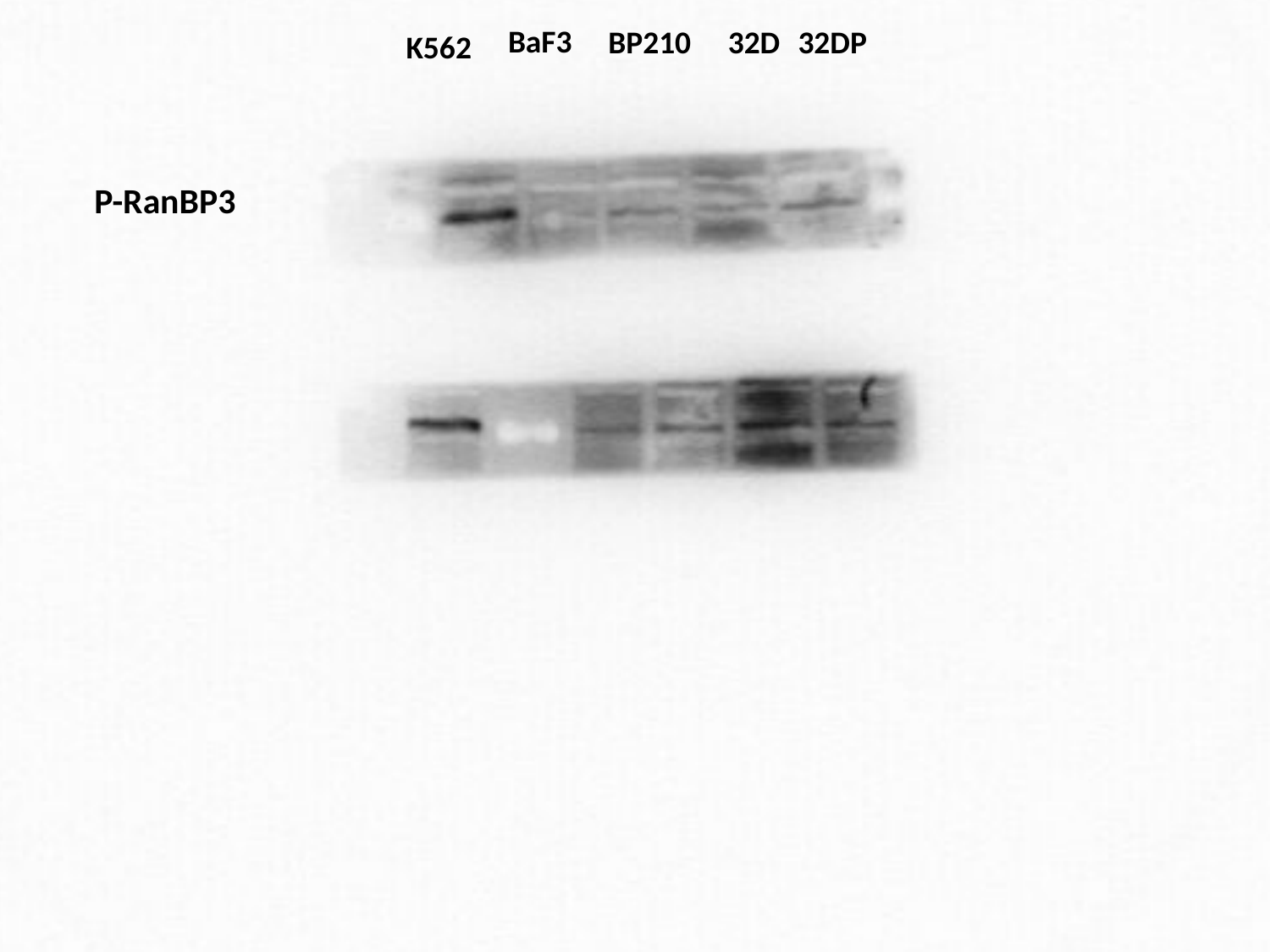

BaF3
BP210
32D
32DP
K562
P-RanBP3

## Slide 4
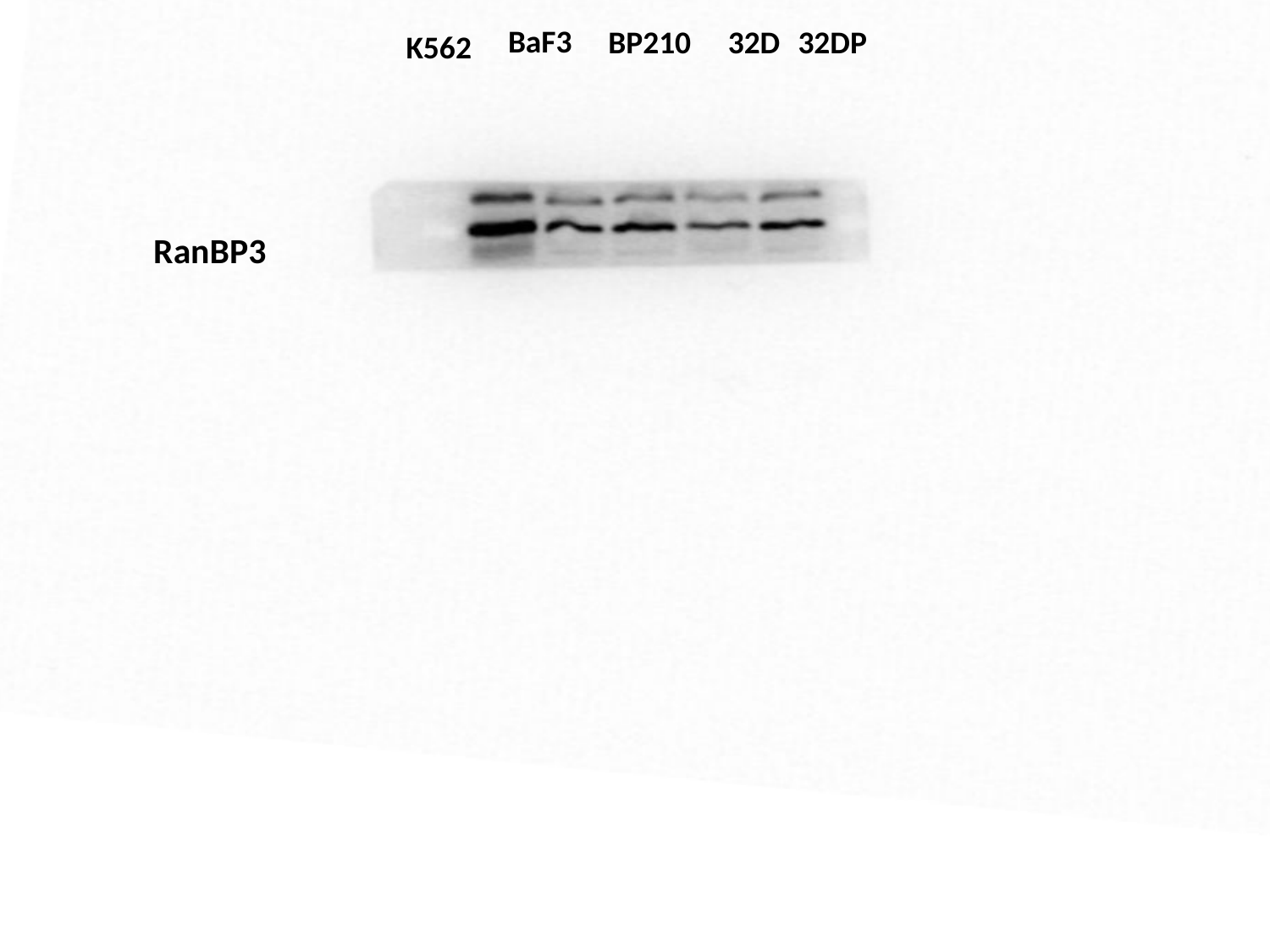

BaF3
BP210
32D
32DP
K562
RanBP3

## Slide 5
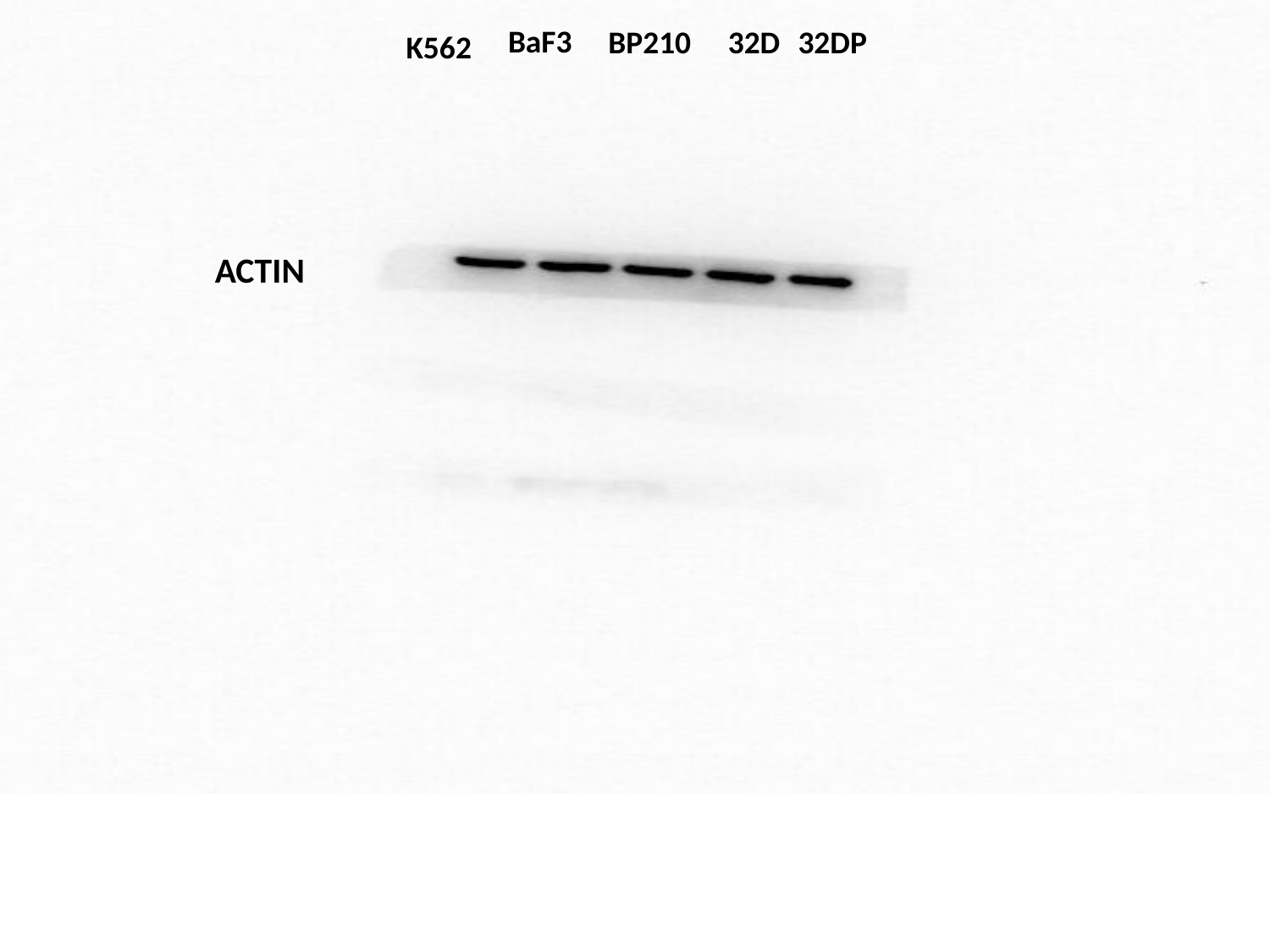

BaF3
BP210
32D
32DP
K562
ACTIN
