# Supplementary material for: RanBP3 Regulates Proliferation, Apoptosis and Chemosensitivity of Chronic Myeloid Leukemia Cells via Mediating SMAD2/3 and ERK1/2 Nuclear Transport
Source: Front Oncol. 2021 Aug 24;11:698410. doi: 10.3389/fonc.2021.698410 (PMC8421687; doi:10.3389/fonc.2021.698410)
Supplement: Supplementary file 2 [file DataSheet_2.zip › Figure 1 original data/1G.pptx]

## Slide 1
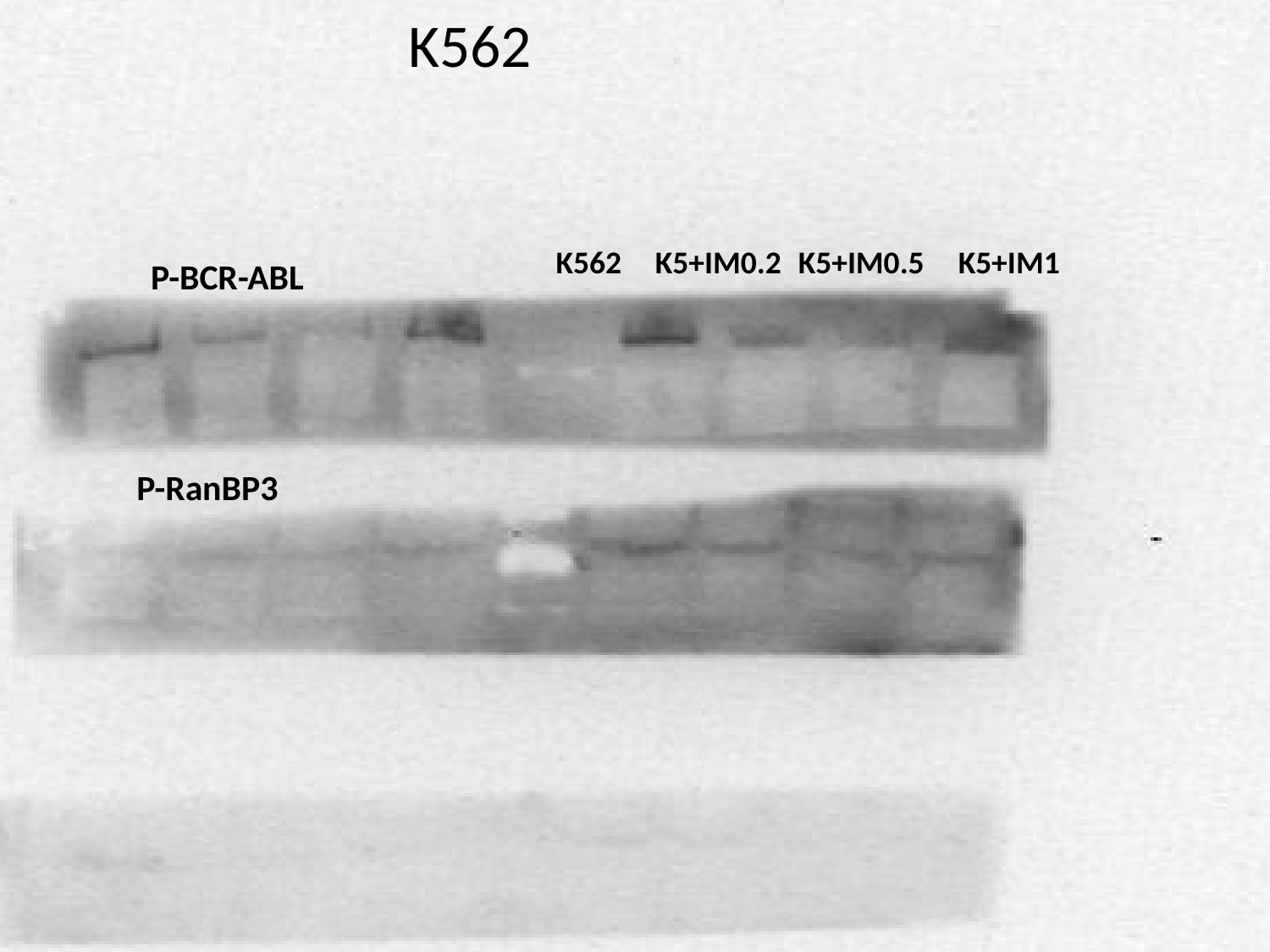

# K562
K562
K5+IM0.2
K5+IM0.5
K5+IM1
P-BCR-ABL
P-RanBP3

## Slide 2
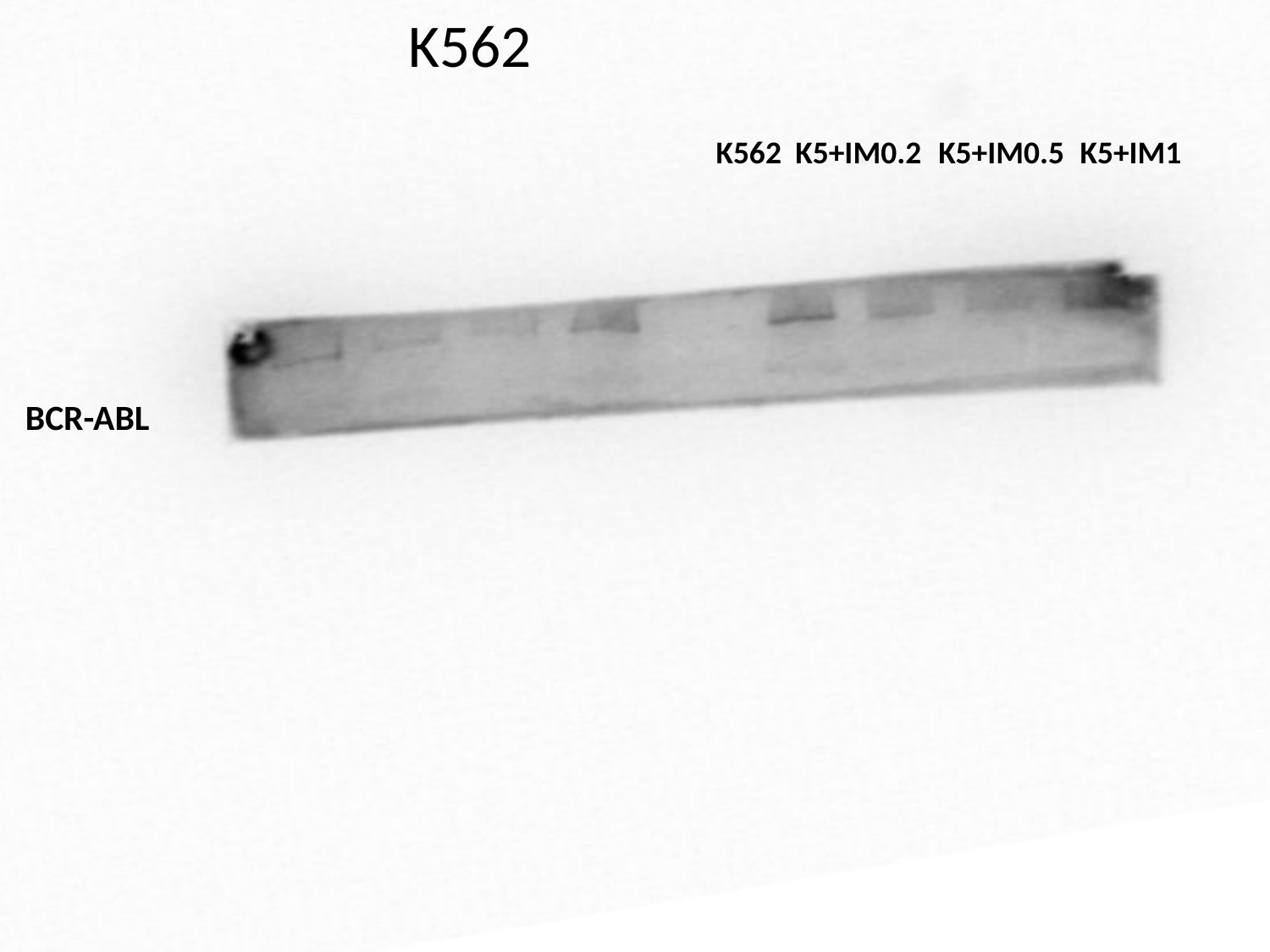

K562
K562
K5+IM0.2
K5+IM0.5
K5+IM1
BCR-ABL

## Slide 3
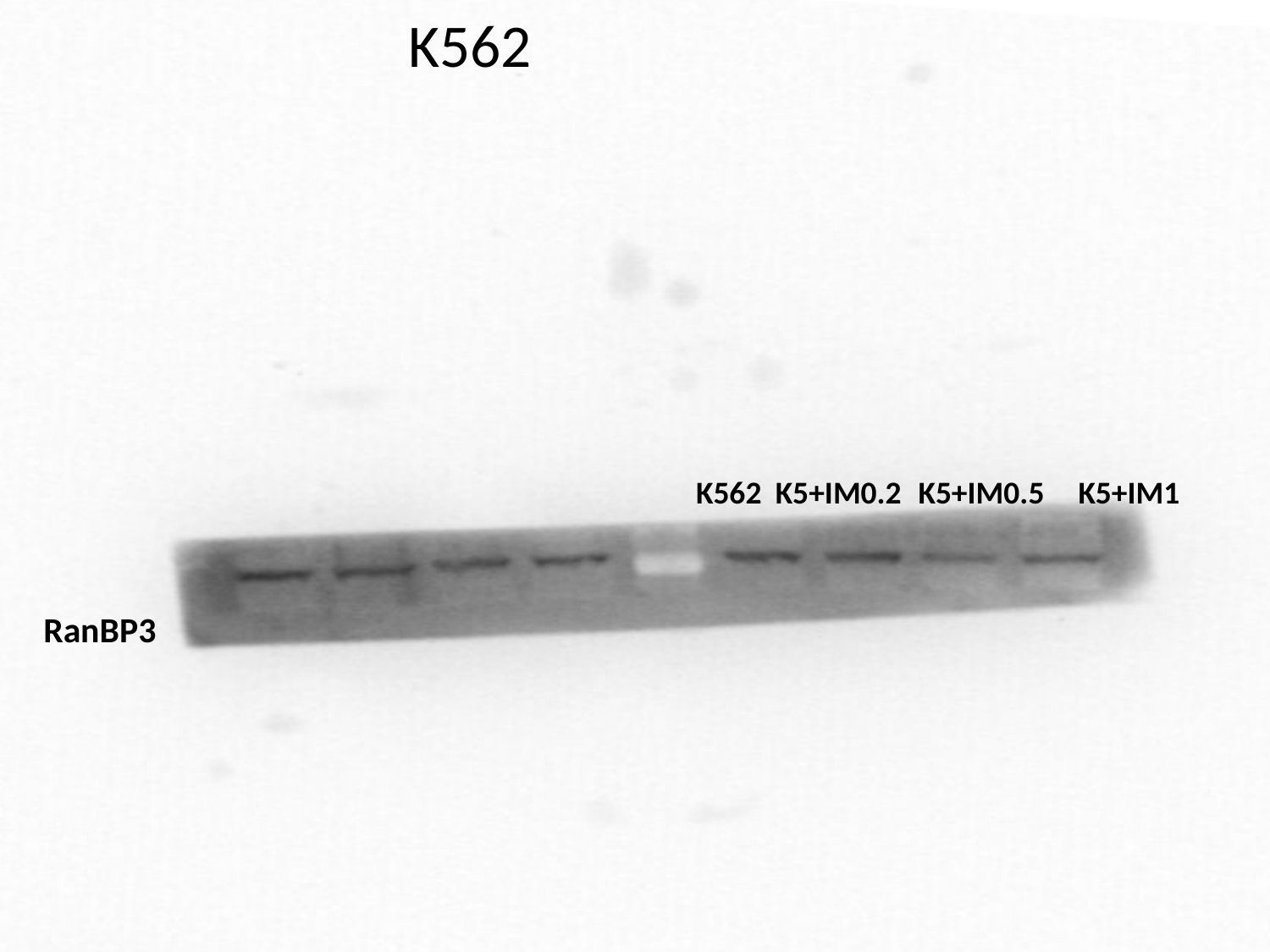

K562
K562
K5+IM0.2
K5+IM0.5
K5+IM1
RanBP3

## Slide 4
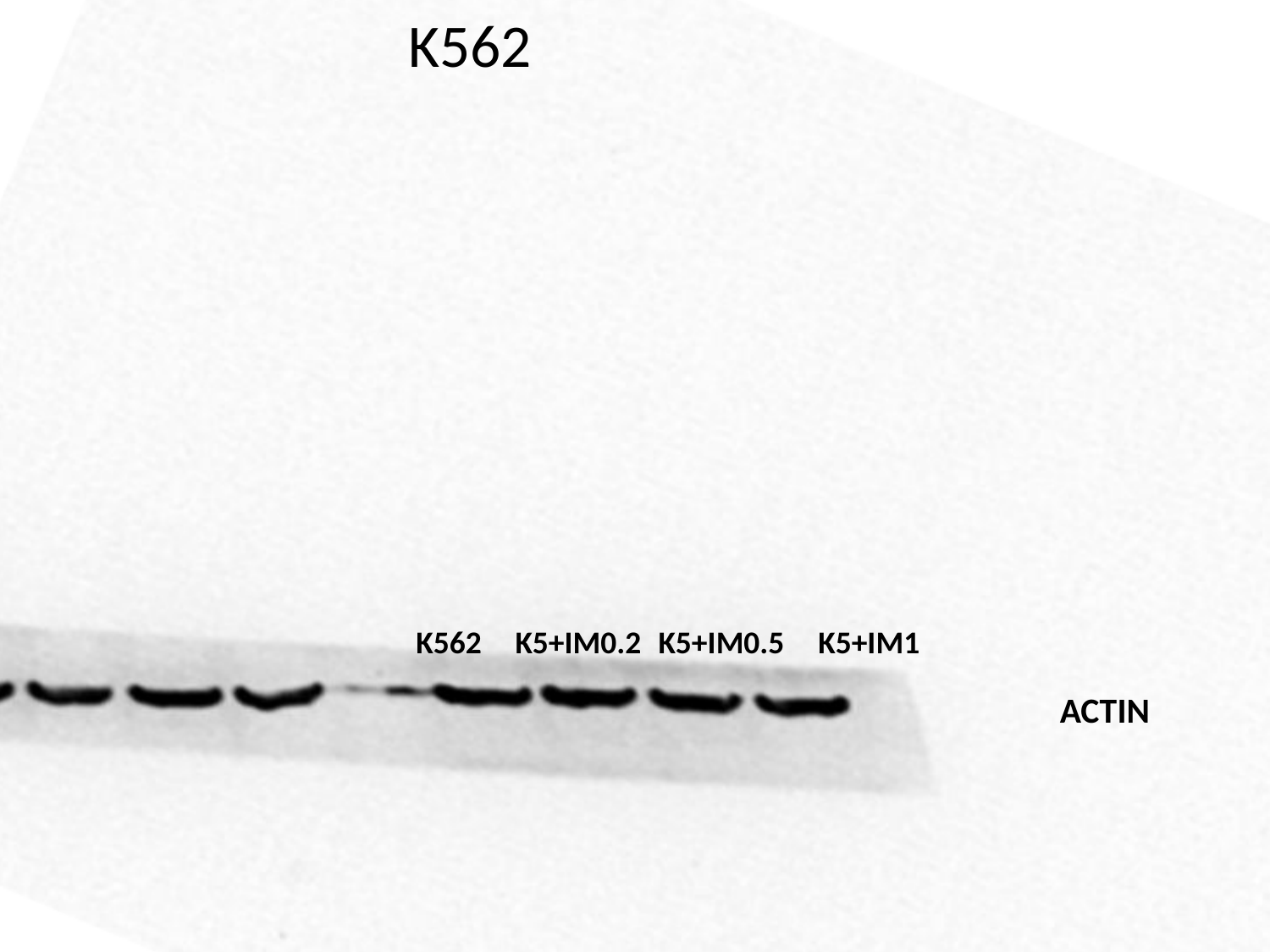

K562
#
K562
K5+IM0.2
K5+IM0.5
K5+IM1
ACTIN

## Slide 5
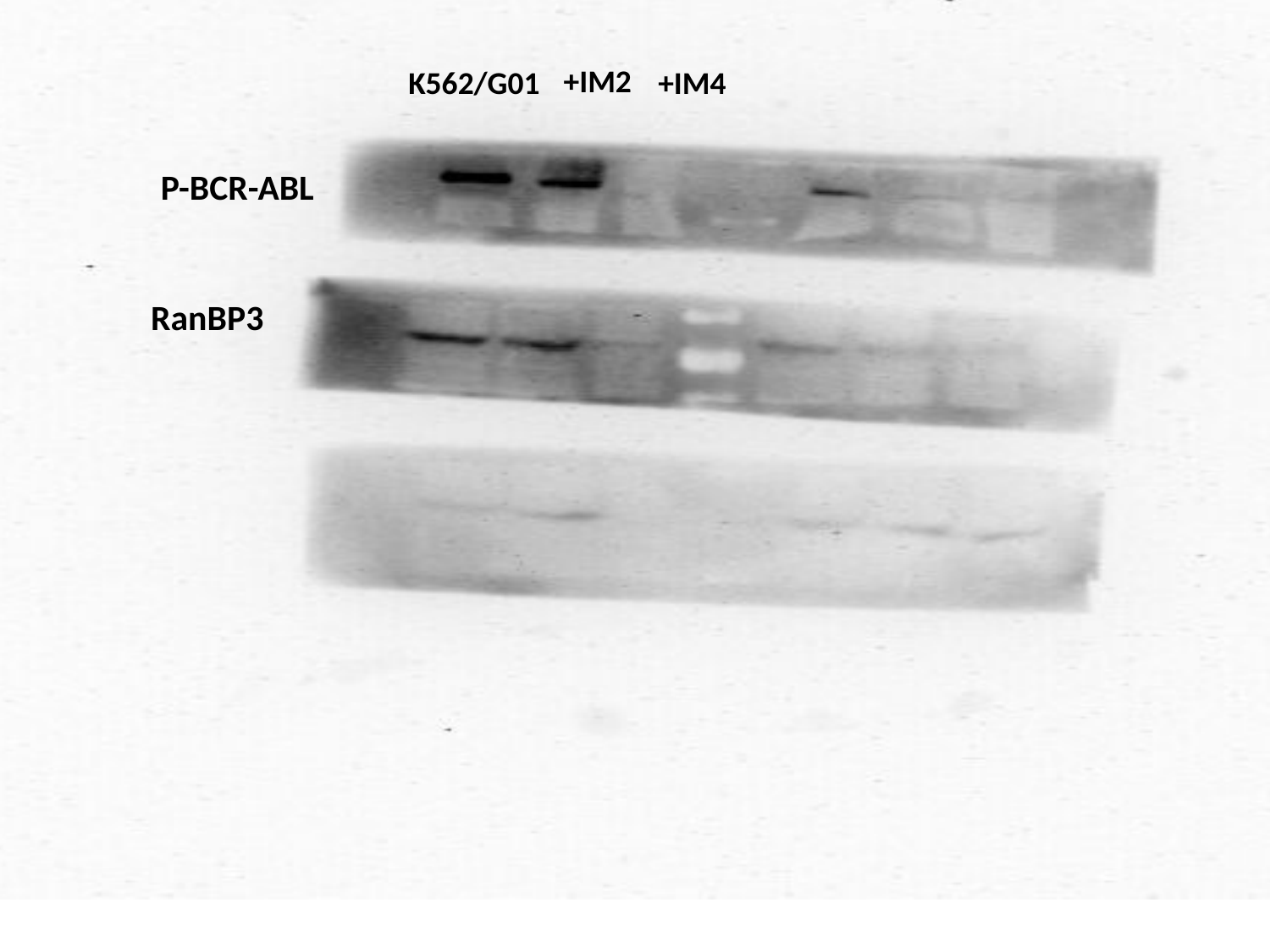

K562/G01
+IM2
K562/G01
+IM4
P-BCR-ABL
RanBP3

## Slide 6
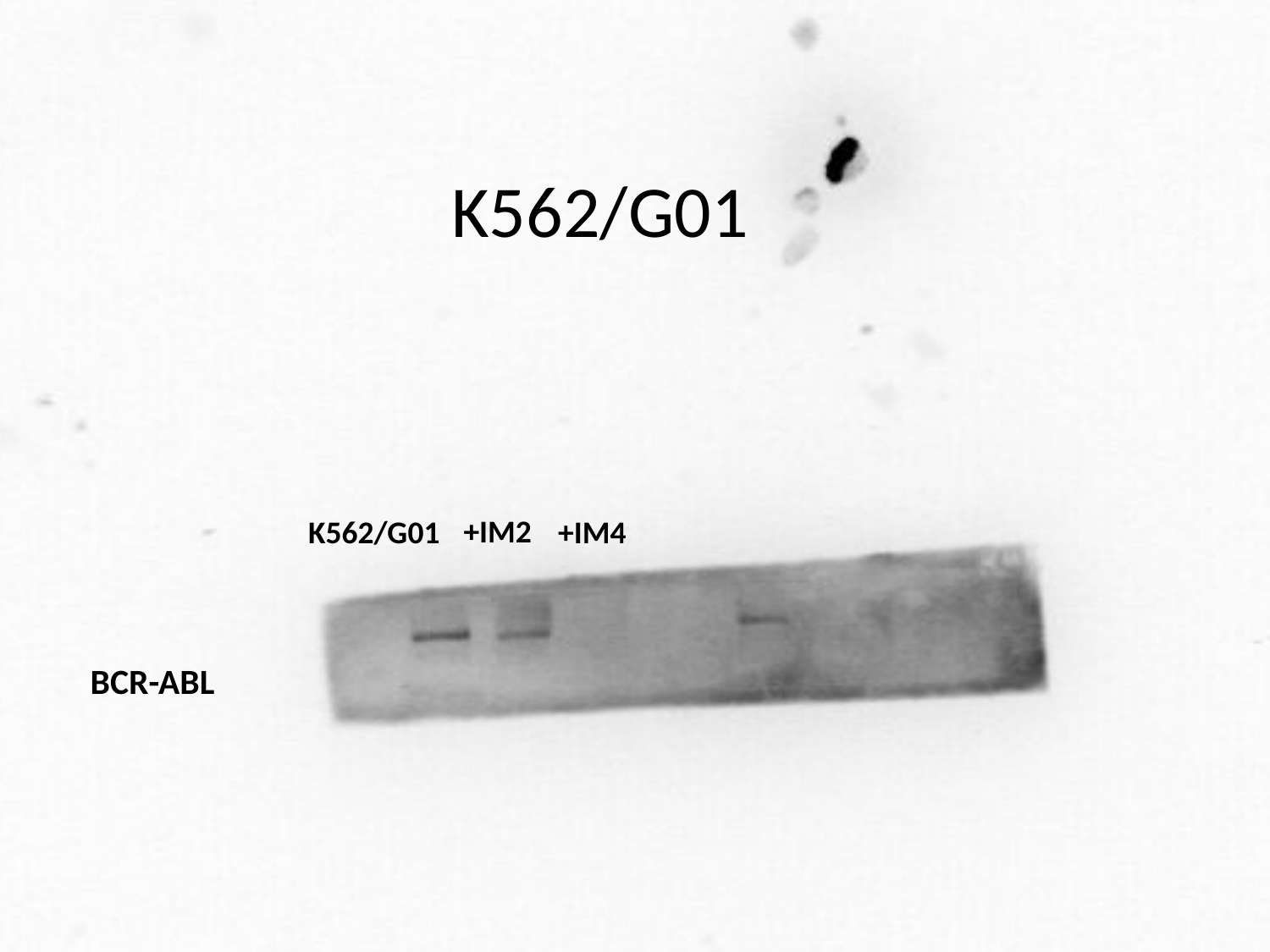

K562/G01
+IM2
K562/G01
+IM4
BCR-ABL

## Slide 7
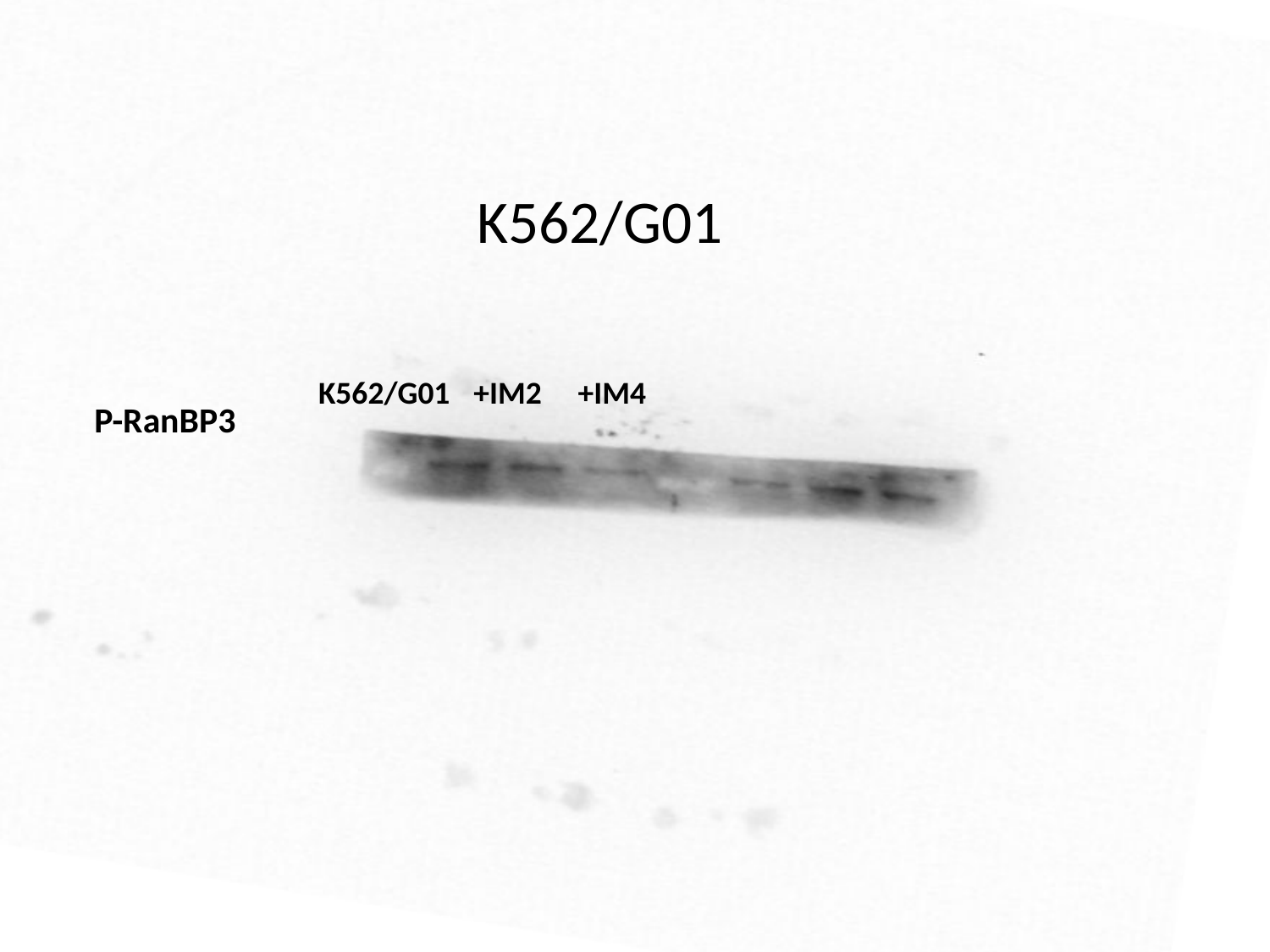

K562/G01
K562/G01
+IM2
+IM4
P-RanBP3

## Slide 8
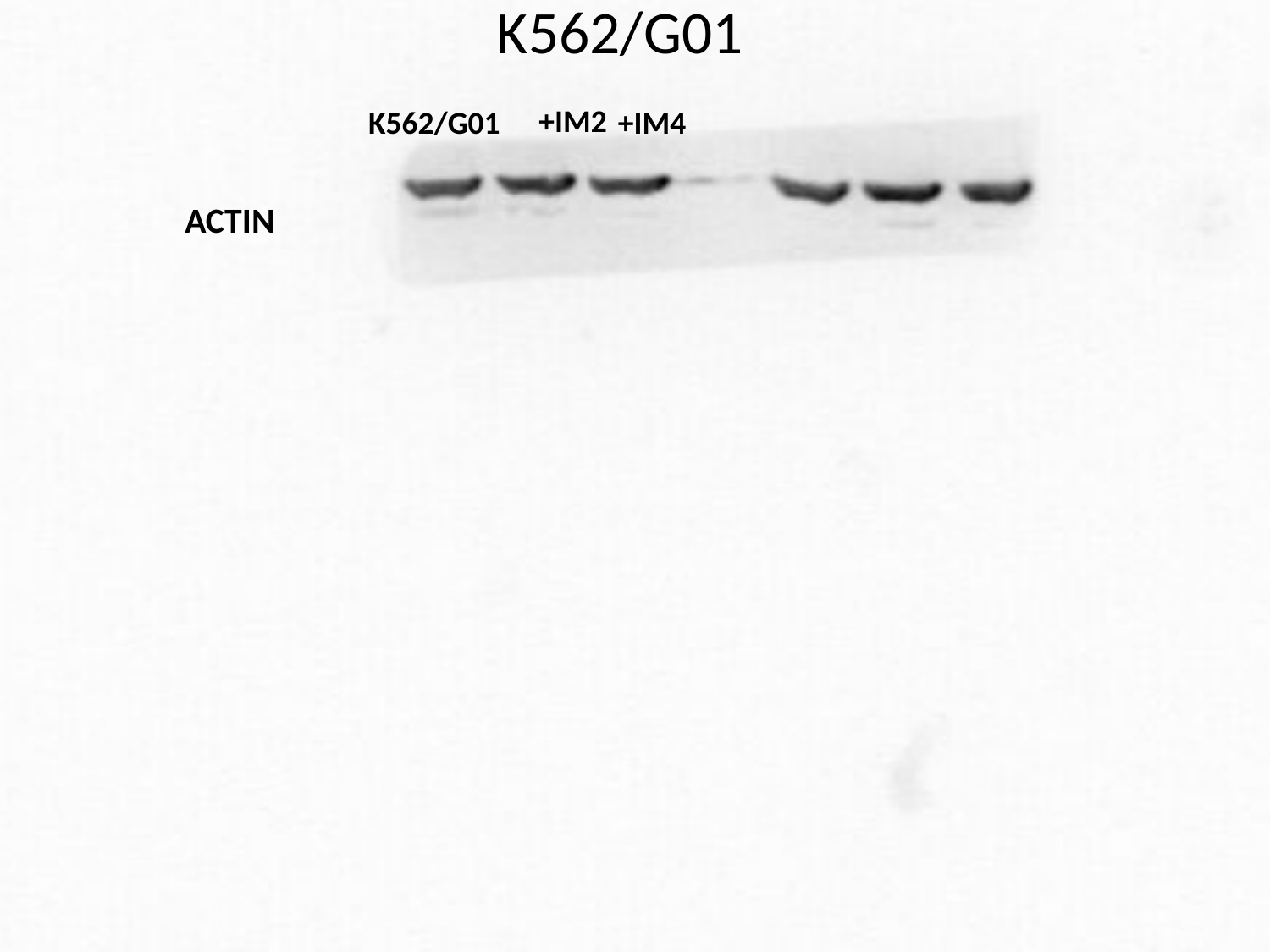

K562/G01
 +IM2
K562/G01
+IM4
ACTIN
