# Supplementary figures and images for: RanBP3 Regulates Proliferation, Apoptosis and Chemosensitivity of Chronic Myeloid Leukemia Cells via Mediating SMAD2/3 and ERK1/2 Nuclear Transport
Source: Front Oncol. 2021 Aug 24;11:698410. doi: 10.3389/fonc.2021.698410 (PMC8421687; doi:10.3389/fonc.2021.698410)

## Slide 1
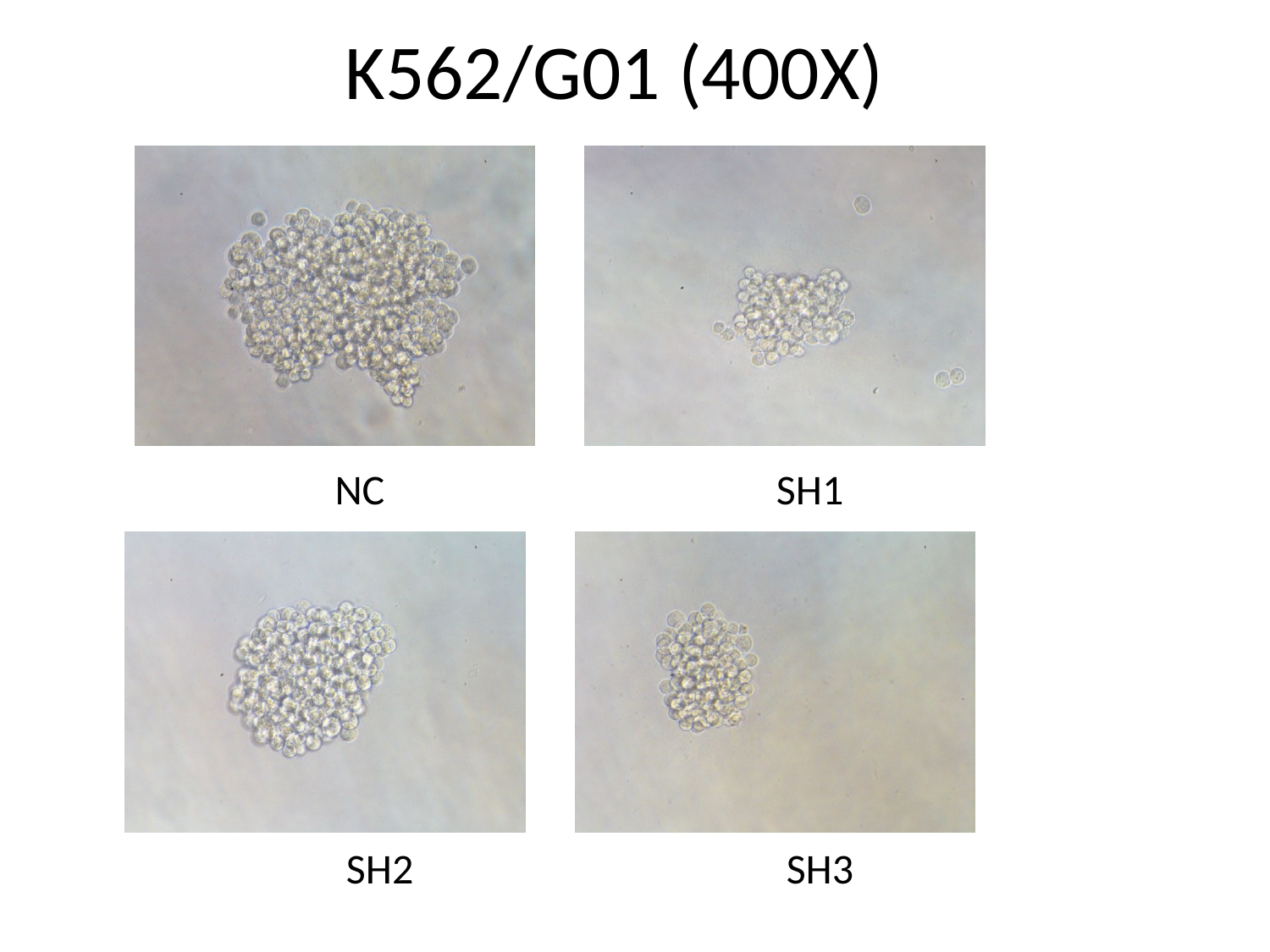

# K562/G01 (400X)
NC
SH1
SH2
SH3

## Slide 2
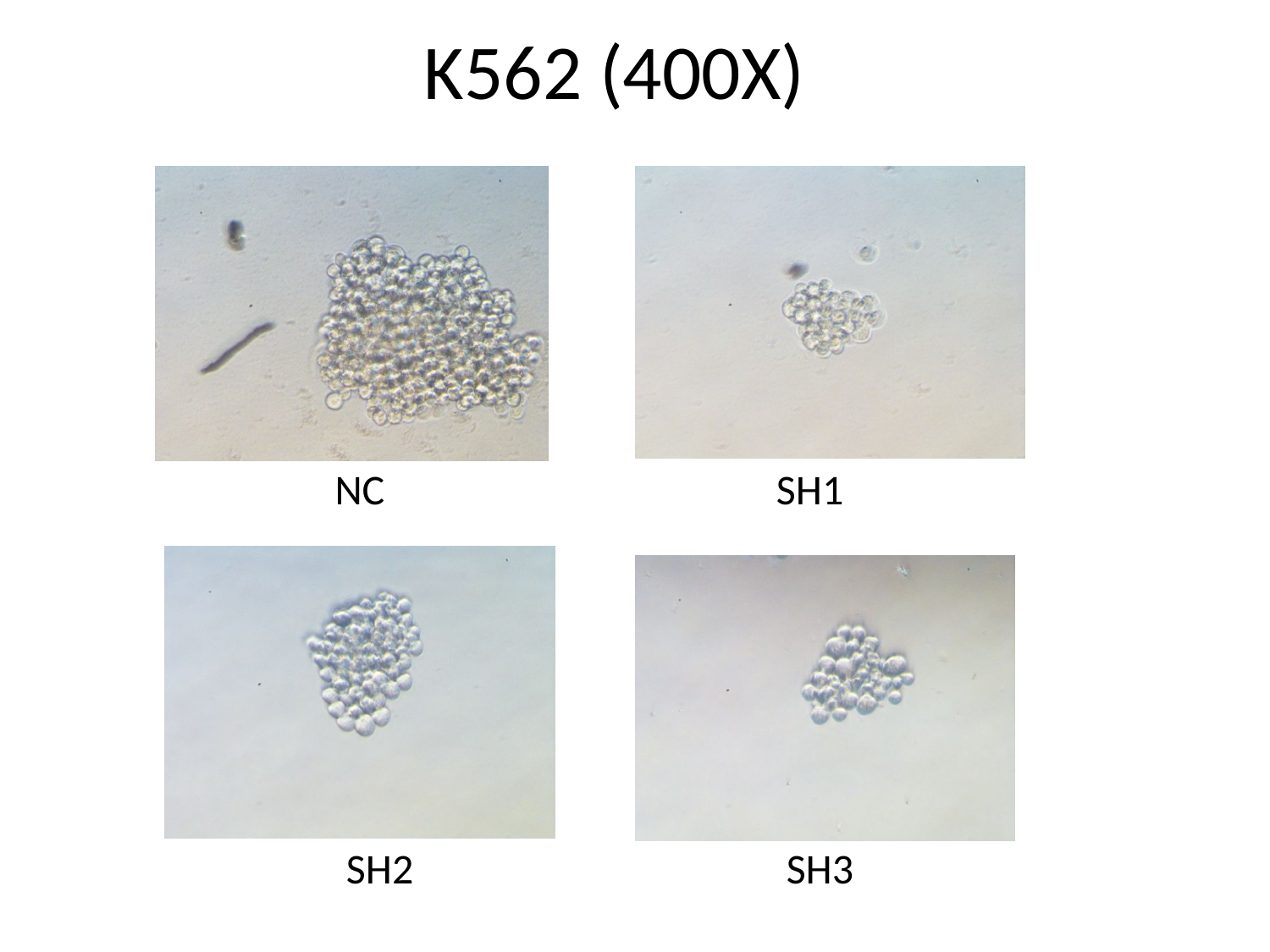

# K562 (400X)
NC
SH1
SH2
SH3

Supplement: Supplementary file 2 [file DataSheet_2.zip › Figure 2 original data/2E.pptx]

## Slide 1
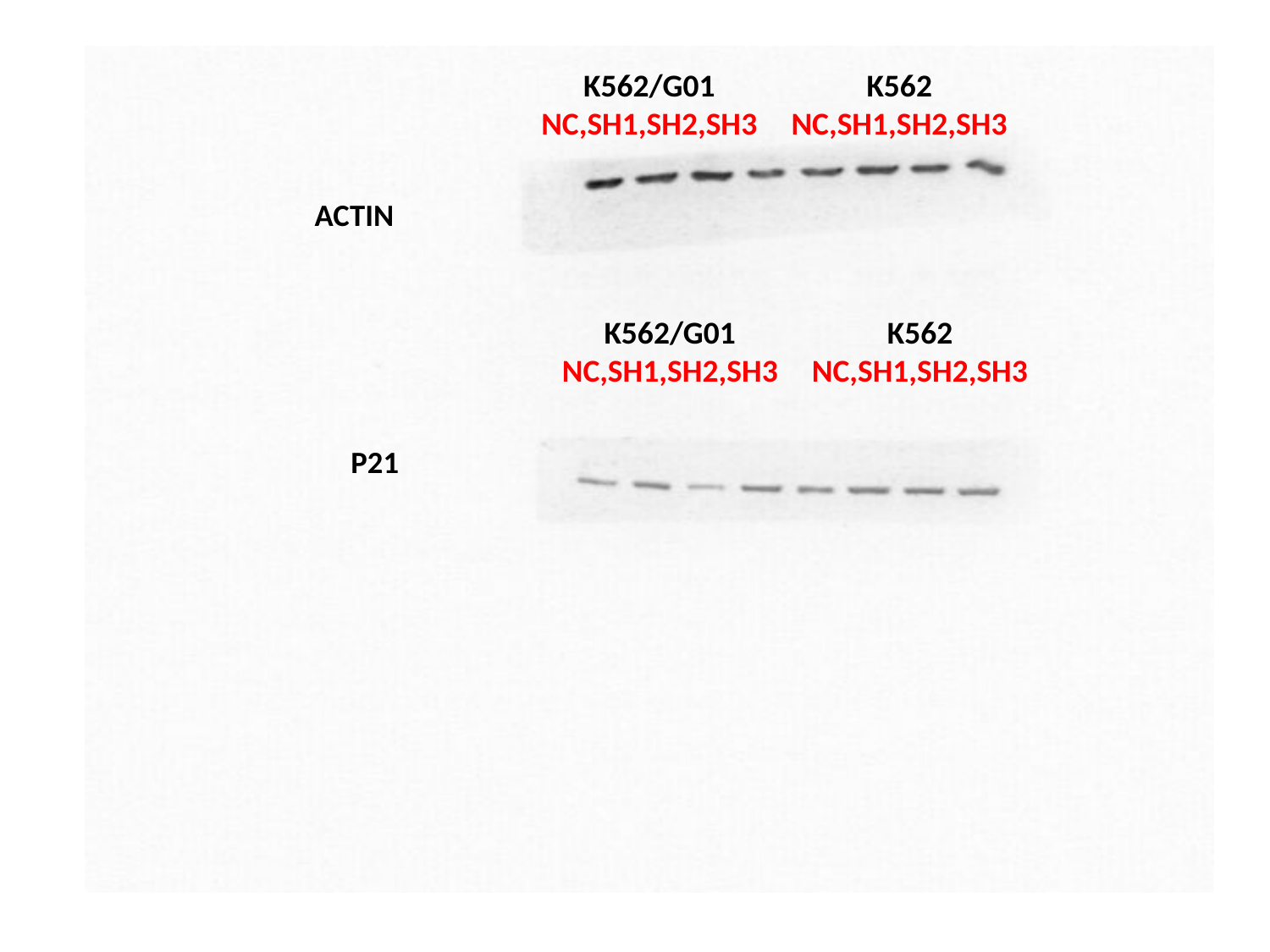

K562/G01
NC,SH1,SH2,SH3
K562
NC,SH1,SH2,SH3
ACTIN
K562/G01
NC,SH1,SH2,SH3
K562
NC,SH1,SH2,SH3
P21

Supplement: Supplementary file 2 [file DataSheet_2.zip › Figure 2 original data/2G.pptx]

## Slide 1
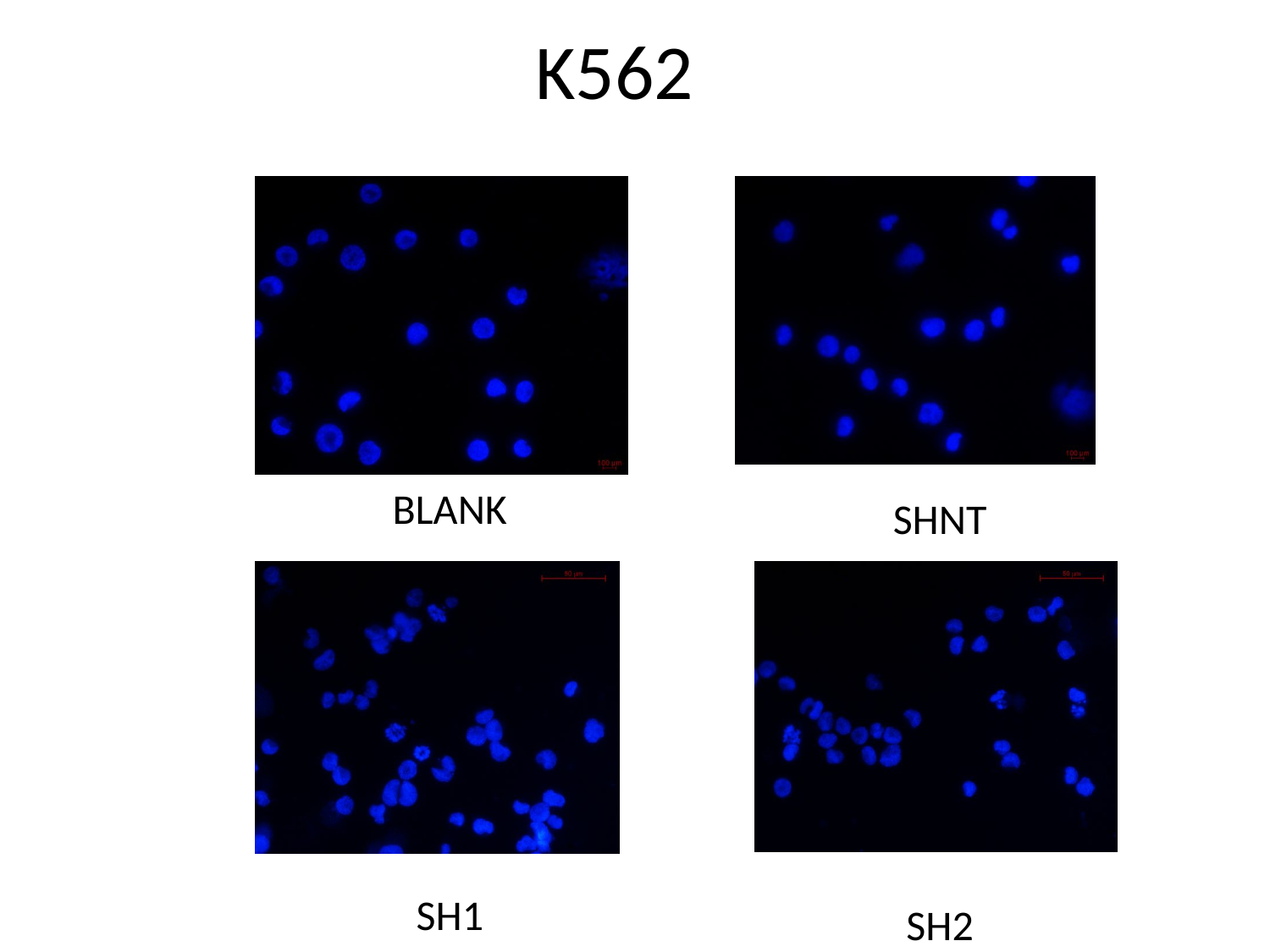

# K562
BLANK
SHNT
SH1
SH2

## Slide 2
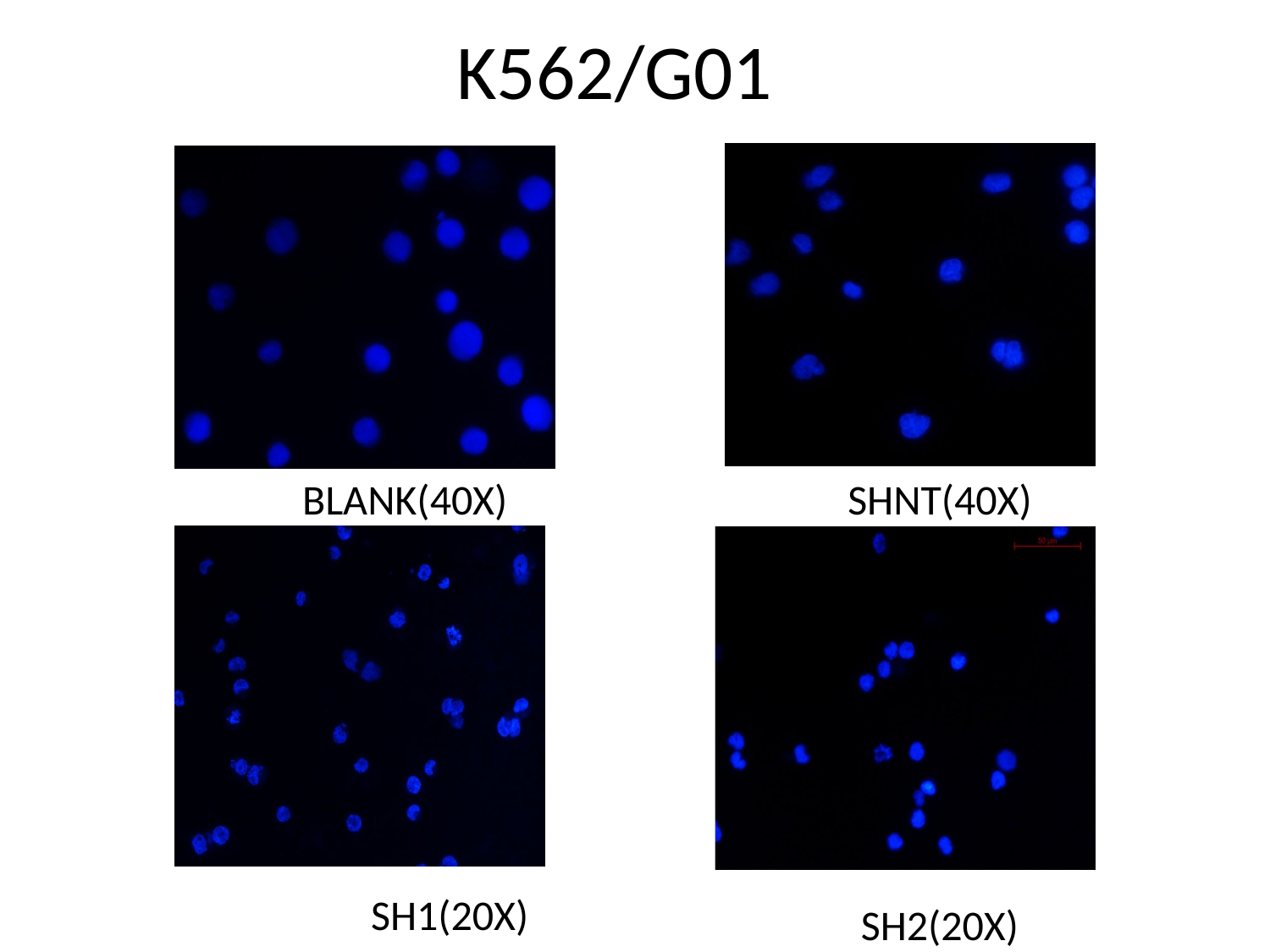

# K562/G01
BLANK(40X)
SHNT(40X)
SH1(20X)
SH2(20X)

Supplement: Supplementary file 2 [file DataSheet_2.zip › Figure 3 original data/3C.pptx]

## ShNT

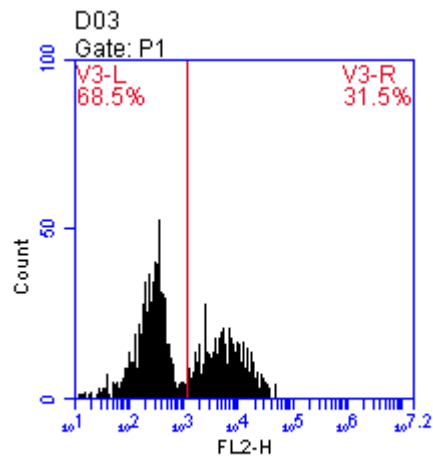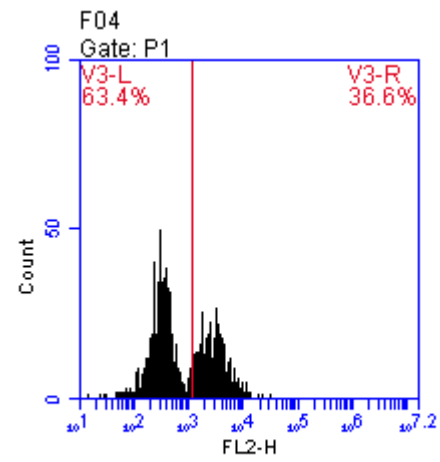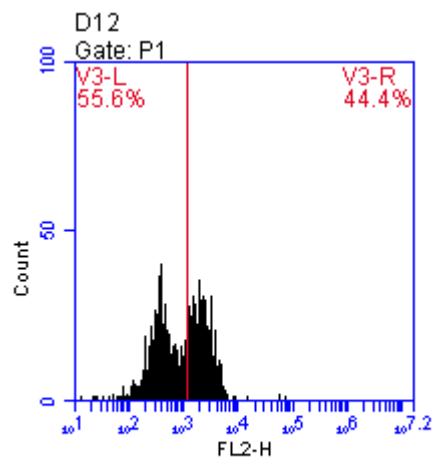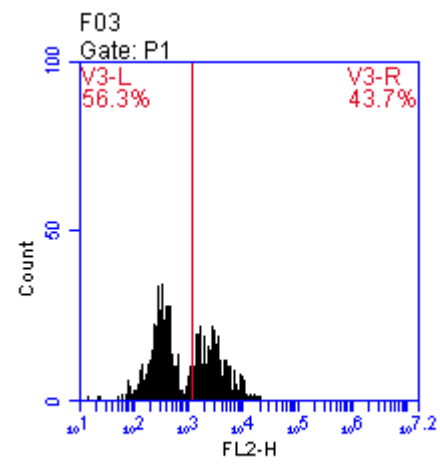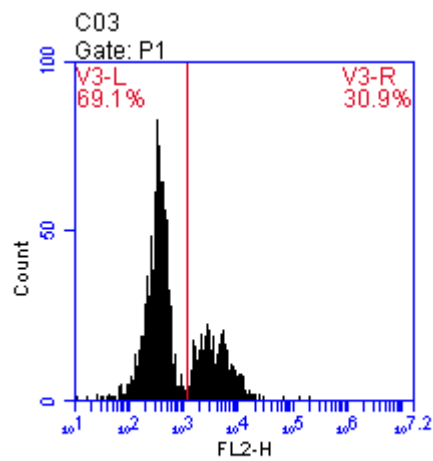

# ShRanBP3#1

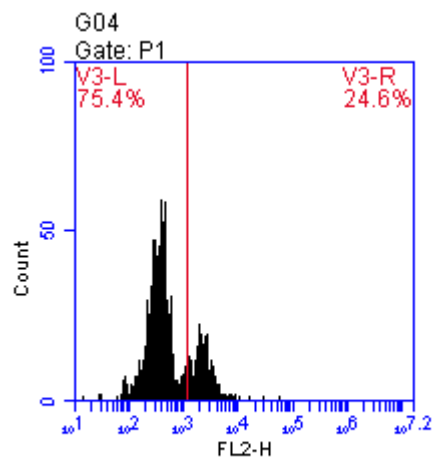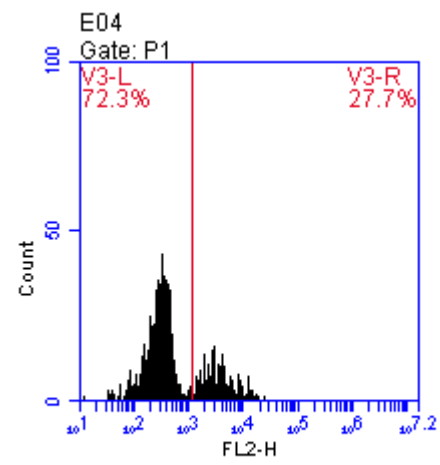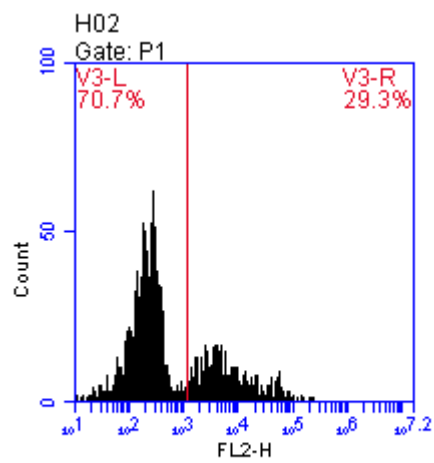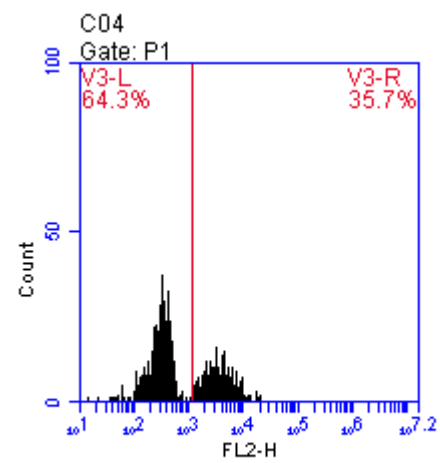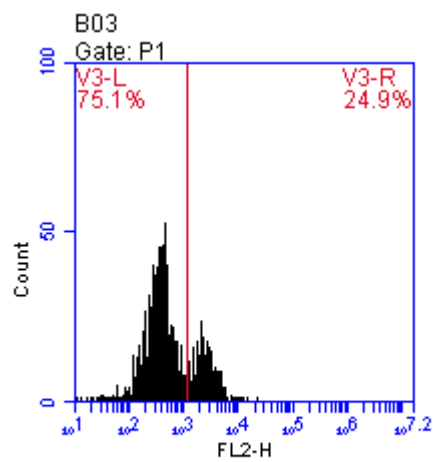

# ShNT+IM

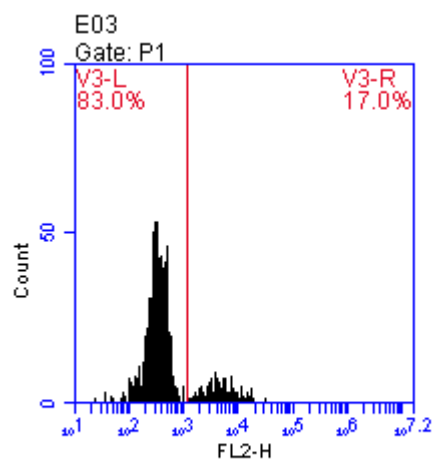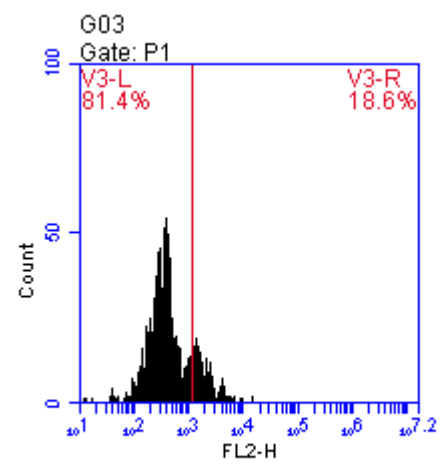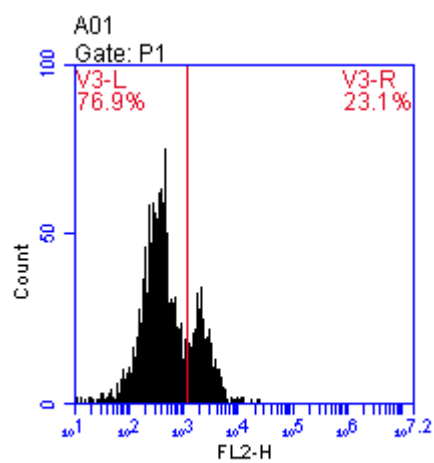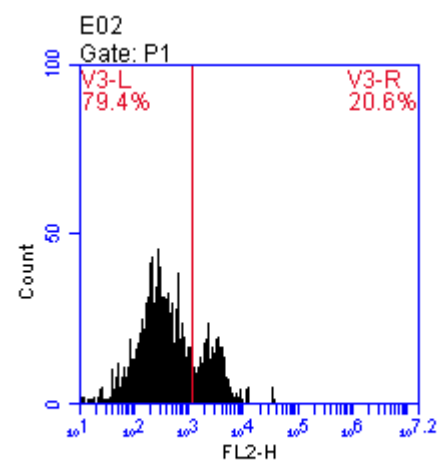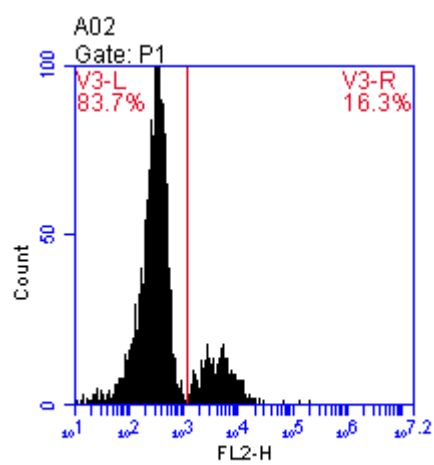

# ShRanBP3#1+IM

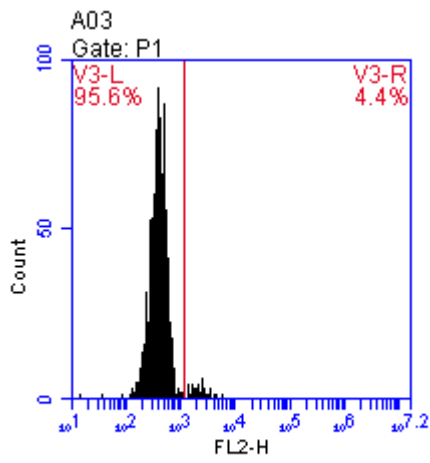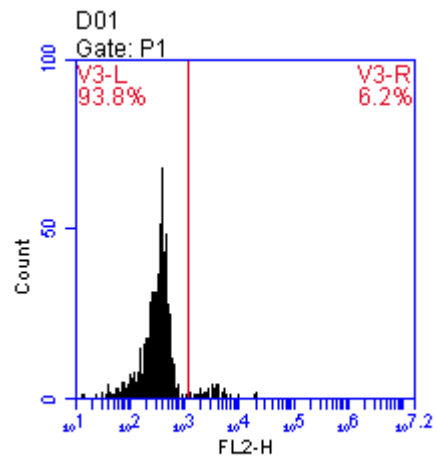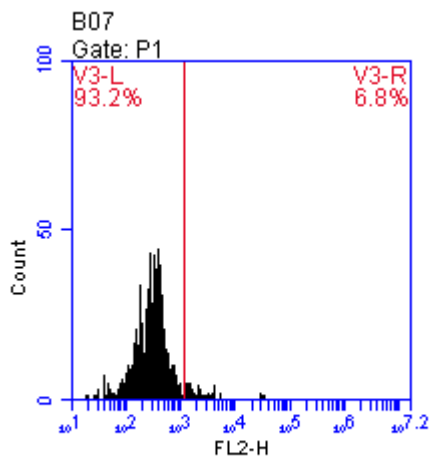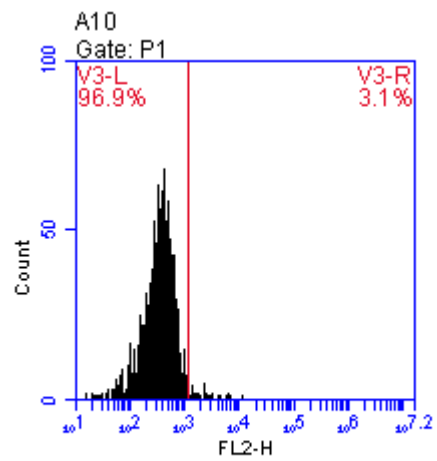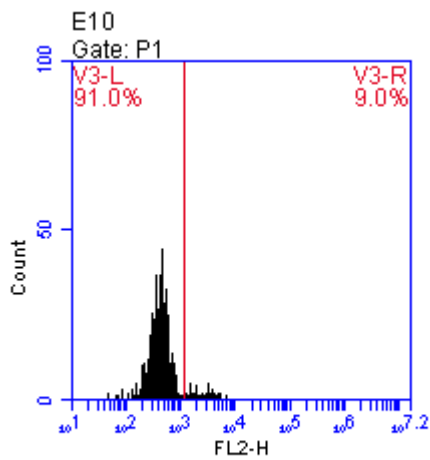

Supplement: Supplementary file 3 [file DataSheet_3.zip › Figure 5 original data/5B.pdf]

## Slide 1
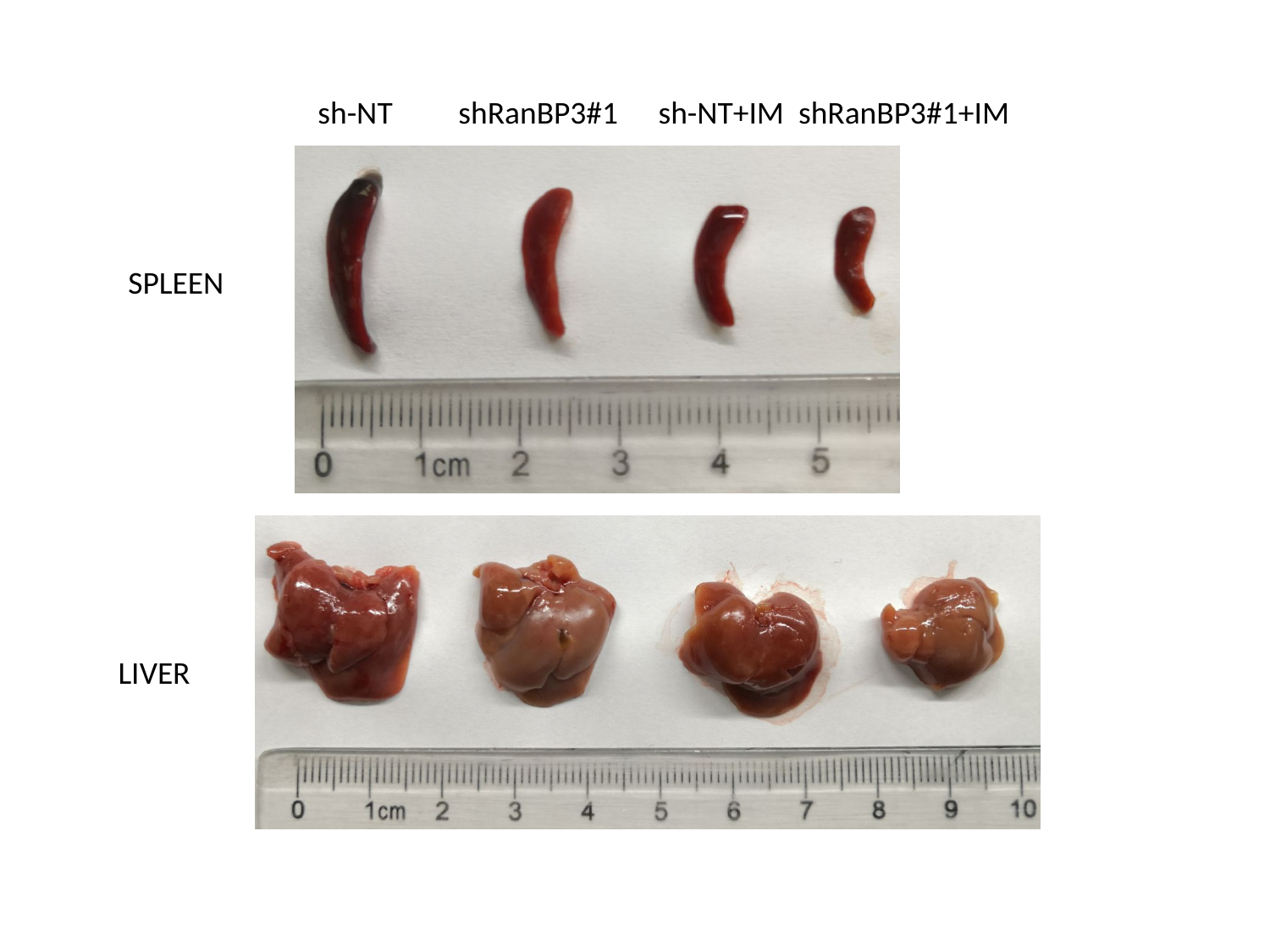

sh-NT
shRanBP3#1
sh-NT+IM
shRanBP3#1+IM
SPLEEN
LIVER

Supplement: Supplementary file 3 [file DataSheet_3.zip › Figure 5 original data/5C.pptx]

## Slide 1
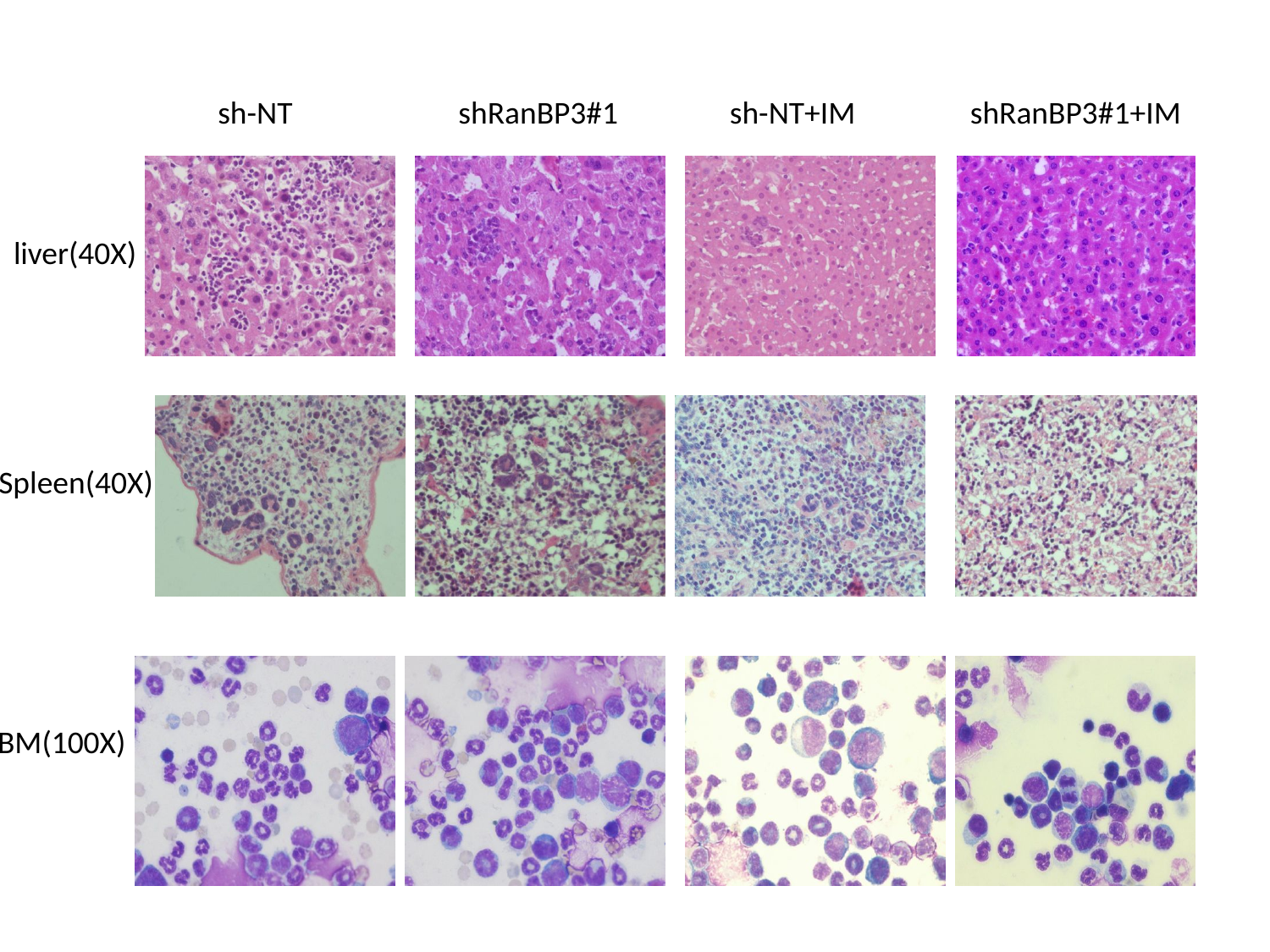

sh-NT
shRanBP3#1
sh-NT+IM
shRanBP3#1+IM
liver(40X)
Spleen(40X)
BM(100X)

Supplement: Supplementary file 3 [file DataSheet_3.zip › Figure 5 original data/5F.pptx]

## Slide 1
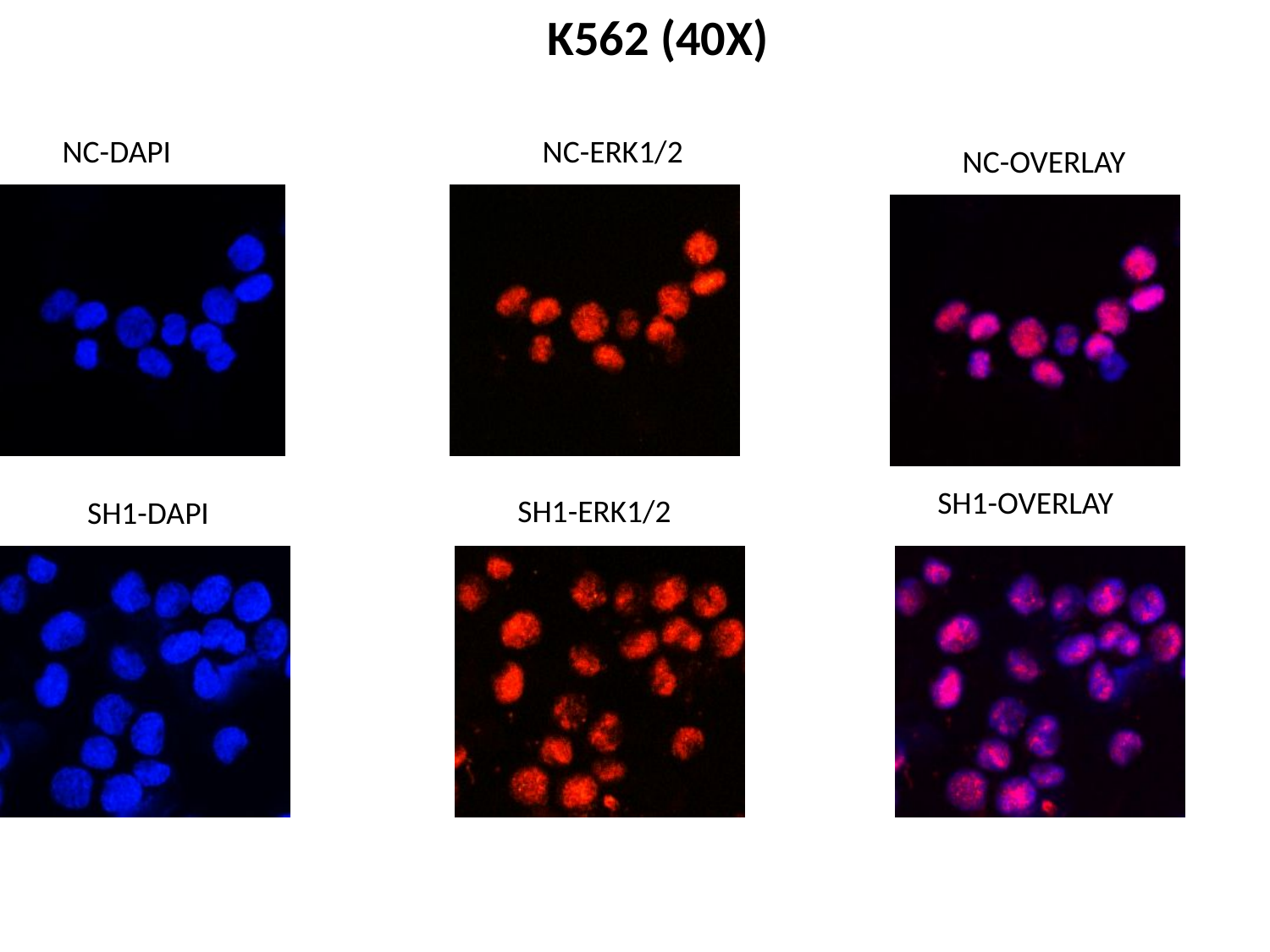

K562 (40X)
NC-DAPI
NC-ERK1/2
NC-OVERLAY
SH1-OVERLAY
SH1-ERK1/2
SH1-DAPI

Supplement: Supplementary file 3 [file DataSheet_3.zip › Figure 6 original data/6E.pptx]

## Slide 1
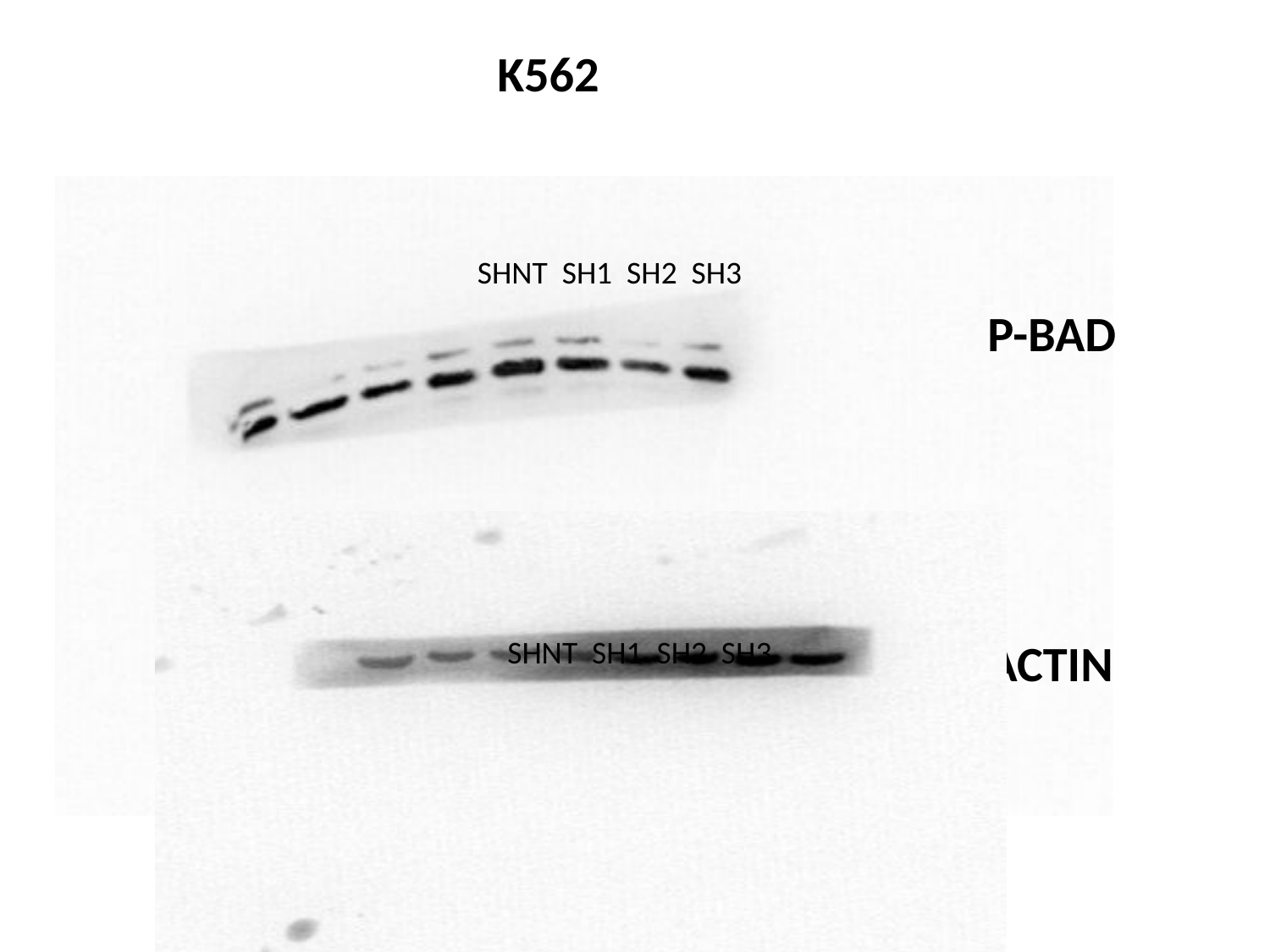

K562
SHNT SH1 SH2 SH3
P-BAD
SHNT SH1 SH2 SH3
ACTIN

Supplement: Supplementary file 3 [file DataSheet_3.zip › Figure 6 original data/6F.pptx]

## Slide 1
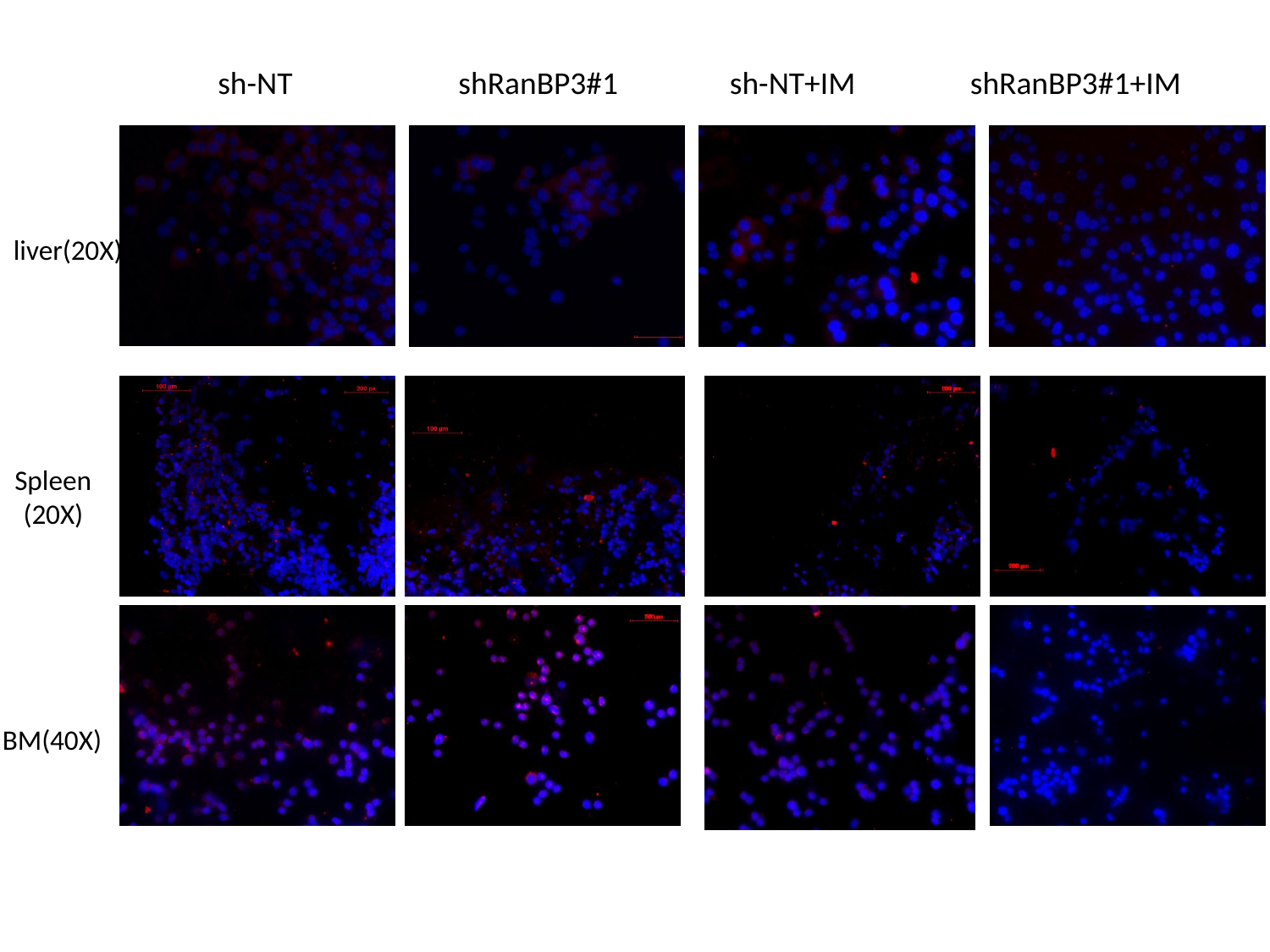

sh-NT
shRanBP3#1
sh-NT+IM
shRanBP3#1+IM
liver(20X)
Spleen
(20X)
BM(40X)

Supplement: Supplementary file 3 [file DataSheet_3.zip › Supplement data/S2.pptx]

## Slide 1
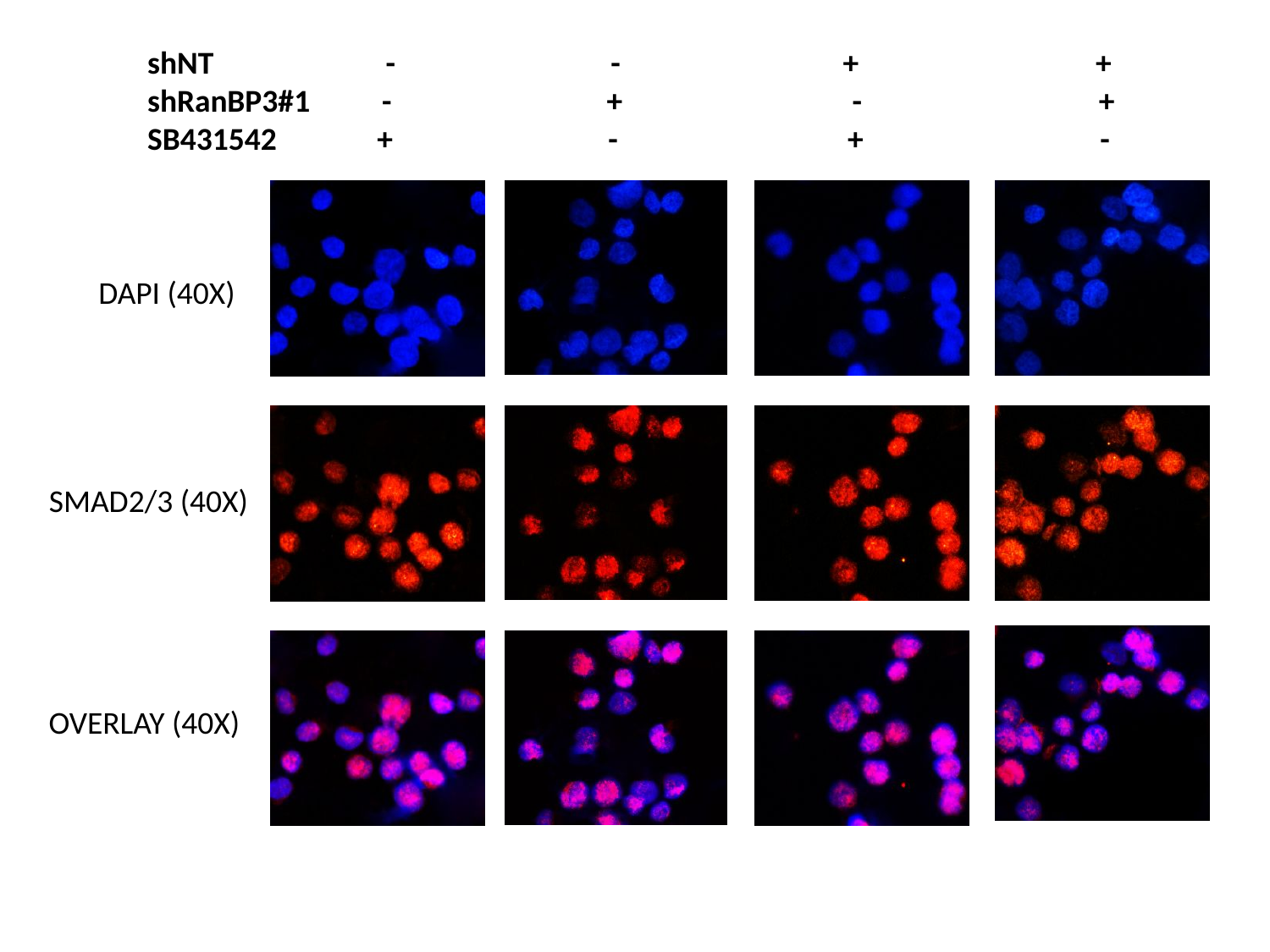

shNT - - + +
shRanBP3#1 - + - +
SB431542 + - + -
DAPI (40X)
SMAD2/3 (40X)
OVERLAY (40X)

Supplement: Supplementary file 3 [file DataSheet_3.zip › Supplement data/S3.pptx]
