# Supplementary material for: RanBP3 Regulates Proliferation, Apoptosis and Chemosensitivity of Chronic Myeloid Leukemia Cells via Mediating SMAD2/3 and ERK1/2 Nuclear Transport
Source: Front Oncol. 2021 Aug 24;11:698410. doi: 10.3389/fonc.2021.698410 (PMC8421687; doi:10.3389/fonc.2021.698410)
Supplement: Supplementary file 3 [file DataSheet_3.zip › Figure 4 original data/4DE.pdf]

SHNT-K562/G01 +IM

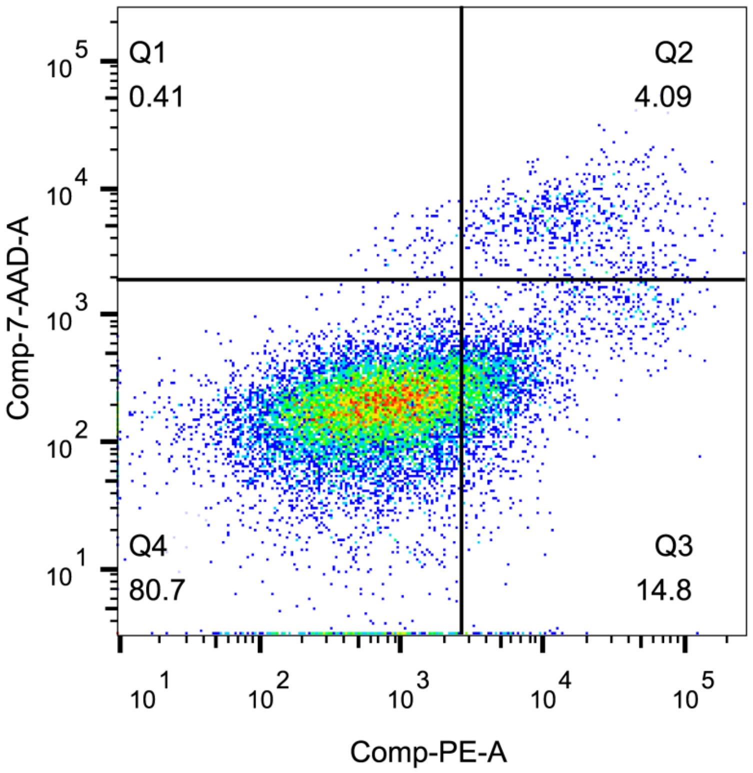

180716_Tube_005_021.fcs
Comp-GFP-A, SSC-A 子细胞群
16887

SH1-K562/G01 +IM

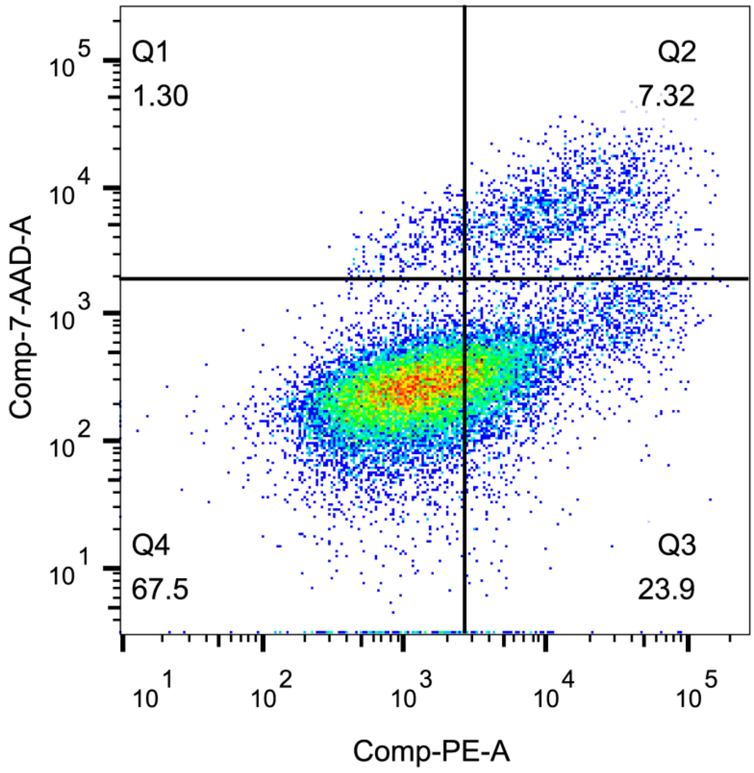

180716_Tube_006_022.fcs
Comp-GFP-A, SSC-A 子细胞群
21614

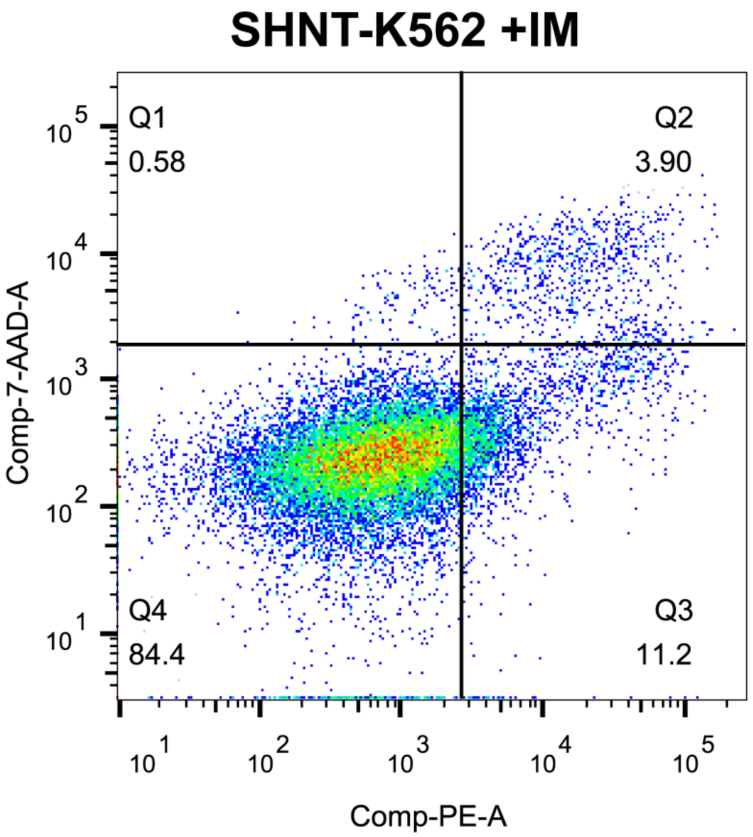

180716_Tube_008_024.fcs

Comp-GFP-A, SSC-A 子细胞群

21207

SH1-K562 +IM

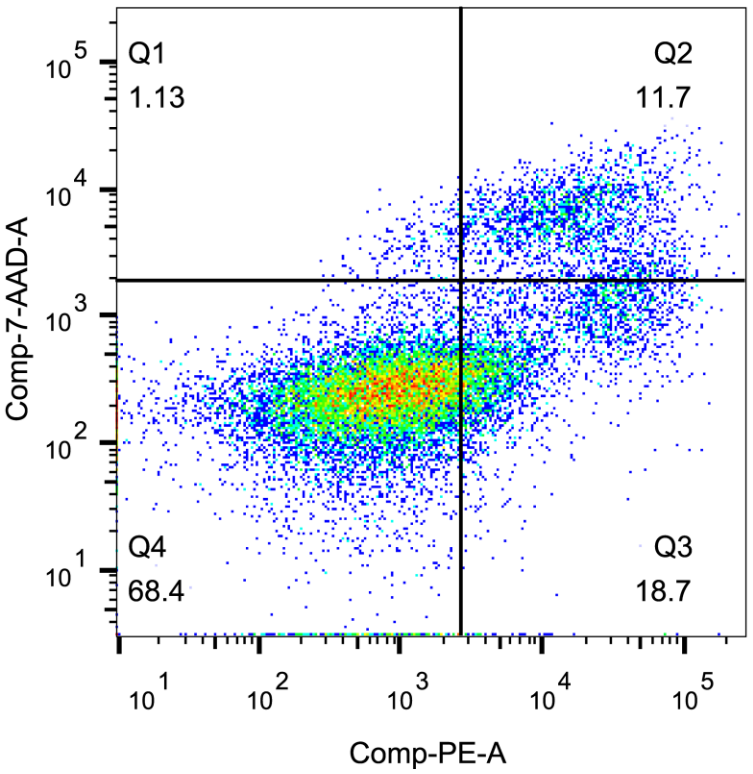

180716_Tube_007_023.fcs

Comp-GFP-A, SSC-A 子细胞群

18824
