# Supplementary material for: RanBP3 Regulates Proliferation, Apoptosis and Chemosensitivity of Chronic Myeloid Leukemia Cells via Mediating SMAD2/3 and ERK1/2 Nuclear Transport
Source: Front Oncol. 2021 Aug 24;11:698410. doi: 10.3389/fonc.2021.698410 (PMC8421687; doi:10.3389/fonc.2021.698410)
Supplement: Supplementary file 3 [file DataSheet_3.zip › Figure 6 original data/6A.pptx]

## Slide 1
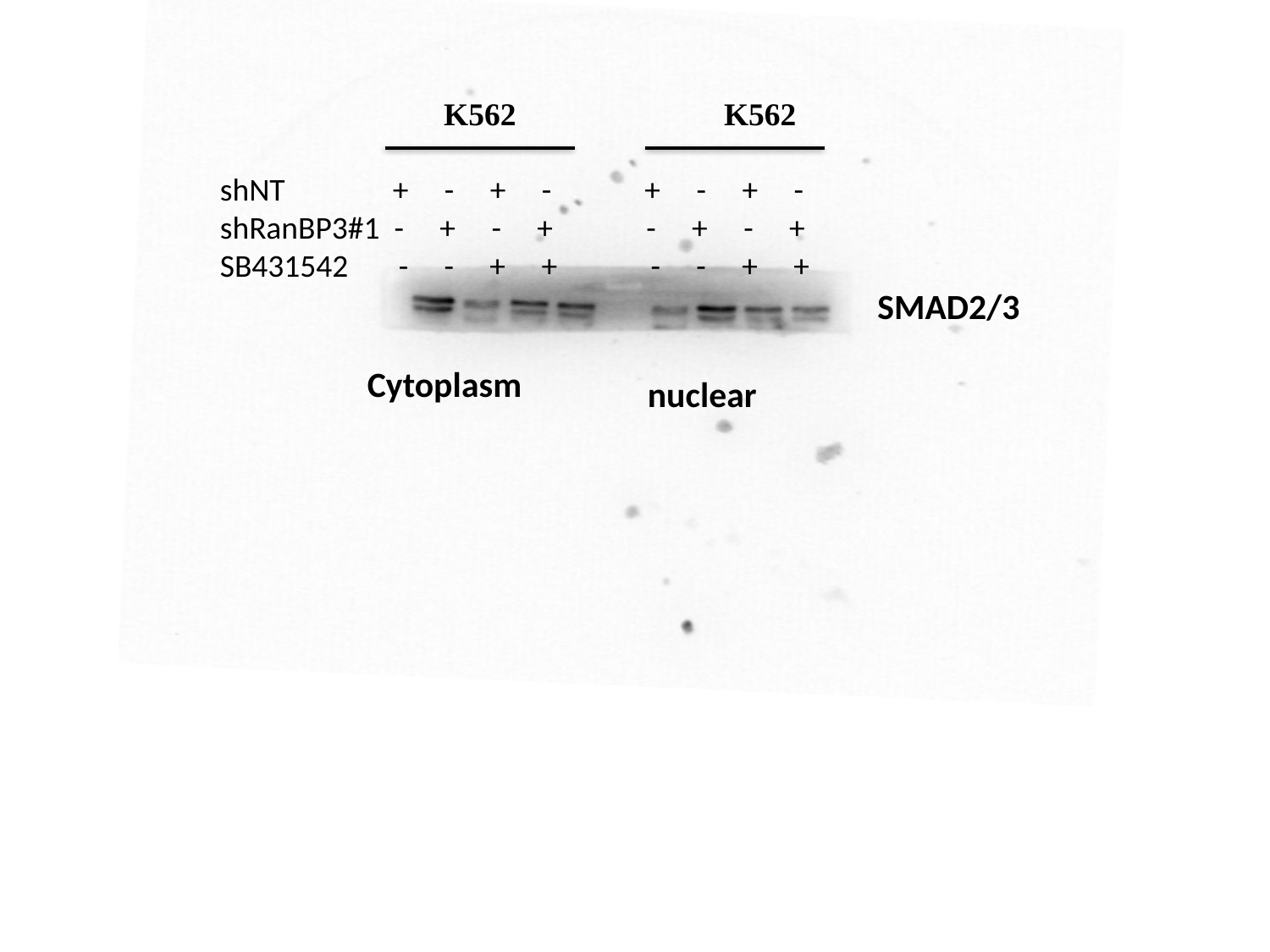

K562
K562
shNT + - + - + - + -
shRanBP3#1 - + - + - + - +
SB431542 - - + + - - + +
SMAD2/3
Cytoplasm
nuclear

## Slide 2
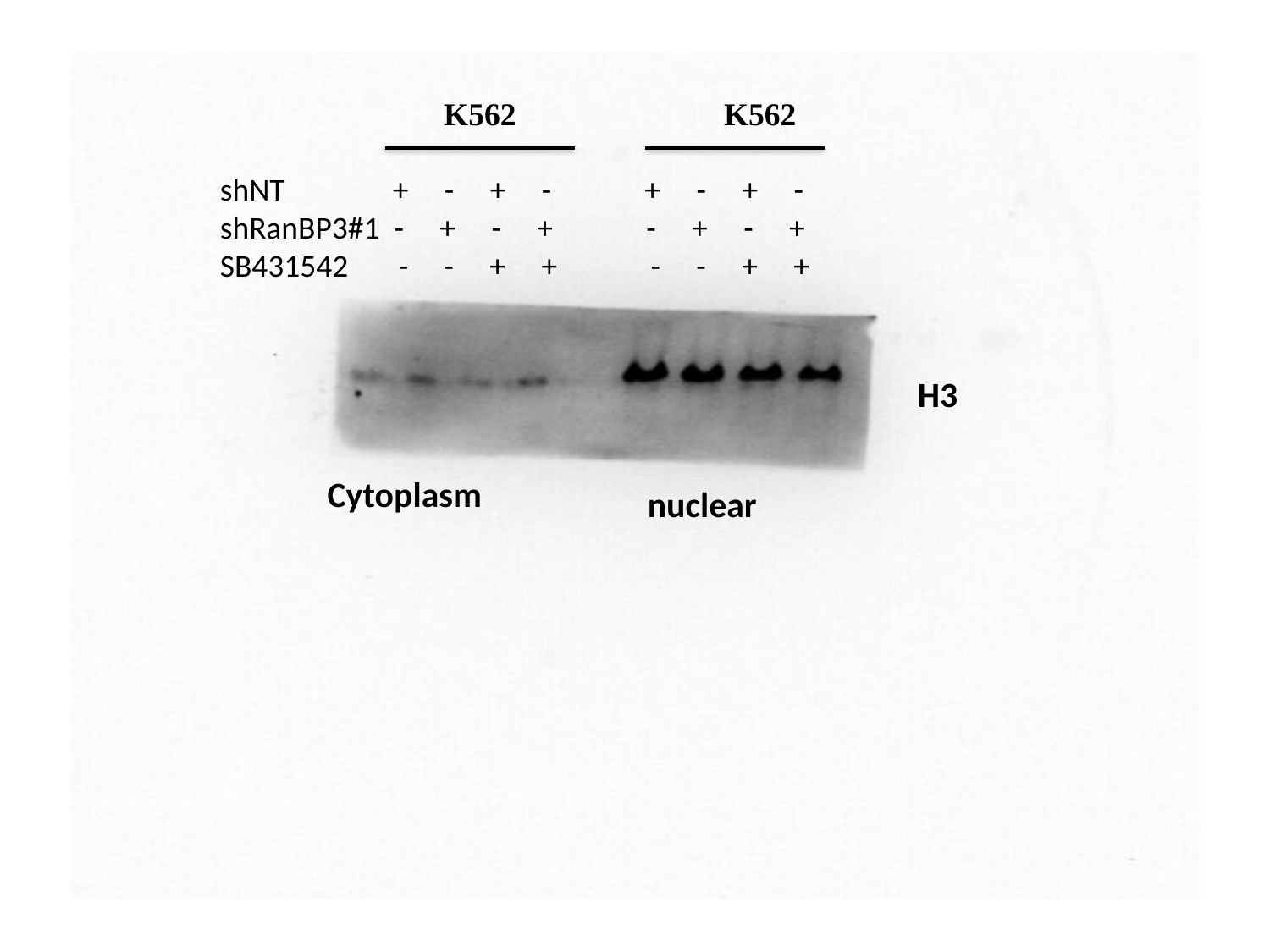

K562
K562
shNT + - + - + - + -
shRanBP3#1 - + - + - + - +
SB431542 - - + + - - + +
H3
Cytoplasm
nuclear

## Slide 3
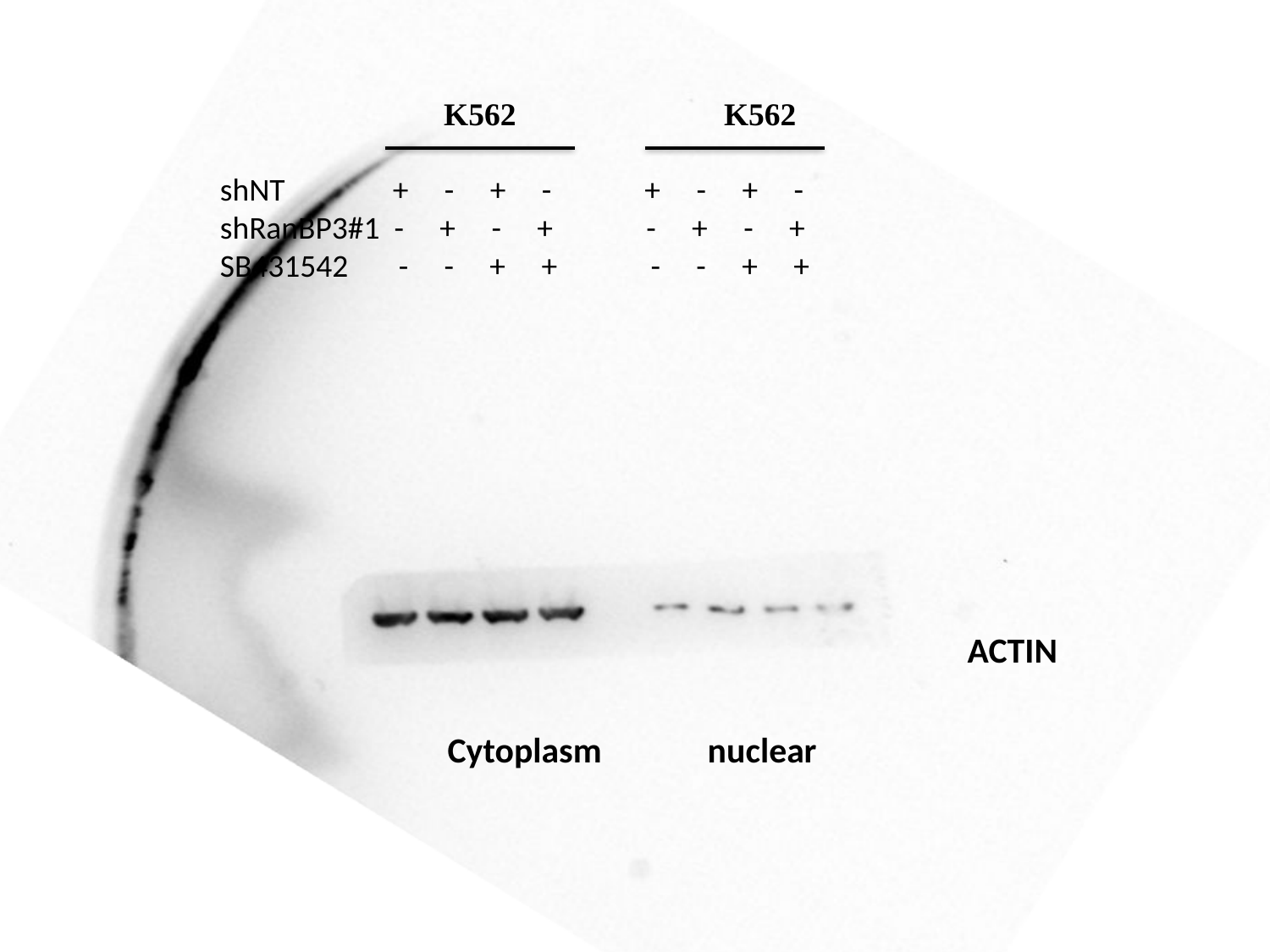

K562
K562
shNT + - + - + - + -
shRanBP3#1 - + - + - + - +
SB431542 - - + + - - + +
ACTIN
Cytoplasm
nuclear
