# Supplementary material for: RanBP3 Regulates Proliferation, Apoptosis and Chemosensitivity of Chronic Myeloid Leukemia Cells via Mediating SMAD2/3 and ERK1/2 Nuclear Transport
Source: Front Oncol. 2021 Aug 24;11:698410. doi: 10.3389/fonc.2021.698410 (PMC8421687; doi:10.3389/fonc.2021.698410)
Supplement: Supplementary file 3 [file DataSheet_3.zip › Figure 6 original data/6B.pptx]

## Slide 1
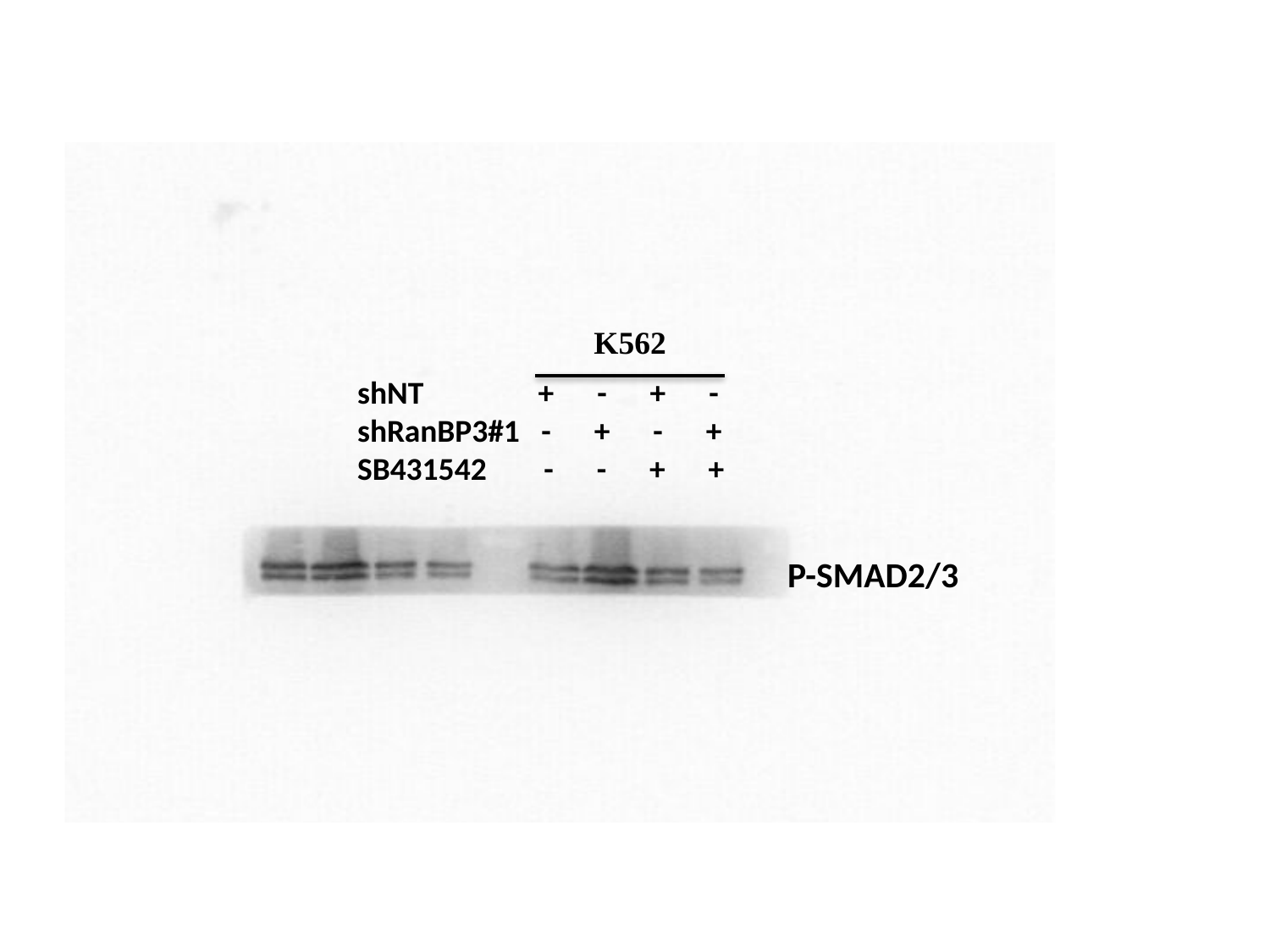

K562
shNT + - + -
shRanBP3#1 - + - +
SB431542 - - + +
P-SMAD2/3

## Slide 2
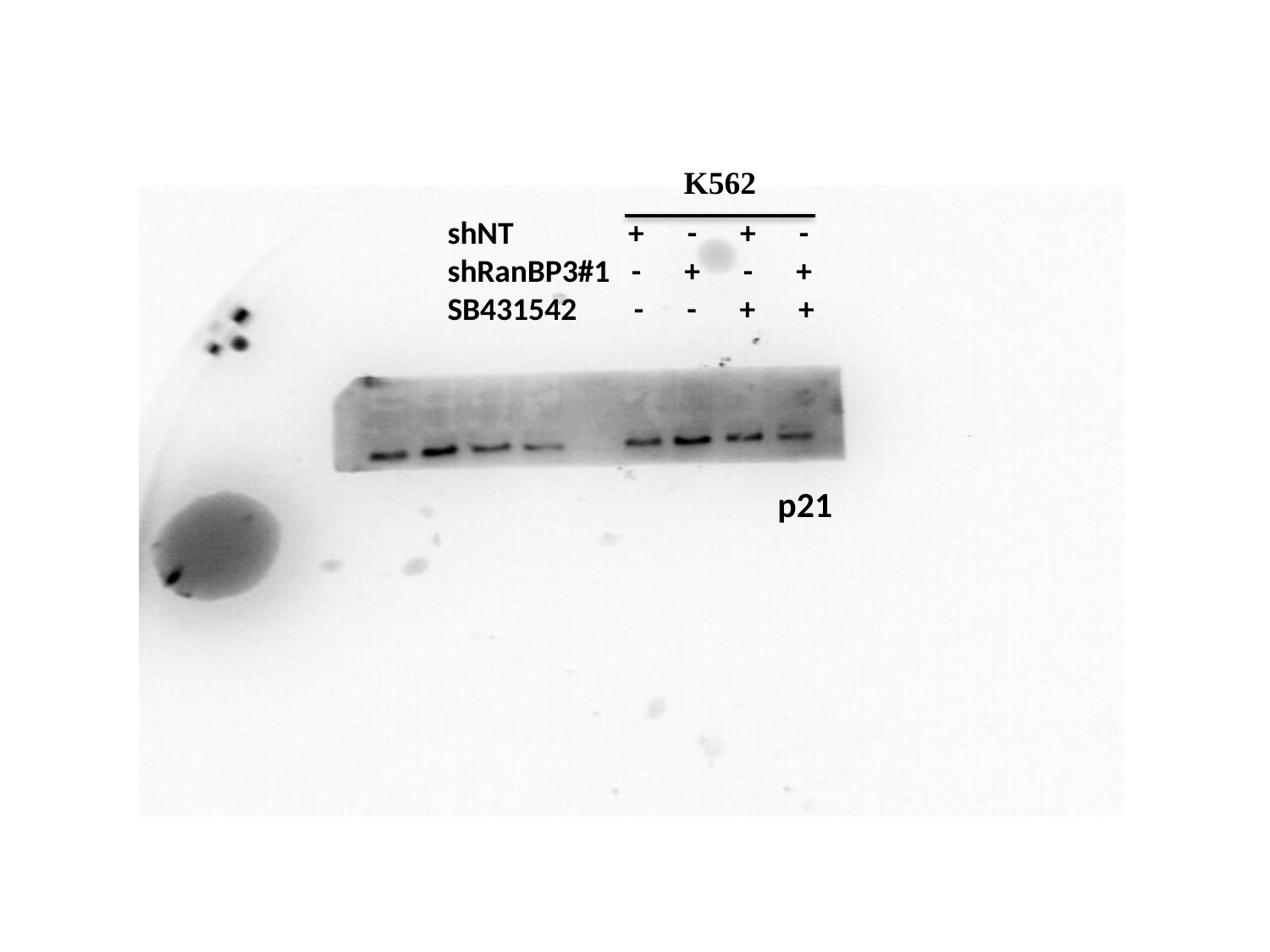

K562
shNT + - + -
shRanBP3#1 - + - +
SB431542 - - + +
p21

## Slide 3
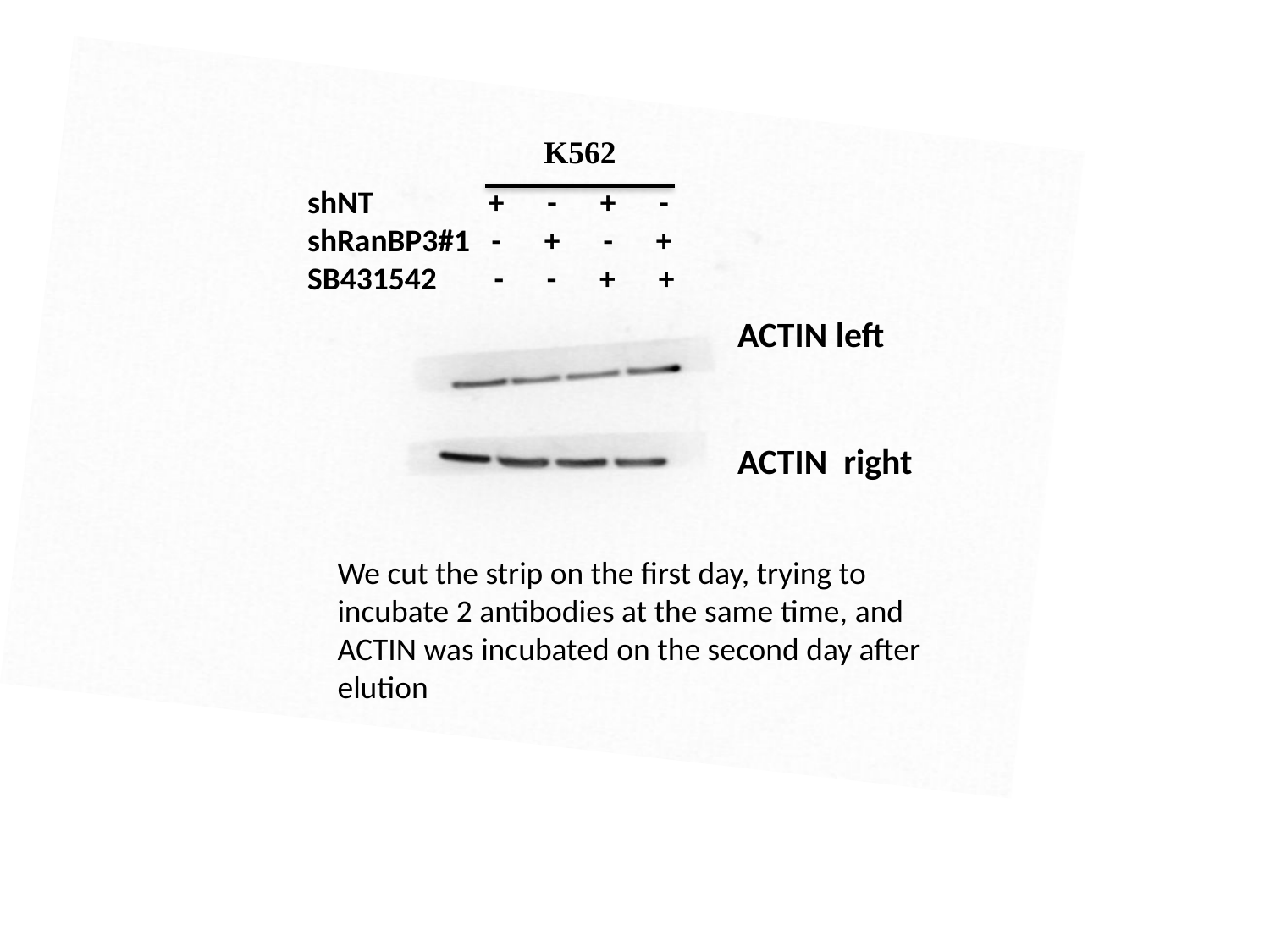

K562
shNT + - + -
shRanBP3#1 - + - +
SB431542 - - + +
ACTIN left
ACTIN right
We cut the strip on the first day, trying to incubate 2 antibodies at the same time, and ACTIN was incubated on the second day after elution
