# Supplementary material for: RanBP3 Regulates Proliferation, Apoptosis and Chemosensitivity of Chronic Myeloid Leukemia Cells via Mediating SMAD2/3 and ERK1/2 Nuclear Transport
Source: Front Oncol. 2021 Aug 24;11:698410. doi: 10.3389/fonc.2021.698410 (PMC8421687; doi:10.3389/fonc.2021.698410)
Supplement: Supplementary file 3 [file DataSheet_3.zip › Figure 6 original data/6G.pptx]

## Slide 1
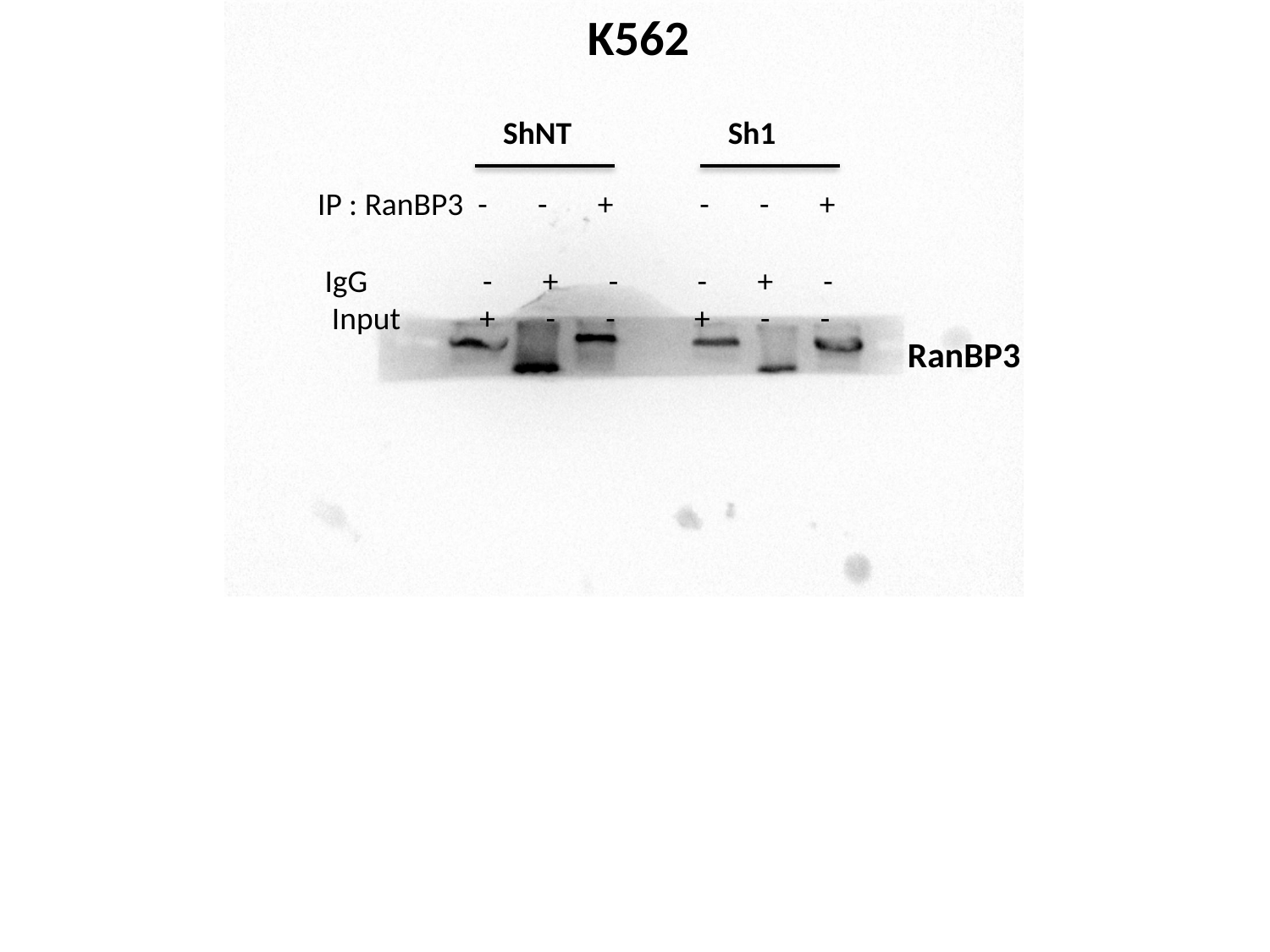

K562
ShNT
Sh1
IP : RanBP3 - - + - - +
 IgG - + - - + -
 Input + - - + - -
RanBP3

## Slide 2
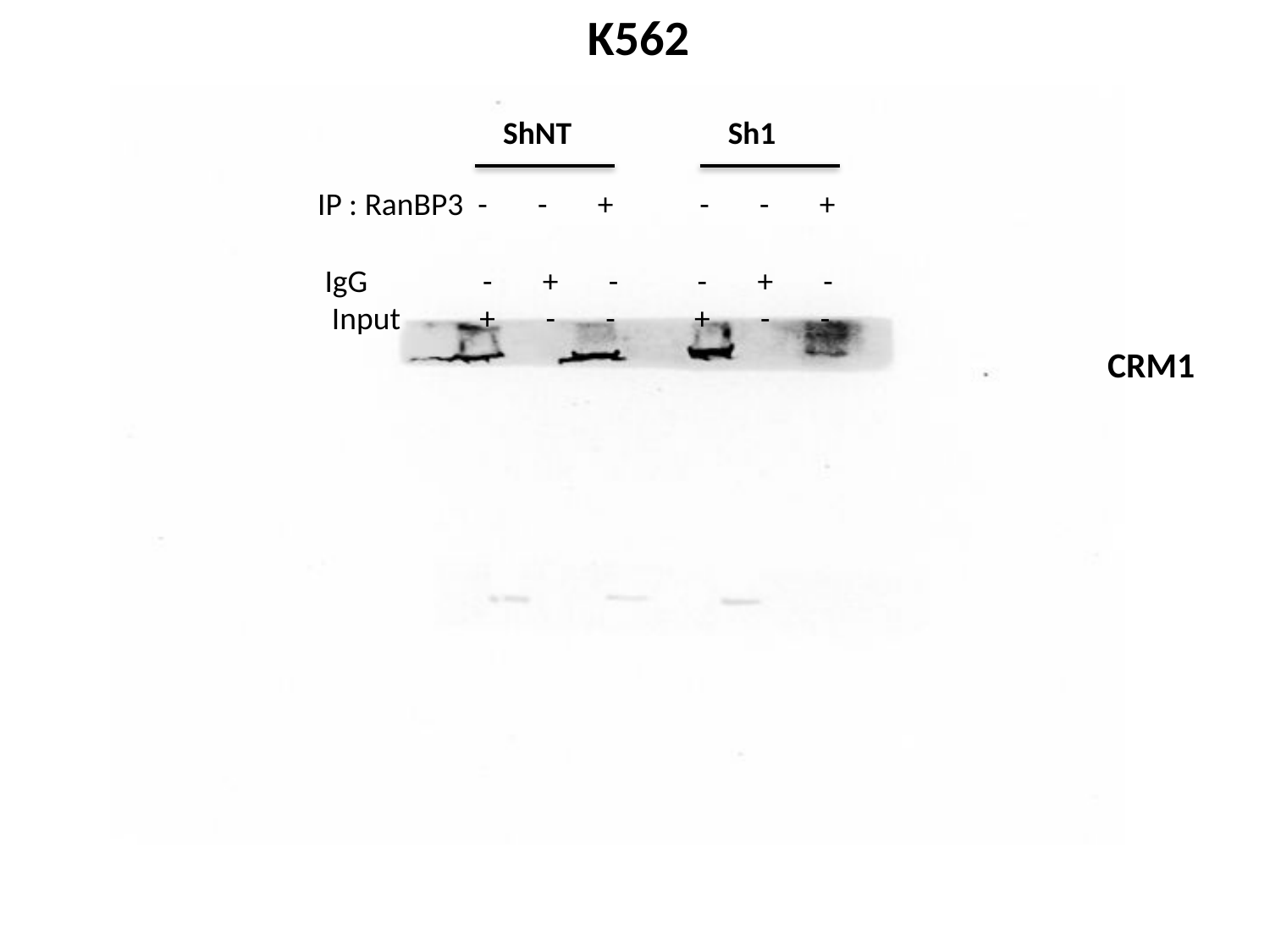

K562
ShNT
Sh1
IP : RanBP3 - - + - - +
 IgG - + - - + -
 Input + - - + - -
CRM1

## Slide 3
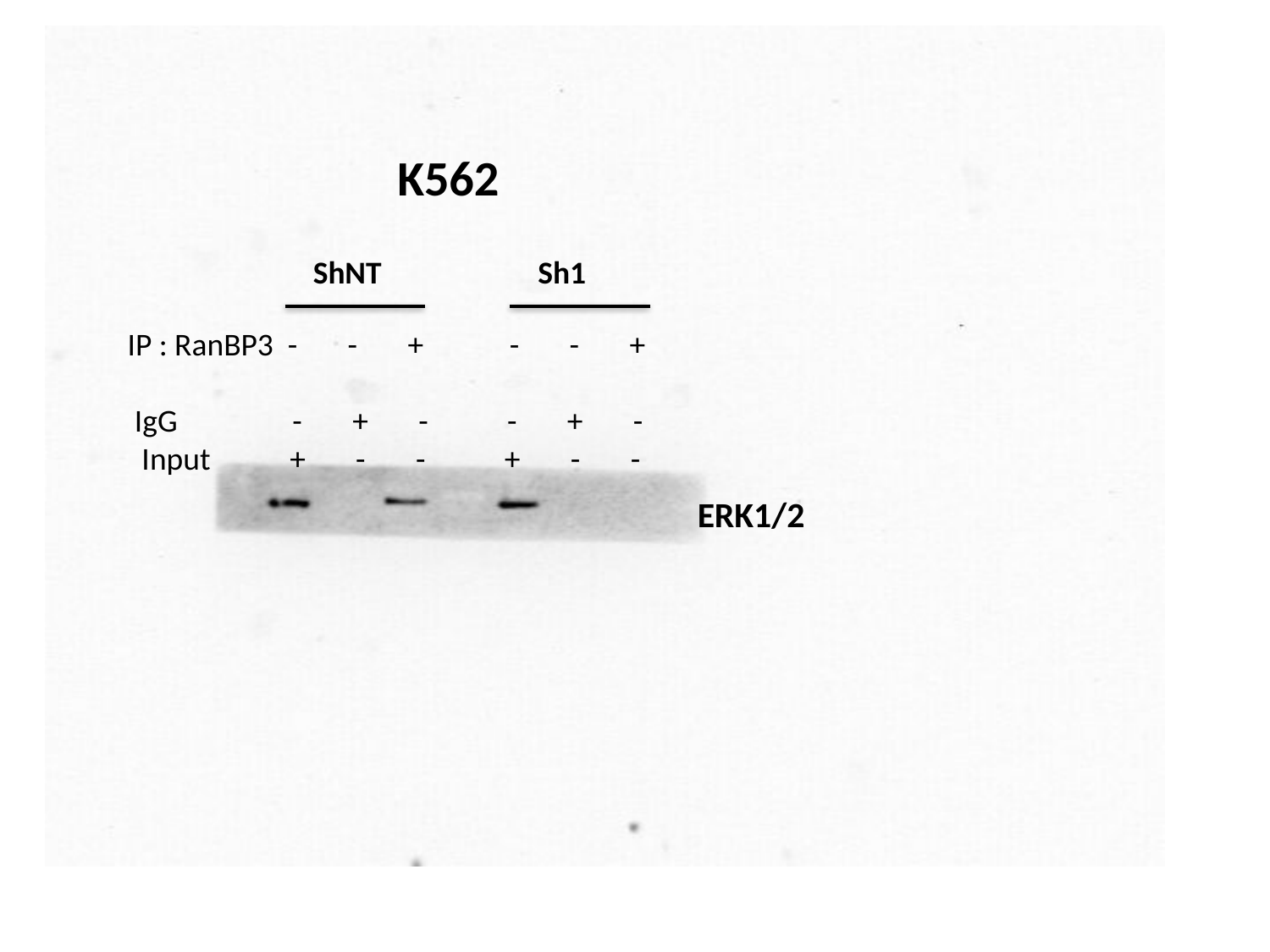

K562
ShNT
Sh1
IP : RanBP3 - - + - - +
 IgG - + - - + -
 Input + - - + - -
ERK1/2

## Slide 4
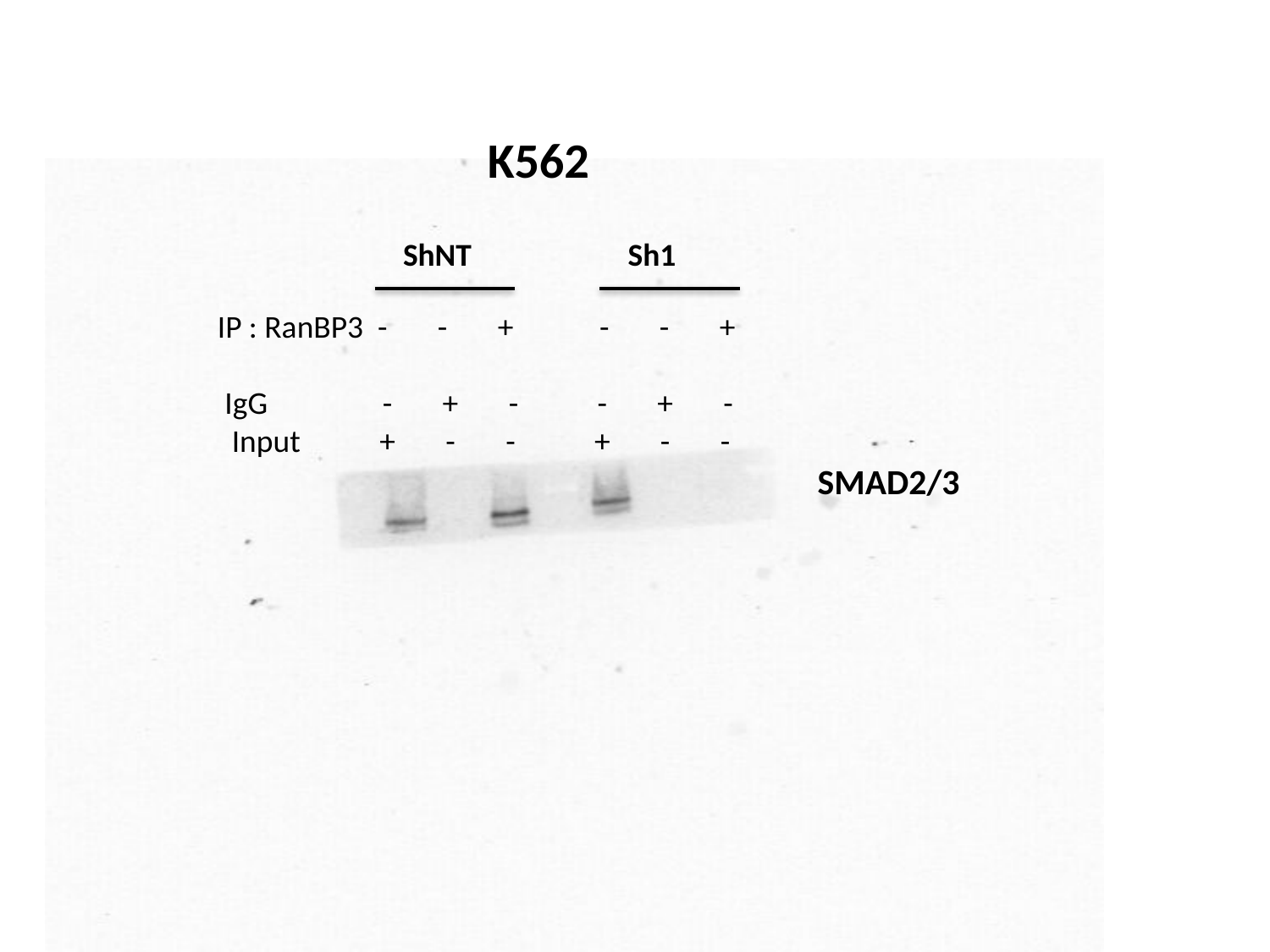

K562
ShNT
Sh1
IP : RanBP3 - - + - - +
 IgG - + - - + -
 Input + - - + - -
SMAD2/3
