# Supplementary material for: RanBP3 Regulates Proliferation, Apoptosis and Chemosensitivity of Chronic Myeloid Leukemia Cells via Mediating SMAD2/3 and ERK1/2 Nuclear Transport
Source: Front Oncol. 2021 Aug 24;11:698410. doi: 10.3389/fonc.2021.698410 (PMC8421687; doi:10.3389/fonc.2021.698410)
Supplement: Supplementary file 3 [file DataSheet_3.zip › Supplement data/S1B.pdf]

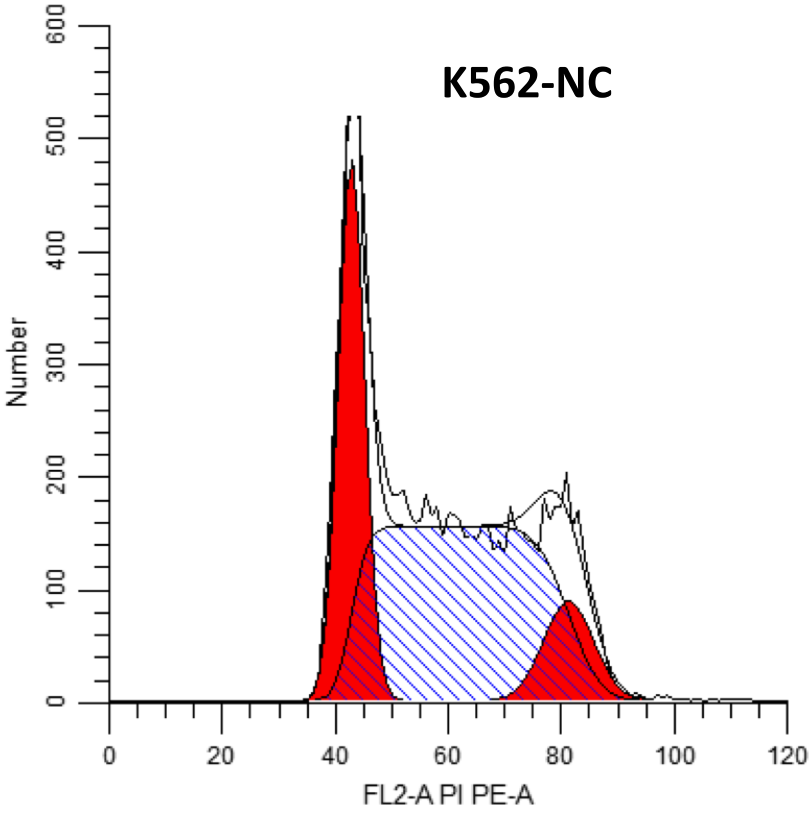

File analyzed: 1.fcs
Date analyzed: 28-Aug-2019
Model: 1nn0n_DSD
Analysis type: Manual analysis
Auto Linearity: No

Ploidy Mode: First cycle is diploid

Diploid: 100.00 %
    Dip G1: 28.08 % at 42.80
    Dip G2: 10.04 % at 81.33
    Dip S: 61.88 %   G2/G1: 1.90
    %CV: 5.46

Total S-Phase: 61.88 %
Total B.A.D.: 0.00 %   no debris no aggs

Debris: %
Aggregates: %
Modeled events: 9768
All cycle events: 9768
Cycle events per channel: 247
RCS: 2.074

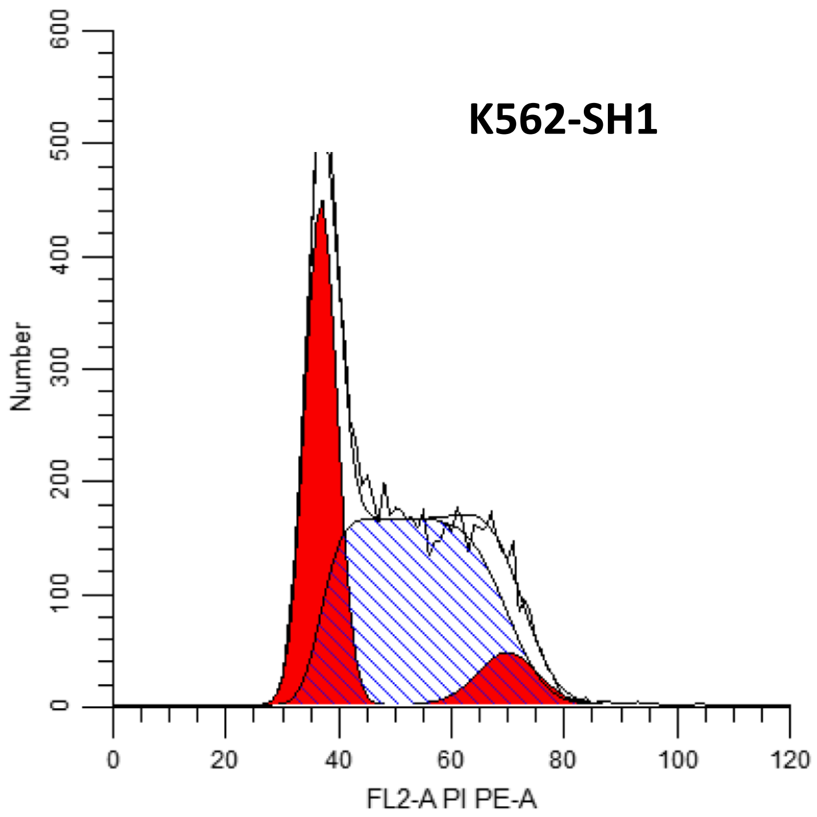

File analyzed: 2.fcs
Date analyzed: 28-Aug-2019
Model: 1nn0n_DSD
Analysis type: Manual analysis
Auto Linearity: No

Ploidy Mode: First cycle is diploid

Diploid: 100.00 %
Dip G1: 34.48 % at 36.78
Dip G2: 6.73 % at 69.88
Dip S: 58.79 % G2/G1: 1.90
%CV: 7.69

Total S-Phase: 58.79 %
Total B.A.D.: 0.00 % no debris no aggs

Debris: %
Aggregates: %
Modeled events: 9286
All cycle events: 9286
Cycle events per channel: 272
RCS: 1.143

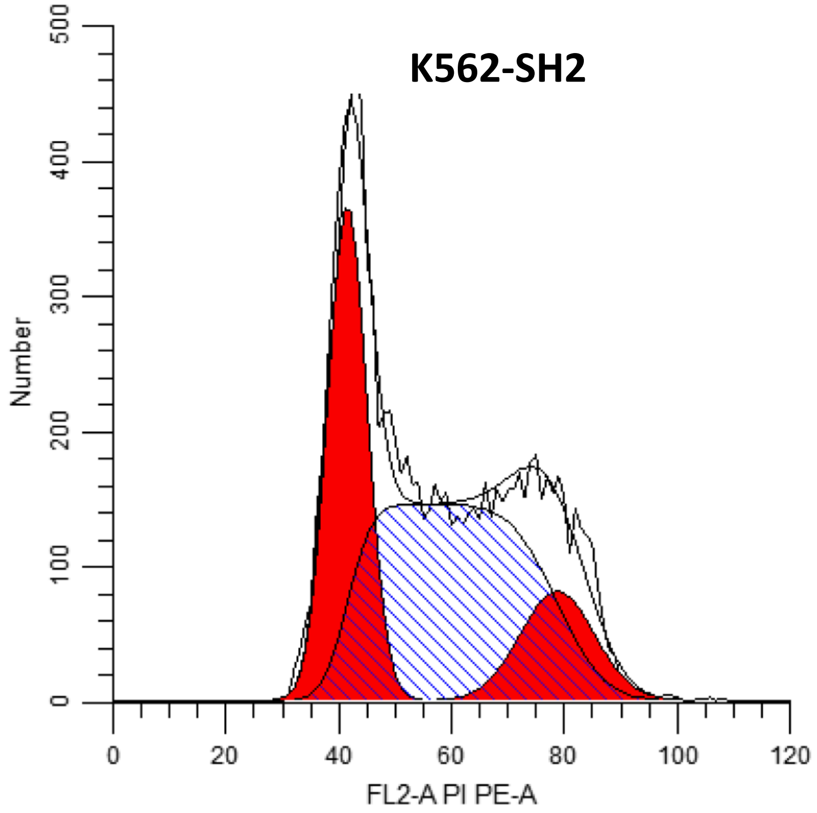

File analyzed: 3.fcs
Date analyzed: 28-Aug-2019
Model: 1nn0n_DSD
Analysis type: Manual analysis
Auto Linearity: No

Ploidy Mode: First cycle is diploid

Diploid: 100.00 %
Dip G1: 32.27 % at 41.45
Dip G2: 13.44 % at 78.76
Dip S: 54.29 % G2/G1: 1.90
%CV: 8.42

Total S-Phase: 54.29 %
Total B.A.D.: 0.00 % no debris no aggs

Debris: %
Aggregates: %
Modeled events: 9967
All cycle events: 9967
Cycle events per channel: 260
RCS: 2.282

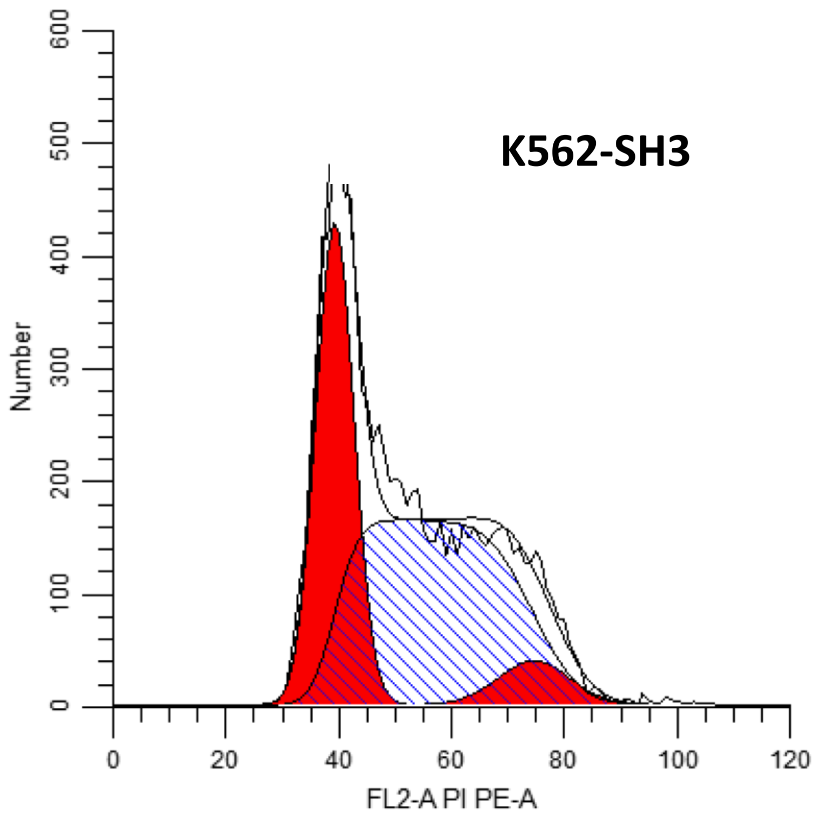

File analyzed: 4.fcs
Date analyzed: 28-Aug-2019
Model: 1nn0n_DSD
Analysis type: Manual analysis
Auto Linearity: No

Ploidy Mode: First cycle is diploid

Diploid: 100.00 %
Dip G1: 36.24 % at 39.23
Dip G2: 6.22 % at 74.54
Dip S: 57.54 % G2/G1: 1.90
%CV: 8.57

Total S-Phase: 57.54 %
Total B.A.D.: 0.00 % no debris no aggs

Debris: %
Aggregates: %
Modeled events: 10046
All cycle events: 10046
Cycle events per channel: 277
RCS: 2.175

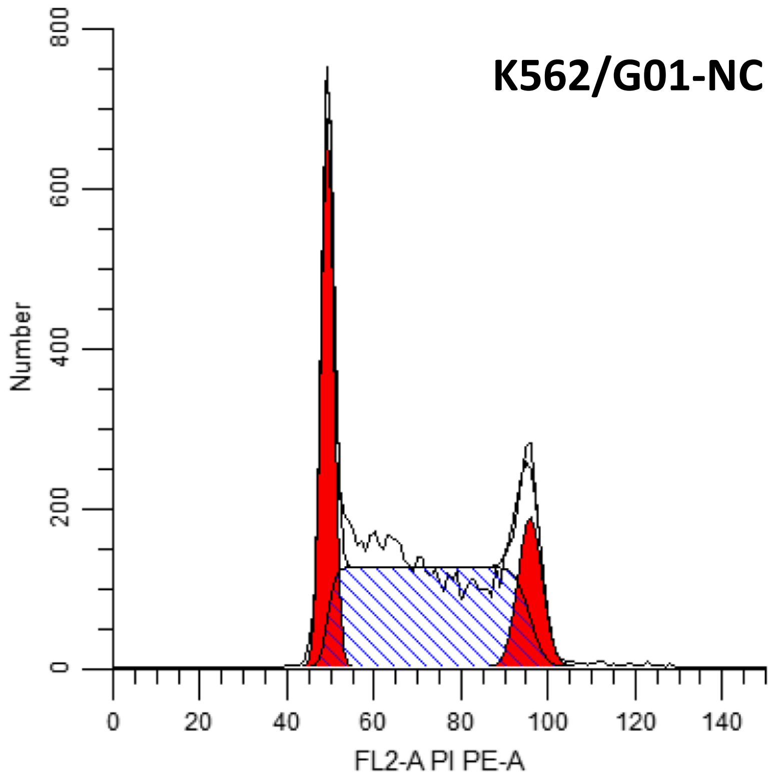

File analyzed: 1.fcs
Date analyzed: 15-Jun-2020
Model: 1nn0n_DSD
Analysis type: Manual analysis
Auto Linearity: No

Ploidy Mode: First cycle is diploid

Diploid: 100.00 %
Dip G1: 25.91 % at 49.17
Dip G2: 13.51 % at 95.88
Dip S: 60.58 % G2/G1: 1.95
%CV: 2.85

Total S-Phase: 60.58 %
Total B.A.D.: 0.00 % no debris no aggs

Debris: %
Aggregates: %
Modeled events: 9572
All cycle events: 9572
Cycle events per channel: 201
RCS: 2.390

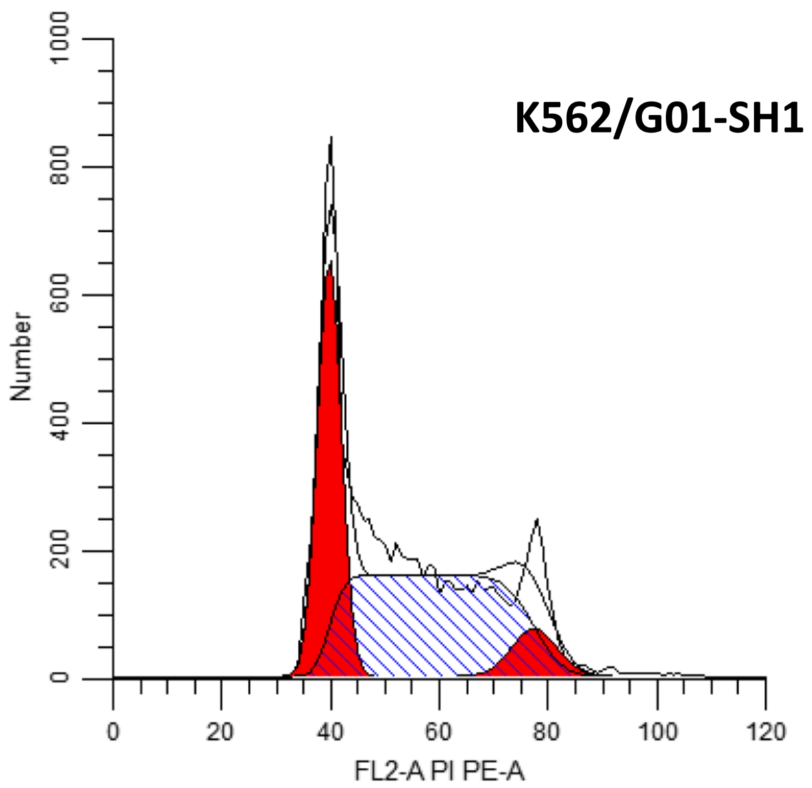

File analyzed: 2.fcs

Date analyzed: 15-Jun-2020

Model: 1nn0n_DSD

Analysis type: Manual analysis

Auto Linearity: No

Ploidy Mode: First cycle is diploid

Diploid: 100.00 %

Dip G1: 34.85 % at 39.70

Dip G2: 7.67 % at 77.41

Dip S: 57.48 %   G2/G1: 1.95

%CV: 5.47

Total S-Phase: 57.48 %

Total B.A.D.: 0.00 %   no debris no aggs

Debris: %

Aggregates: %

Modeled events: 10352

All cycle events: 10352

Cycle events per channel: 267

RCS: 6.243

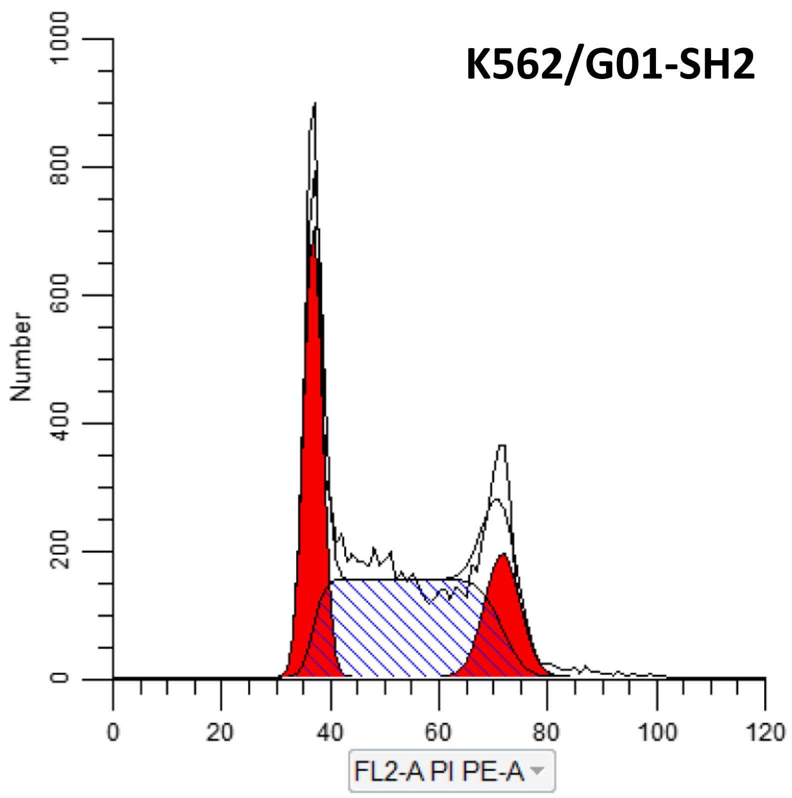

File analyzed: 3.fcs

Date analyzed: 15-Jun-2020

Model: 1nn0n_DSD

Analysis type: Manual analysis

Auto Linearity: No

Ploidy Mode: First cycle is diploid

Diploid: 100.00 %

Dip G1: 30.53 % at 36.73

Dip G2: 16.00 % at 71.62

Dip S: 53.47 %   G2/G1: 1.95

%CV: 4.55

Total S-Phase: 53.47 %

Total B.A.D.: 0.00 %   no debris no aggs

Debris: %

Aggregates: %

Modeled events: 9943

All cycle events: 9943

Cycle events per channel: 277

RCS: 4.892

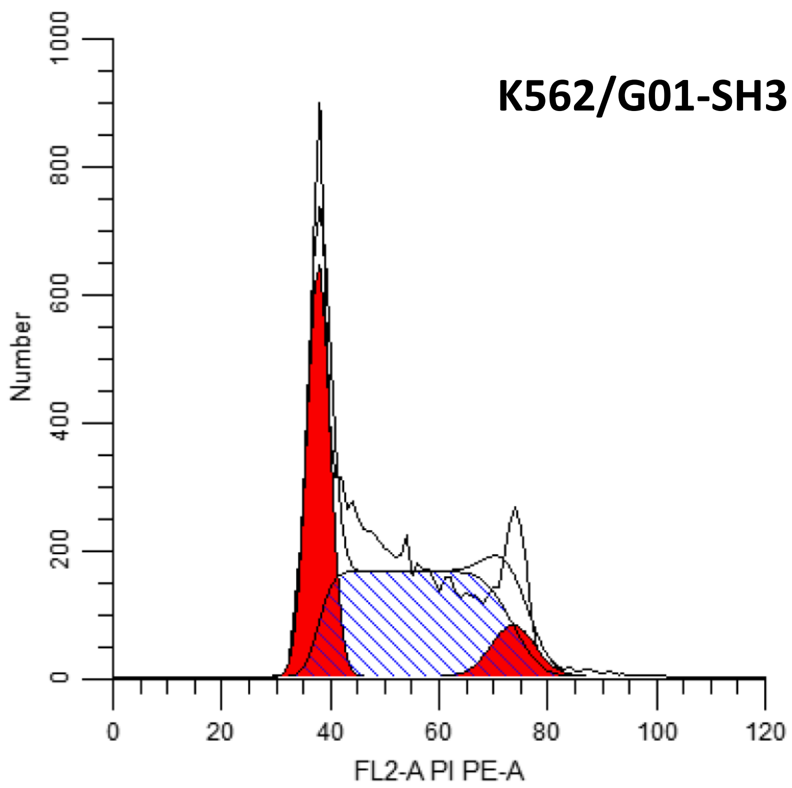

File analyzed: 4.fcs
Date analyzed: 15-Jun-2020
Model: 1nn0n_DSD
Analysis type: Manual analysis
Auto Linearity: No

Ploidy Mode: First cycle is diploid

Diploid: 100.00 %
Dip G1: 33.72 % at 37.73
Dip G2: 8.16 % at 73.57
Dip S: 58.12 % G2/G1: 1.95
%CV: 5.52

Total S-Phase: 58.12 %
Total B.A.D.: 0.00 % no debris no aggs

Debris: %
Aggregates: %
Modeled events: 10157
All cycle events: 10157
Cycle events per channel: 276
RCS: 6.410
