# Supplementary material for: RanBP3 Regulates Proliferation, Apoptosis and Chemosensitivity of Chronic Myeloid Leukemia Cells via Mediating SMAD2/3 and ERK1/2 Nuclear Transport
Source: Front Oncol. 2021 Aug 24;11:698410. doi: 10.3389/fonc.2021.698410 (PMC8421687; doi:10.3389/fonc.2021.698410)
Supplement: Supplementary file 3 [file DataSheet_3.zip › Supplement data/S4.pptx]

## Slide 1
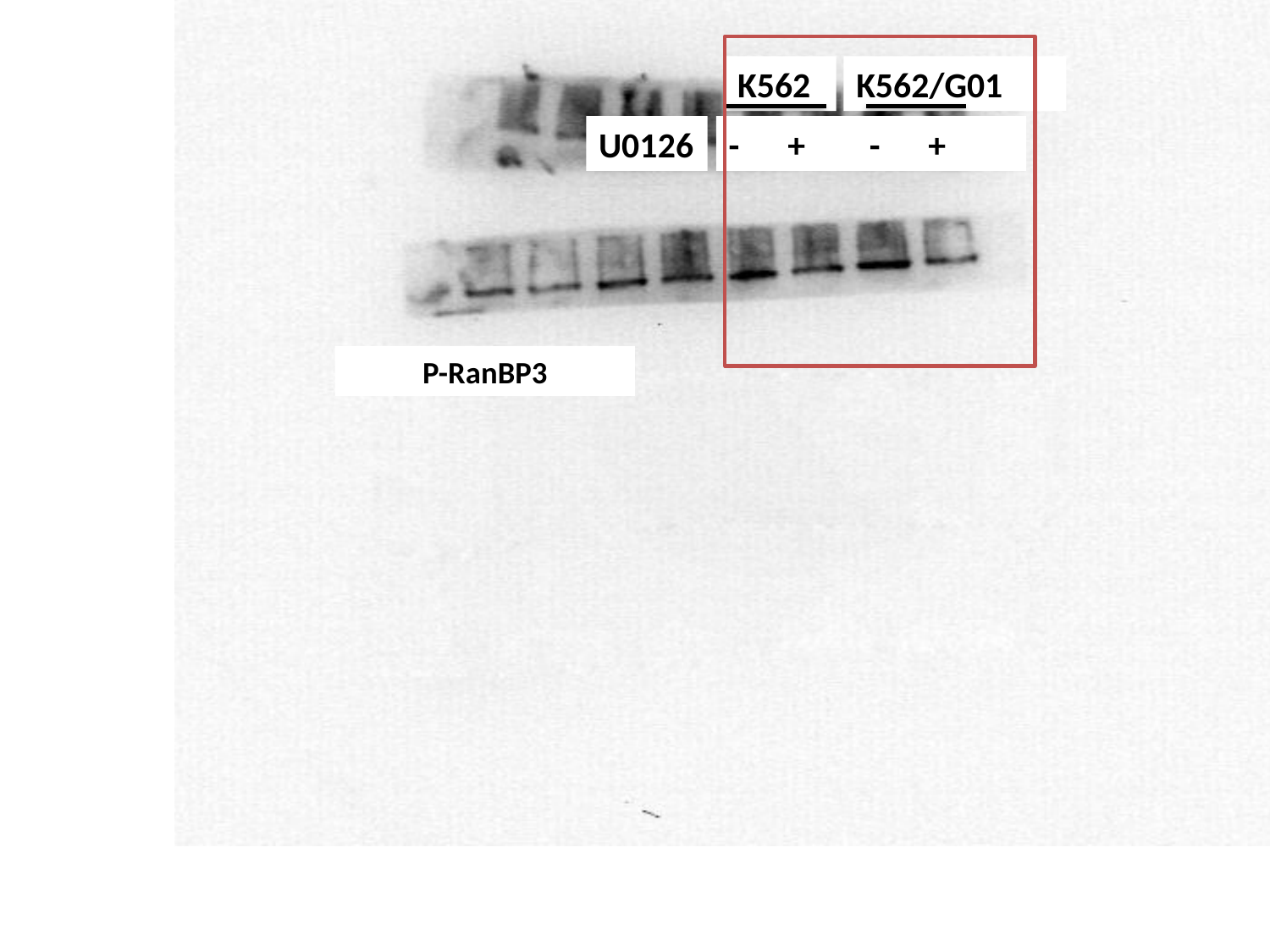

K562
K562/G01
U0126
- + - +
P-RanBP3

## Slide 2
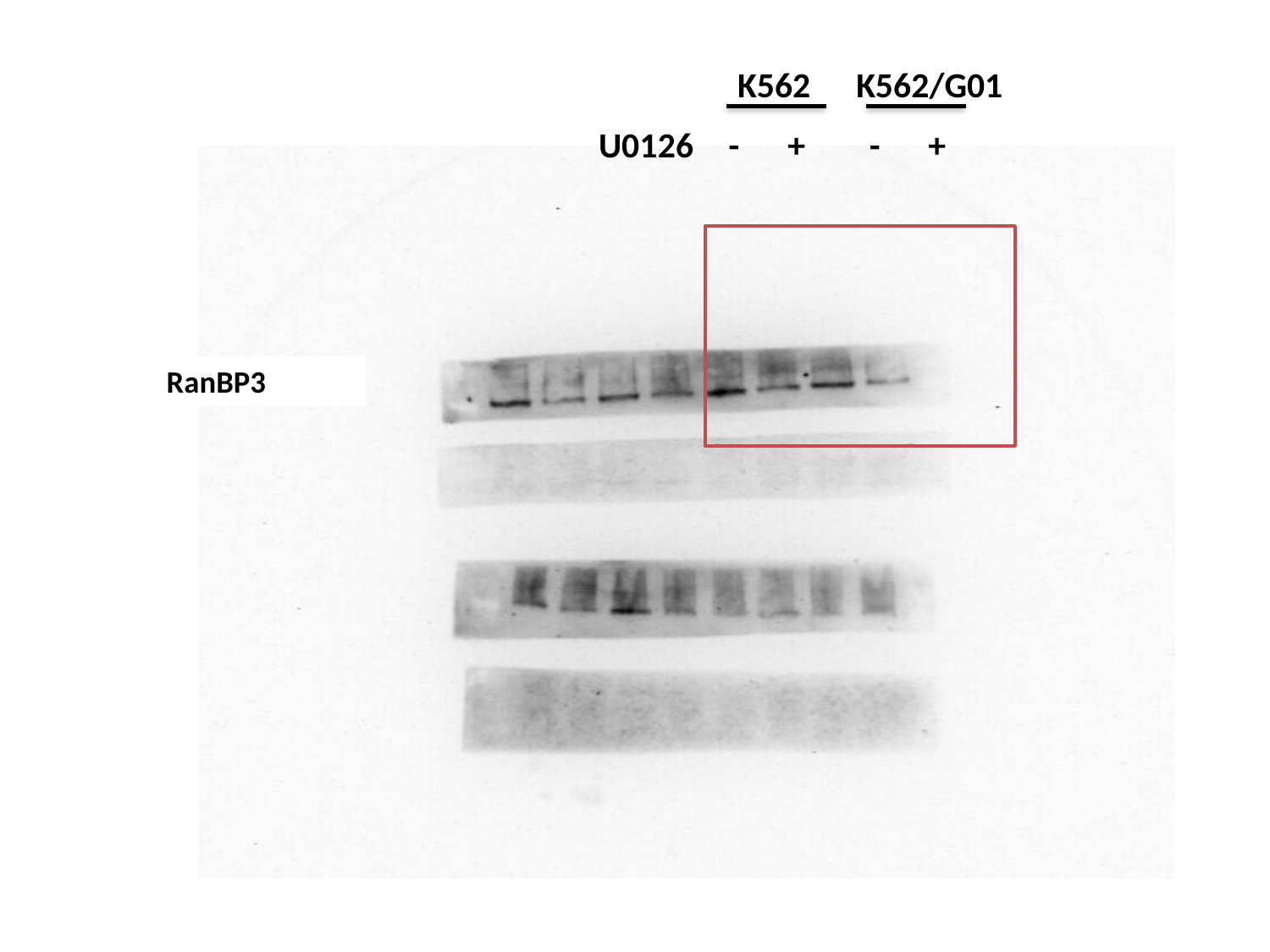

K562
K562/G01
U0126
- + - +
RanBP3

## Slide 3
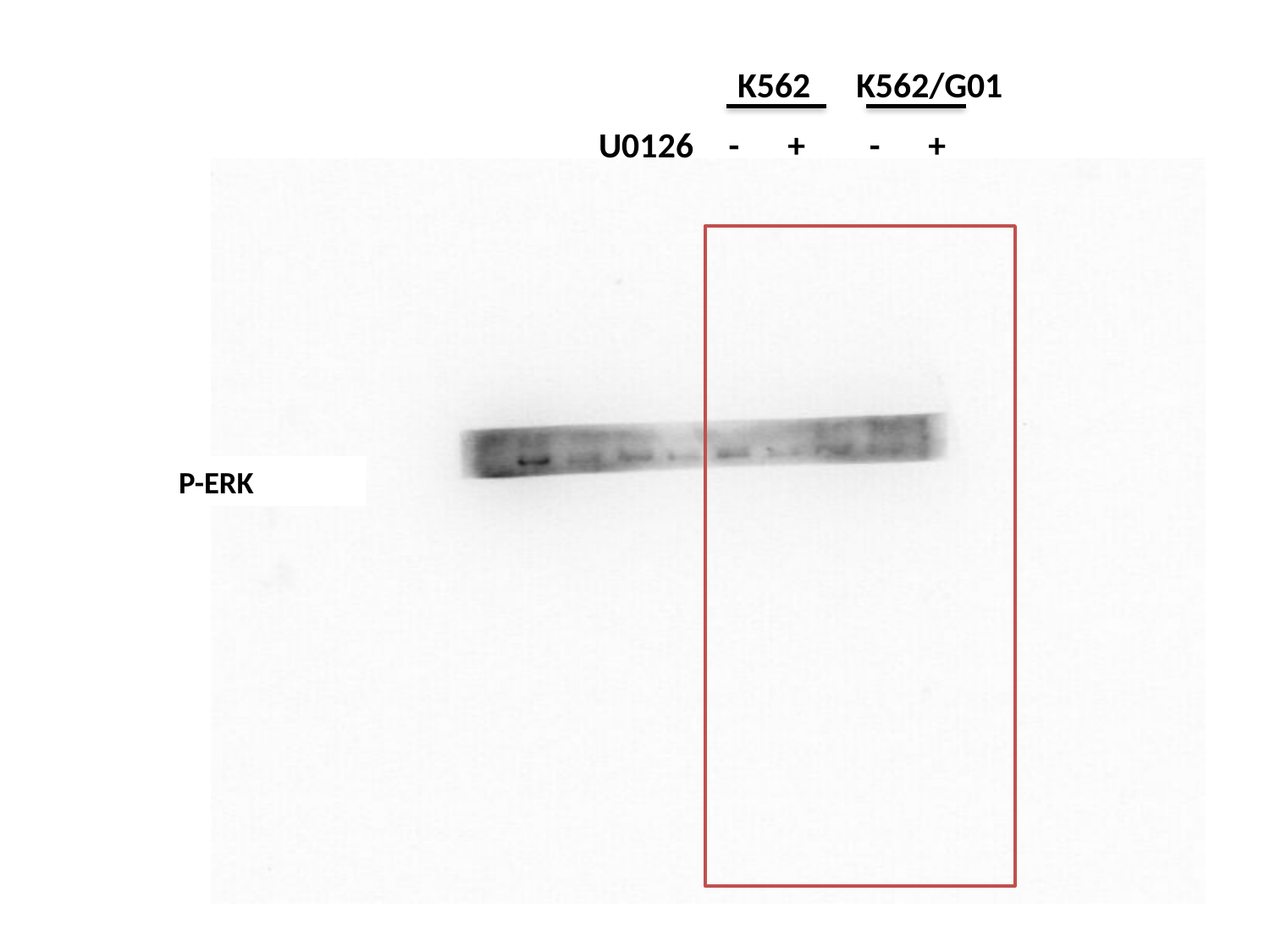

K562
K562/G01
U0126
- + - +
P-ERK

## Slide 4
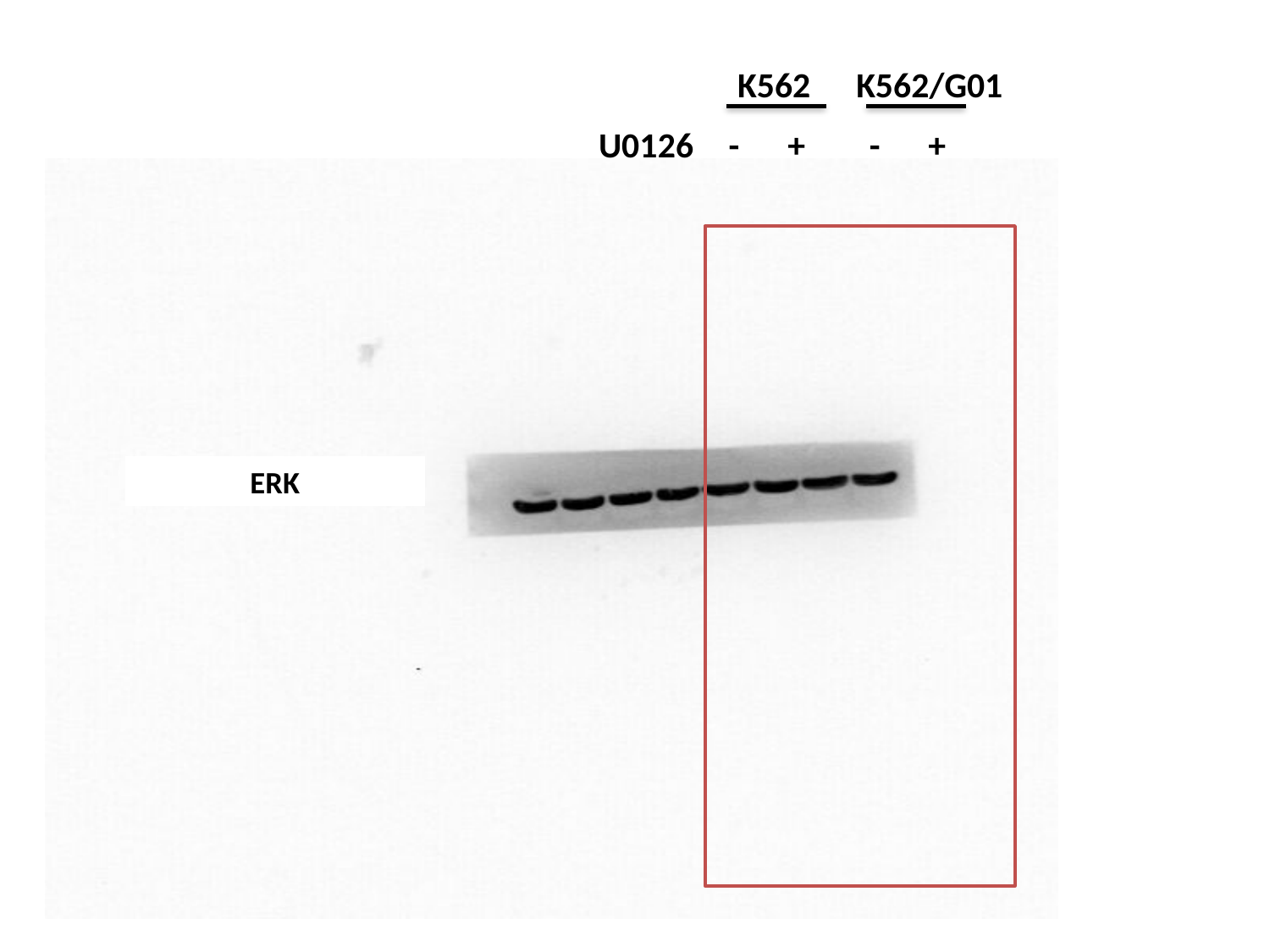

K562
K562/G01
U0126
- + - +
ERK

## Slide 5
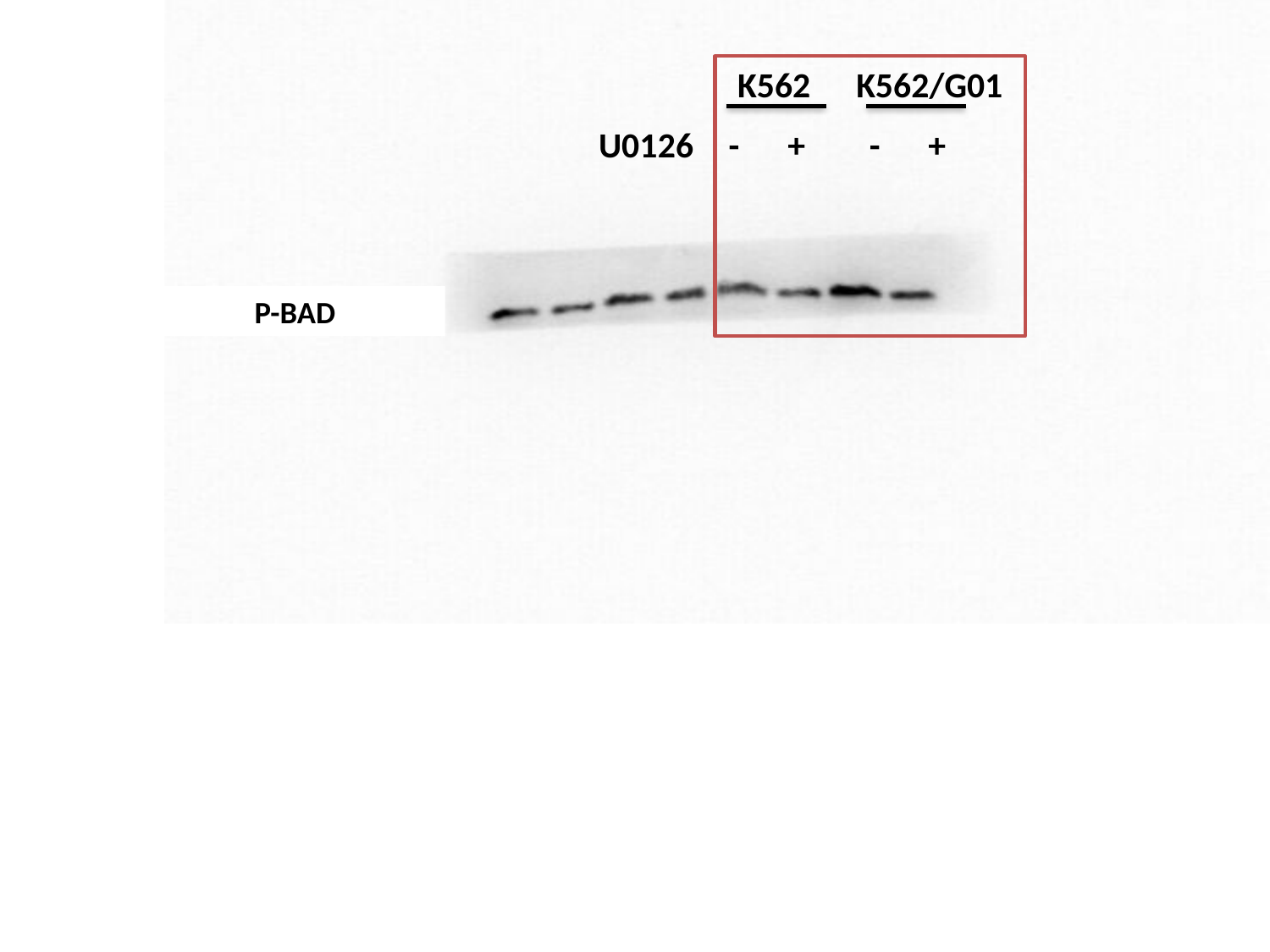

K562
K562/G01
U0126
- + - +
P-BAD

## Slide 6
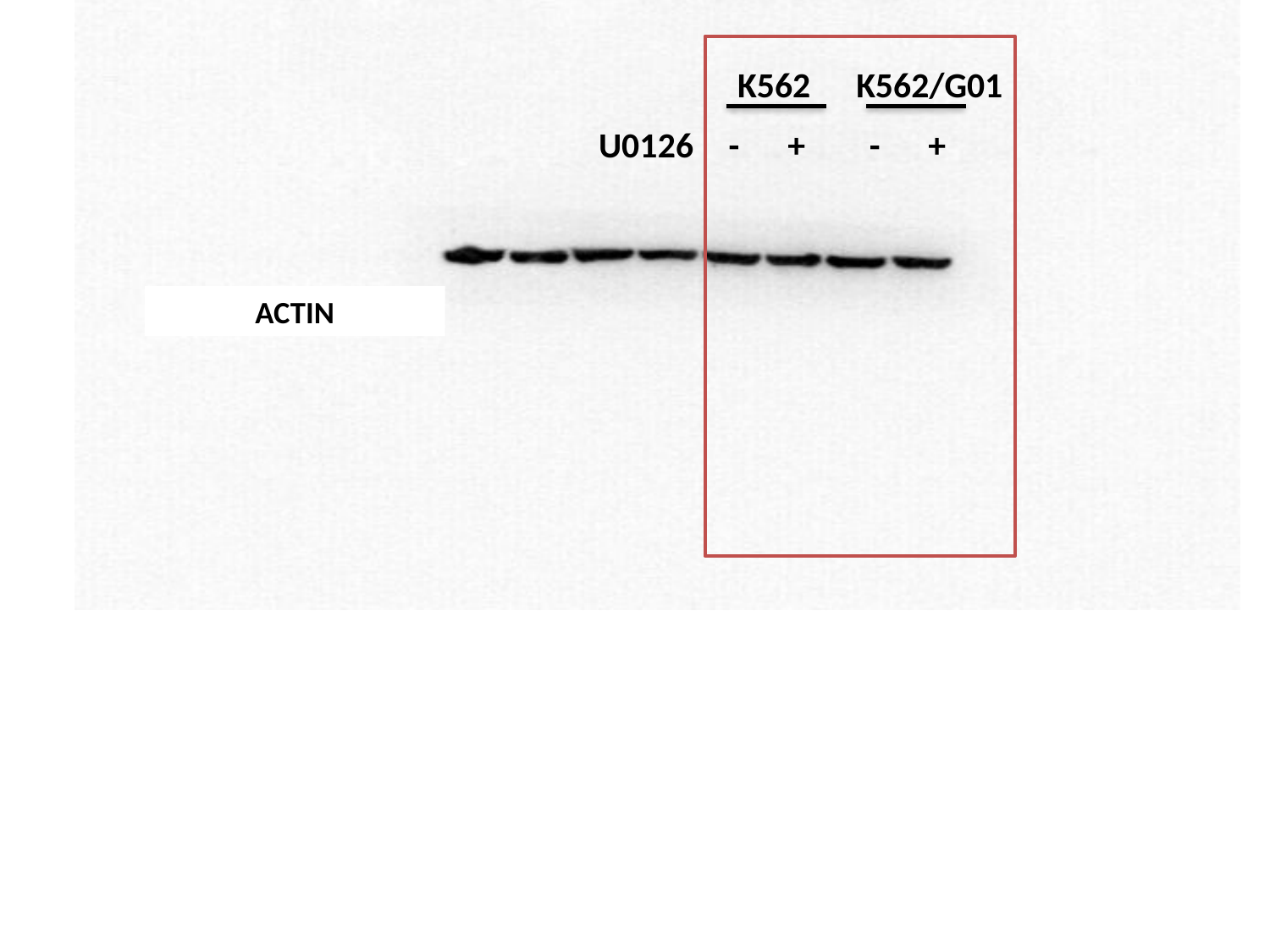

K562
K562/G01
U0126
- + - +
ACTIN
